# Ubiquitination and Deubiquitination: From Molecular Mechanisms to Therapeutic Strategies

**DOI:** 10.1002/mco2.71136

**Published:** 2026-11-13

**Authors:** Wanqing Liu, Jinglai Zhu, Dean Guo, Tao Yang, Tong Zhang, Zizhao Yang

**Affiliations:** ^1^ School of Pharmacy, Shanghai University of Traditional Chinese Medicine Shanghai China; ^2^ Center For Laboratory Animal Service & Experiments The Seventh People's Hospital of Shanghai University of Traditional Chinese Medicine Shanghai China; ^3^ Shanghai International Standardization Research Institute of Traditional Chinese Medicine, Shanghai University of Traditional Chinese Medicine Shanghai China

**Keywords:** deubiquitinating enzymes, deubiquitination, E3 ubiquitin ligases, molecular glues, PROTACs, targeted protein degradation, ubiquitin code, ubiquitination

## Abstract

Ubiquitination and deubiquitination are established as a reversible regulatory system controlling protein stability, localization, molecular interactions, and signaling duration. Ubiquitin is recognized not merely as a proteasomal degradation signal but as a molecular code whose output is determined by substrate identity, chain topology, and cellular context. Distinct ubiquitin signals are generated by E3 ubiquitin ligases (E3 ligases), remodeled by deubiquitinating enzymes (DUBs), and decoded by ubiquitinbinding domains. Beyond protein turnover, epigenetic and epitranscriptomic factors are directly regulated by ubiquitin signaling through control of their stability, activity, and interactions, thereby integrating chromatin and RNA regulation. In this review, the ubiquitin activating enzyme (E1)/ubiquitin conjugating enzyme (E2)/E3 cascade, ubiquitin chain architectures, DUBs, and signal recognition mechanisms are outlined in relation to key biological processes. The contribution of disrupted networks to cancer, neurodegeneration, and autoimmune diseases is discussed. Therapeutic strategies, including proteolysis targeting chimeras (PROTACs), molecular glues, DUB inhibitors, and E3 modulators, are presented with early clinical and preclinical developments highlighted to link mechanistic biology to therapeutic design. Shared principles of degradative and nondegradative ubiquitin signaling are identified across cellular networks, with emphasis on epigenetic and epitranscriptomic regulation. A nuanced framework is offered to interpret disease dysfunction, evaluate targeted therapies, and prioritize unresolved questions regarding substrate specificity and signaling context.

## Graphical abstract

Ubiquitination and deubiquitination form a reversible regulatory network that controls protein stability, localization, interactions, chromatin association, and signaling. E3 ligases generate distinct ubiquitin signals; DUBs remove or remodel them; and ubiquitin‐binding proteins decode chain topology and cellular context. This coordinated ubiquitination/deubiquitination network regulates DNA repair, epigenetic stability, immunity, inflammation, the cell cycle, apoptosis, and RNA metabolism. Its dysregulation contributes to cancer, neurodegeneration, and inflammatory or autoimmune disorders. Emerging therapeutics include PROTACs, molecular glues, DUB inhibitors, and targeted E3 modulators.

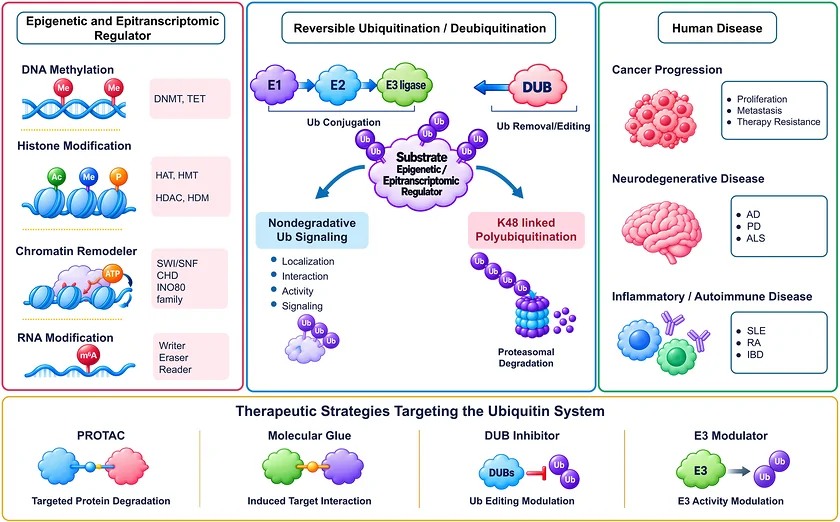

## 1 Introduction

Epigenetic regulation comprises heritable, reversible changes in gene activity without altering the DNA sequence. Since Waddington's foundational work, the field has evolved into a framework for understanding how chromatin states and cellular identities are established and maintained [1]. DNA methylation, histone modifications, chromatin remodeling, and RNAmediated regulation collectively shape chromatin accessibility, transcription, cell fate, and adaptation [2, 3]. Their dysregulation contributes to cancer and neurological disorders, driving the development of DNA methyltransferase and histone deacetylase (HDAC) inhibitors [4, 5]. Specifically, E3mediated ubiquitination targets DNA methyltransferase 1 (DNMT1) for proteasomal degradation, while ubiquitin specific protease 7 (USP7)mediated deubiquitination stabilizes DNMT1 [6].

Ubiquitination and deubiquitination form a reversible regulatory system: E3 ubiquitin ligases (E3 ligases) generate substratespecific ubiquitin signals, deubiquitinating enzymes (DUBs) remove or remodel them, and ubiquitinbinding proteins recognize distinct ubiquitin structures [7, 8, 9, 10, 11, 12, 13]. Ubiquitination can trigger proteasomal degradation or induce nondegradative changes, such as altered protein localization, activity, interactions, or signaling complex assembly, and serves as a cargo recognition signal in selective autophagy [14, 15].

At the epigenetic and epitranscriptomic interface, regulation by ubiquitination and deubiquitination can alter protein abundance, chromatin recruitment, catalytic activity, and multiprotein complex assembly [6, 16, 17, 18, 19]. Although extensive studies have characterized individual ubiquitindependent regulatory mechanisms, their relationships with chromatin and RNA regulation warrant further comparative synthesis to better understand how ubiquitin signals integrate epigenetic and epitranscriptomic processes.

The therapeutic relevance of this system is exemplified by proteolysistargeting chimeras (PROTACs) and molecular glues, which recruit or redirect E3 ligases to target proteins [20, 21, 22, 23]. Related strategies target key epigenetic regulators, including embryonic ectoderm development (EED) in polycomb repressive complex 2 (PRC2), DNMT1, and the methyltransferaselike 3/14 (METTL3/METTL14) methyltransferase complex [24, 25, 26]. Although target selection and cellular context remain critical considerations [27, 28, 29], DUB inhibitors and direct E3 ligase modulators offer complementary strategies to reshape protein stability and signaling [30, 31, 32].

Complementing established reviews on ubiquitin chain architecture, DUB specificity, selective autophagy, and therapeutic targeting, this review asks: how do ubiquitin signal generation, editing, and recognition control the abundance and functional states of regulatory proteins, and how can this knowledge guide therapy? Rather than cataloging enzymes and substrates, we use epigenetic and epitranscriptomic regulators as recurring examples to compare outcomes across cellular networks: degradation, stabilization, altered activity, localization, recruitment, and molecular interactions. DNA repair, chromatin regulation, RNA modification, immune signaling, and selective autophagy are integrated, not as isolated topics, but as illustrations of shared principles: substrate selection, ubiquitin chain topology, DUBmediated editing, signal recognition, and cellular context. This framework directly links disease dysregulation to mechanistic rationales for targeted protein degradation, DUB inhibition, and E3 modulation.

Accordingly, this review first outlines the core mechanisms of ubiquitin conjugation, editing, and recognition; then examines their roles in epigenetic and epitranscriptomic regulation alongside broader cellular networks; discusses their pathological implications; evaluates therapeutic strategies; and concludes with key challenges and future directions.

## 2 UbiquitinProteasome System and DUBs: Mechanistic Overview

The ubiquitin system generates, edits, and decodes structurally diverse signals through the coordinated actions of ubiquitinactivating enzyme (E1), ubiquitinconjugating enzyme (E2), E3 ligases, DUBs, and ubiquitin‐binding proteins. This section outlines the ubiquitination cascade, ubiquitin chain architectures, major families of DUBs, and signal recognition mechanisms, the foundation for the functional networks discussed below.

### 2.1 The Ubiquitination Cascade: E1, E2, and E3 Enzymes

Ubiquitination is initiated through a sequential enzymatic cascade involving E1, E2, and E3 ligases. While E1 and E2 enzymes participate in ubiquitin activation and transfer, E3 ligases provide the primary determinant of substrate specificity by selecting target proteins and shaping ubiquitin signal architecture [8, 33, 34]. Based on catalytic mechanisms, E3 ligases are broadly classified into really interesting new gene (RING), homologous to E6AP C terminus (HECT), and RING between RING (RBR) families, which differ in ubiquitin transfer strategies. RING E3 ligases facilitate direct ubiquitin transfer from E2 to substrates, whereas HECT and RBR E3 ligases form transient ubiquitin thioester intermediates before ubiquitin transfer [33, 34].

E2/E3 pairing further influences ubiquitin transfer efficiency, chain initiation, elongation, and linkage selection. RING E3 ligases include both single subunit enzymes and multisubunit cullin RING ligase complexes, whereas RBR ligases, exemplified by Parkin and HHARI (ARIH1), employ a hybrid catalytic mechanism involving distinct E2 binding and catalytic domains [35].

Together, these reactions generate monoubiquitination, multi‐monoubiquitination, and structurally diverse polyubiquitin chains whose consequences are determined by chain architecture, substrate context, and signal recognition.

### 2.2 The Ubiquitin Code: Chain Topology and Substrate Fate

The ubiquitin code describes how structurally distinct ubiquitin modifications encode signals that are interpreted to produce cellular outcomes. Ubiquitin can be attached as a single unit (monoubiquitination), at multiple sites on a substrate (multi‐monoubiquitination), or as chains (polyubiquitination). Polyubiquitin chains can be assembled through ubiquitin's seven lysine residues or N terminal methionine (M1), generating homotypic, mixed, and branched architectures with distinct signaling properties [36, 37].

K48linked polyubiquitin is a canonical signal for recognition and degradation by the 26S proteasome [9, 38], while K11linked chains frequently support anaphasepromoting complex/cyclosome (APC/C)dependent turnover during cell cycle progression [39]. K63linked chains more commonly organize nonproteolytic processes, including DNA damage responses (DDRs), immune signaling, endocytic trafficking, and signaling complex assembly [11, 12, 36, 37]. Ubiquitinated cargo can also recruit ubiquitinbinding adaptors and autophagy receptors for selective autophagic clearance [14, 40, 41]. These associations are not absolute, because K63linked chains can seed branched architectures that promote proteasomal degradation in specific contexts [42].

M1linked linear ubiquitin chains are generated by the linear ubiquitin chain assembly complex (LUBAC) and have major roles in inflammatory and immune signaling, particularly in nuclear factor κB (NFκB) pathway assembly [43]. Atypical K6, K27, K29, and K33linked chains contribute to diverse processes, including genome maintenance, immune regulation, and protein homeostasis [36, 37]. Ring finger protein 168 (RNF168)mediated K27linked ubiquitination of H2A family histones, for example, contributes to the recruitment of DDR factors to damaged chromatin [44].

Mixed chains contain more than one linkage type, while branched chains contain ubiquitin moieties modified at multiple sites. K11/K48 branched chains enhance the proteasomal degradation of mitotic regulators, whereas other mixed or branched architectures can alter receptor recruitment or integrate proteolytic and nonproteolytic signals [42, 45]. Thus, linkage identity influences substrate fate but does not determine it in isolation; chain length, architecture, substrate context, localization, and signal decoding act together.

### 2.3 DUBMediated Counterbalance: Classification and Catalytic Mechanisms

DUBs are essential counter regulators of ubiquitination. They remove ubiquitin from substrates, trim or remodel polyubiquitin chains, process ubiquitin precursors, and recycle ubiquitin, thereby maintaining ubiquitin homeostasis and limiting inappropriate signaling [10, 46]. Through these functions, DUBs control the magnitude, duration, reversibility, and specificity of ubiquitin‐dependent regulation.

Human DUBs are classified into several major families, including ubiquitin‐specific proteases (USPs), ubiquitin C terminal hydrolases (UCHs), ovarian tumor (OTU) domain containing proteases, Machado/Joseph disease domain containing proteases (Josephins), and JAB1/MPN/MOV34 metalloenzyme (JAMM) family metalloproteases [46, 47]. Beyond ubiquitin removal, DUBs regulate ubiquitin signaling by controlling substrate stability, ubiquitin chain architecture, localization, and protein activity. Cysteine protease DUBs use catalytic Cys, His, and Asp/Asn residues; JAMM DUBs rely on a metal‐dependent catalytic center. Substrate selection and linkage preference are further determined by catalytic domain architecture, ubiquitinbinding surfaces, auxiliary domains, interaction partners, and subcellular localization.

DUB specificity can depend on both linkage type and chain length. Breast cancer Type 1 susceptibility protein (BRCA1)associated protein 1 (BAP1) cooperates with ASXL proteins to remove monoubiquitin from H2AK119, BRCA1/BRCA2 containing complex subunit 3 (BRCC36/BRCC3) preferentially cleaves K63linked chains, and MINDY1 preferentially recognizes and cleaves extended K48linked chains [48, 49, 50]. These examples illustrate how catalytic organization and auxiliary recognition elements shape DUB specificity and ubiquitin signal editing.

Mechanistic studies further show that DUBs can stabilize substrates, remove activating monoubiquitin marks, remodel polyubiquitin chains, or suppress ubiquitin transfer through noncatalytic mechanisms [10, 19, 51, 52, 53]. Accordingly, DUBs function as active editors of the ubiquitin code, and their effects depend on the substrate, ubiquitin architecture, interacting partners, and cellular context.

### 2.4 Ubiquitin Signal Recognition

Ubiquitinbinding proteins decode ubiquitin signals through noncovalent ubiquitinbinding domains, which recognize specific chain architectures and link ubiquitination events to downstream cellular responses [40]. Individual domains vary in their affinity for monoubiquitin and polyubiquitin and in their preference for linkages or topologies. Because many individual interactions are weak, avidity and multivalent recognition arise from tandem domains, domain spacing and orientation, and cooperation with adjacent structural modules.

Proteasomal receptors provide a central example of ubiquitin signal recognition. Rpn10/S5a recognizes ubiquitin chains through ubiquitininteracting motifs (UIMs), whereas Rpn13 uses an N terminal pleckstrin like receptor for ubiquitin (Pru) domain. Shuttle proteins such as RAD23 capture ubiquitinated cargo through ubiquitinassociated (UBA) domains and dock at the proteasome through ubiquitin like domains, coupling chain recognition to substrate delivery and processing by the 26S proteasome [9, 38, 54].

Signaling adaptors use related principles to decode nonproteolytic chains. At DNA double strand breaks (DSBs), the tandem UIMs of receptorassociated protein 80 (RAP80) preferentially recognize K63linked chains and recruit the BRCA1 complex, whereas the ubiquitinbinding in ABIN and NEMO (UBAN) domain of NEMO preferentially recognizes M1linked ubiquitin chains during IκB kinase (IKK)/NFκB signaling [55, 56, 57]. In chromatin regulation, a ubiquitininteracting region in DNA methyltransferase 3A (DNMT3A) isoform 1 (DNMT3A1) recognizes H2AK119Ub and contributes to recruitment of de novo DNA methylation machinery to polycombregulated chromatin [58].

Autophagy receptors also decode ubiquitin as a cargo selection signal. Sequestosome 1/p62 (p62/SQSTM1) and neighbor of BRCA1 gene 1 (NBR1) use C terminal UBA domains to bind ubiquitinated cargo and microtubuleassociated protein 1 light chain 3 (LC3)interacting regions to engage LC3/gamma aminobutyric acid receptorassociated protein (GABARAP) proteins on autophagosomal membranes [14, 15, 41]. Histone Deacetylase 6 (HDAC6) uses its zinc finger ubiquitinbinding (ZnF UBP) domain to recognize unanchored ubiquitin C termini associated with protein aggregates and promote aggresome recruitment, acting as a ubiquitinbinding adaptor rather than a canonical LC3binding autophagy receptor [59].

Together, proteasomal receptors, signaling adaptors, chromatin readers, and autophagy receptors show that ubiquitindependent outcomes require both signal architecture and recognition. The coordinated generation, editing, and decoding of ubiquitin signals provide the mechanistic basis for the functional networks discussed in the following sections.

## 3 Regulatory Roles of Ubiquitination and Deubiquitination in Major Cellular Networks

Ubiquitination and deubiquitination coordinate diverse cellular processes by regulating protein stability, enzymatic activity, and molecular interactions. Degradationassociated ubiquitination and DUBmediated editing modulate the abundance of regulatory proteins, whereas nondegradative ubiquitin signals facilitate the recruitment of DNA repair and signaling complexes and influence protein activity. Through these distinct mechanisms, ubiquitin signaling contributes to genome maintenance and chromatin regulation, inflammatory and immune responses, cell cycle progression and apoptosis, and RNA modification and editing. The resulting cellular outcomes depend on substrate identity, ubiquitin chain architecture, cellular context, and recognition by ubiquitinbinding proteins [10, 11]. Across the following subsections, we compare representative examples in terms of the underlying ubiquitindependent mechanism, its consequences for protein abundance or function, and the biological context in which these effects occur. This comparative approach highlights shared regulatory principles while distinguishing degradative and nondegradative outcomes across cellular networks (Figure 1).

**FIGURE 1:**
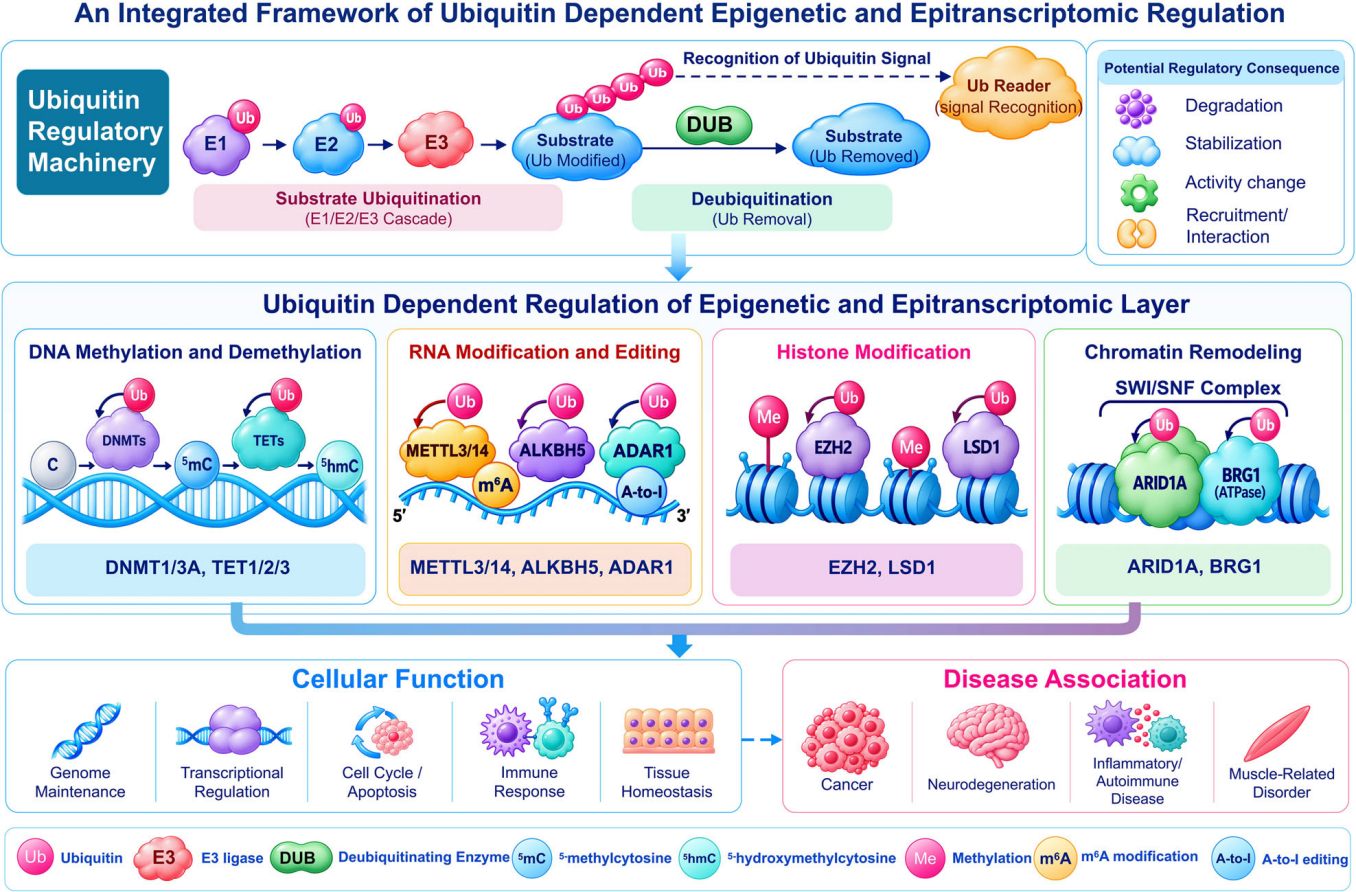
An integrated framework of ubiquitindependent epigenetic and epitranscriptomic regulation. The ubiquitin system, comprising E1/E2/E3 ligases, deubiquitinating enzymes (DUBs), and ubiquitin readers, controls substrate fate, activity, and interactions. It regulates four key epigenetic and posttranscriptional layers: DNA methylation/demethylation, RNA modification/editing, histone modification, and chromatin remodeling. These mechanisms govern essential cellular functions, including genome maintenance, transcription, cell cycle control, immunity, and tissue homeostasis, and are implicated in cancer, neurodegeneration, inflammatory/autoimmune diseases, and muscle disorders.

In genome and chromatinassociated processes, ubiquitin signals serve two related roles: assembling nondegradative platforms for DNA repair and regulating the abundance or chromatin engagement of epigenetic factors. RING finger protein 8 (RNF8) and RNF168 promote the recruitment of repair proteins, including p53binding protein 1 (53BP1) and BRCA1 containing complexes [60], whereas E3 ligases and DUBs modulate DNA methyltransferases, ten eleven translocation (TET) dioxygenases, and chromatin remodeling proteins.

Ubiquitination/deubiquitination networks are similarly central to inflammatory signaling, immune activation, cell cycle progression, and apoptosis. In innate immune pathways, nondegradative ubiquitin chains frequently act as molecular scaffolds for signalosome assembly. A classic example is tumor necrosis factor (TNF) receptorassociated factor 6 (TRAF6)dependent activation of the IKK complex, which requires the Ubc13/Uev1A ubiquitinconjugating system and polyubiquitin chain formation [61]. By contrast, cell cycle and apoptotic pathways often depend on tightly timed degradation or stabilization of regulatory proteins. In the tumor protein p53 (p53)/mouse double minute 2 homolog (MDM2) axis, MDM2 promotes p53 ubiquitination and degradation, whereas DUBs such as USP7, USP10, and USP11 modulate the stability and activity of p53 or its negative regulators [62, 63]. Thus, the balance between ubiquitin ligases and DUBs helps determine whether cells proliferate, arrest, repair damage, activate inflammation, or undergo programmed cell death.

Ubiquitination and deubiquitination also regulate RNA metabolism and epitranscriptomic programs. N^6^‐methyladenosine (m^6^A) writers, erasers, and readers can themselves be substrates of E3 ligases and DUBs, linking protein stability to RNA methylation, RNA decay, translation, stress adaptation, and disease‐associated transcriptional programs. For example, USP5 stabilizes extracellular signalregulated kinase (ERK) phosphorylated METTL3 and supports m^6^A methyltransferase complex activity, whereas E3 ligases can promote the degradation of METTL3, METTL14, AlkB homolog 5 (ALKBH5), and selected m^6^A readers in a contextdependent manner [19, 64]. In specific settings, ubiquitinated proteins may additionally be routed toward selective autophagy through receptors such as p62/SQSTM1, providing an auxiliary ubiquitindependent pathway for the removal of aggregated, damaged, or poorly soluble substrates [15]. The following sections discuss how these interconnected ubiquitination/deubiquitination networks regulate DNA damage repair and epigenetic stability, inflammatory and immune signaling, cell cycle progression and apoptosis, and RNA metabolism (Table 1).

**TABLE 1:** Key epigenetic and epitranscriptomic enzymes and their degradation pathways.

Enzymes	Encoded genes	Epigenetics	Protein degradation pathways	Selective autophagy receptors
DNMT1 [6, 65, 66, 67]	DNMT1	DNA methylation	Proteasomal pathway	/
DNMT3A [68, 69, 70]	DNMT3A	DNA methylation	Proteasomal pathway	/
TET2 [71, 72, 73, 74, 75]	TET2	DNA demethylation	Proteasomal pathway	/
EZH2 [17, 76]	EZH2	Histone modification	Proteasomal pathway	/
MLL1 [77, 78, 79]	KMT2A	Histone modification	Proteasomal pathway	/
HDAC4 [80]	HDAC4	Histone modification	Proteasomal pathway	/
SMARCA4 [81, 82]	SMARCA4	Chromatin remodeling	Proteasomal pathway	/
ARID1A [83, 84, 85]	ARID1A	Chromatin remodeling	Proteasomal pathway	/
CHD4 [86, 87]	CHD4	Chromatin remodeling	Proteasomal pathway	/
METTL3 [88, 89, 90]	METTL3	RNA modification/m^6^A	Proteasomal pathway	/
METTL14 [91, 92]	METTL14	RNA modification/m^6^A	Proteasomal pathway (Including ERAD)	/
WTAP [93]	WTAP	RNA modification/m^6^A	Proteasomal pathway	/
FTO [94, 95, 96, 97]	FTO	RNA modification/m^6^A	Proteasomal pathway; p62/SQSTM1‐mediated selective autophagy	p62/SQSTM1 [96, 97]
ALKBH5 [98, 99, 100, 101]	ALKBH5	RNA modification/m^6^A	Proteasomal pathway	/
NAT10 [102, 103]	NAT10	RNA modification/ac^4^C	Proteasomal pathway	/
ADAR1 [104, 105]	ADAR1	RNA editing/A‐to‐I	Proteasomal pathway	/

### 3.1 Regulation of DNA Damage Repair and Epigenetic Stability

DNA DSB repair provides a canonical example of ubiquitindependent chromatin signaling. Following DNA damage, phosphorylation of mediator of DNA damage checkpoint protein 1 (MDC1) creates docking sites for the FHA domain of RNF8, allowing RNF8 to initiate ubiquitindependent signaling at damaged chromatin and promote the recruitment of RNF168 [106]. RNF168 subsequently amplifies the response by monoubiquitinating H2A and H2AX at Lys13 and Lys15, thereby promoting 53BP1 accumulation and the assembly of DDR foci [107]. In parallel, RAP80 recognizes nonK48 ubiquitin structures, particularly K63linked chains, through tandem UIMs and recruits the BRCA1‐A complex (BRCA1‐A) to DNA lesions [55]. The BRCA1‐A‐associated JAMM family DUB BRCC36/BRCC3 counterbalances this pathway by editing K63linked ubiquitin chains and modulating the extent and duration of damageassociated ubiquitin signaling [49]. Together, these events demonstrate that chromatin ubiquitination can function as a spatial recruitment and signaling mechanism without necessarily targeting the modified substrate for degradation.

Replicationassociated DNA damage is controlled by a ubiquitin editing network centered on proliferating nuclear antigen (PCNA) and the Fanconi anemia pathway. At stalled replication forks, the RAD6/RAD18 E2/E3 complex monoubiquitinates PCNA at Lys164, facilitating the recruitment of translesion DNA polymerases and lesion bypass [108, 109]. In parallel, monoubiquitination of the Fanconi anemia complementation group I (FANCI)/Fanconi anemia group D2 protein (FANCD2) complex promotes its retention at damaged chromatin and is required for efficient DNA interstrand crosslink repair [110]. The USP1/USP1associated factor 1 (UAF1) deubiquitinase complex subsequently removes monoubiquitin from PCNA and FANCD2, helping terminate or reset these DNA damage tolerance and repair responses [111, 112, 113]. Importantly, failure to install these ubiquitin signals compromises lesion bypass and crosslink repair, whereas failure to deubiquitinate FANCD2 also impairs efficient repair, demonstrating that genome maintenance depends on the dynamic installation and removal of ubiquitin modifications rather than on ubiquitination alone.

Alongside its role in DNA repair, ubiquitin signaling influences epigenetic stability by regulating the abundance, chromatin engagement, and activity of DNA methylation and demethylation factors. DNMT3A and DNMT3B establish de novo methylation patterns, whereas DNMT1 maintains DNA methylation during replication [114, 115, 116]. TET family dioxygenases promote DNA demethylation through methylcytosine oxidation [117, 118]. Importantly, ubiquitination and deubiquitination regulate the stability and chromatin engagement of these enzymes: coordinated E3/DUB activities control DNMT1 turnover, whereas cullin/RING ligase 4 Vprbinding protein complex (CRL4^VprBP^)mediated monoubiquitination promotes TET chromatin binding [6, 16]. These mechanisms contribute to methylation fidelity and disease‐associated epigenetic remodeling [119].

#### 3.1.1 DNMT1

DNMT1 provides a representative example of how degradative, and nondegradative ubiquitin signals cooperate to maintain epigenetic inheritance. Its recruitment to newly replicated chromatin depends on ubiquitin like with PHD and RING finger domains 1 (UHRF1), whose SET and RINGassociated domain recognizes hemimethylated DNA and facilitates DNMT1 recruitment and activation [120]. Accordingly, the coordinated regulation of DNMT1 abundance, chromatin recruitment, and catalytic activity is crucial for faithful maintenance of DNA methylation.

DNMT1 abundance is controlled by several ubiquitindependent proteolytic mechanisms. 60 kDa Tat interactive protein (TIP60)mediated acetylation of DNMT1 facilitates its recognition and ubiquitination by the E3 ligase UHRF1, leading to proteasomal degradation, whereas HDAC1mediated deacetylation and USP7mediated deubiquitination counteract this process and stabilize DNMT1 [6]. DNMT1 turnover is also coupled with lysine methylation. Methylation of DNMT1 at Lys142 creates a recognition signal for L3MBTL3, which recruits the cullin/RING ligase 4 DCAF5 complex (CRL4^DCAF5^) ubiquitin ligase and promotes DNMT1 proteolysis. Conversely, lysinespecific demethylase 1 (LSD1)mediated demethylation and PHD finger protein 20 like 1 (PHF20L1) binding protect DNMT1 from this degradation pathway, particularly during S phase [65]. These findings demonstrate that crosstalk among acetylation, methylation, ubiquitination, and deubiquitination determines DNMT1 stability rather than ubiquitination acting as an isolated degradation signal.

Diseaseassociated alterations in E3 ligase activity can further reshape DNMT1 abundance and its downstream methylation output. In gastric cancer (GC), Protocadherin 10 (PCDH10) is downregulated in tumor tissues and cell lines, and low PCDH10 expression is associated with lymph node metastasis and poor prognosis. RNF180 directly interacts with DNMT1 and promotes its ubiquitination and proteasomal degradation, thereby reducing excessive methylation of the PCDH10 promoter, restoring PCDH10 expression, and suppressing tumor cell proliferation, migration, invasion, and metastatic potential [66]. Metabolic stress provides a distinct example. In high fat diet fed mice, reduced hepatic ubiquitin protein ligase E3A (UBE3A) expression decreases DNMT1 ubiquitination and proteasomal turnover, resulting in increased DNMT1 stability. Stabilized DNMT1, together with DNMT3A, promotes methylation of the Klb promoter and reduces Klb expression, whereas liverspecific deletion of DNMT1 or DNMT3A restores Klb expression and ameliorates hepatic steatosis [67]. Thus, diseaseassociated changes in E3 ligasedependent DNMT1 turnover can convert oncogenic or environmental signals into persistent alterations in DNA methylation and gene expression.

#### 3.1.2 UHRF1

UHRF1 also regulates DNMT1 through nondegradative ubiquitin signaling. After recognizing hemimethylated DNA and the surrounding chromatin environment, the RING domain of UHRF1 catalyzes multi‐monoubiquitination of the H3 N terminal tail. Monoubiquitination at H3 Lys18 and Lys23 generates a major dual monoubiquitin signal, H3Ub2, which is recognized by the replication foci targeting sequence domain of DNMT1. This interaction promotes DNMT1 recruitment to chromatin, relieves DNMT1 autoinhibition, and enhances its methyltransferase activity [121, 122, 123]. UHRF1 additionally catalyzes dual monoubiquitination of PCNAassociated factor 15, generating PAF15Ub2 as a replication coupled DNMT1 recruitment signal. PAF15Ub2 acts predominantly during early S phase, whereas H3Ub2 provides a complementary recruitment mechanism at later replicating regions; together, these pathways support high fidelity inheritance of DNA methylation [124]. Thus, UHRF1mediated ubiquitination can either promote DNMT1 degradation or generate chromatin docking and activation signals for DNMT1, depending on the modified substrate and molecular context.

The stability and subcellular localization of UHRF1 are themselves dynamically regulated by ubiquitination, phosphorylation, and deubiquitination. In cholangiocarcinoma (CCA), STIP1 homology and U box containing protein 1 (STUB1) expression is elevated in tumor tissues, and high STUB1 expression is associated with aggressive disease and poor overall survival. Functional studies showed that STUB1 promotes CCA cell proliferation, migration, invasion, and tumor growth, whereas phospholipase A2 group IIA (PLA2G2A) acts as a tumor suppressor in this context [125]. Mechanistically, STUB1 catalyzes nondegradative K63linked ubiquitination of UHRF1, enhancing its nuclear localization and interaction with DNMT1. The resulting STUB1/UHRF1/DNMT1 complex increases methylation of the PLA2G2A promoter and represses PLA2G2A expression, thereby connecting altered ubiquitin signaling with clinically relevant epigenetic silencing [125]. In a distinct pathway, polo like kinase 1 (PLK1) phosphorylates UHRF1 at Ser265, facilitating its interaction with USP7 and promoting USP7dependent deubiquitination and stabilization of UHRF1. PLK1 inhibition disrupts this interaction, increases ubiquitinproteasomedependent UHRF1 turnover, reduces DNMT1 recruitment to chromatin, and decreases global DNA methylation [126]. These examples demonstrate that ubiquitin signaling controls maintenance methylation both by determining DNMT1 abundance and by regulating the stability, localization, and chromatin scaffolding functions of UHRF1.

Collectively, the UHRF1/DNMT1 axis illustrates the functional diversity and context dependence of ubiquitin signaling in epigenetic regulation. Degradationoriented ubiquitination restrains DNMT1 or UHRF1 abundance, whereas substratespecific monoubiquitination and K63linked ubiquitination promote DNMT1 recruitment, activation, or assembly with regulatory partners. Disruption of these interconnected mechanisms can lead to either excessive DNMT1dependent gene silencing or insufficient maintenance methylation, emphasizing that epigenetic stability requires coordinated control of protein turnover, chromatinassociated ubiquitin signaling, and downstream proteostasis programs.

#### 3.1.3 DNMT3A

DNMT3A links ubiquitin signaling to de novo DNA methylation, chromatin targeting, protein turnover, and disease‐associated transcriptional reprogramming. As a principal de novo DNA methyltransferase, DNMT3A establishes new methylation patterns during embryonic development, lineage differentiation, and tissue maturation and contributes to the regulation of gene expression, transposable element repression, and genome stability [116]. DNMT3A mutations are recurrent in acute myeloid leukemia (AML), highlighting the importance of DNMT3A dysfunction in abnormal hematopoietic differentiation and leukemic transformation [127]. Accordingly, regulation of DNMT3A abundance, catalytic activity, oligomerization, and chromatin localization can have substantially different consequences depending on the affected cell type and disease context.

A major nondegradative connection between DNMT3A and ubiquitin signaling is provided by its recognition of monoubiquitinated H2A. The long DNMT3A1 isoform contains an intrinsically disordered N terminal region that recognizes H2AK119Ub, enabling DNMT3A1 recruitment to polycomb repressive complex 1 (PRC1) associated and bivalent chromatin [58, 128]. This pathway functions alongside chromatin targeting mediated by the DNMT3A Pro Trp Trp Pro (PWWP) domain. When normal PWWPdependent targeting is impaired, DNMT3A can be redistributed toward PRC1regulated CpG islands, altering the timing and distribution of de novo DNA methylation. Disruption of the DNMT3A1 N terminal H2AK119Ub recognition region similarly perturbs methylation at developmental genes and produces postnatal developmental abnormalities. Thus, H2AK119Ub acts as a chromatin docking signal for DNMT3A rather than as a degradation mark, extending the regulatory function of histone ubiquitination from transcriptional repression to locus selective DNA methylation.

DNMT3A abundance is also controlled by ubiquitin‐dependent proteolysis. Systematic functional analysis of disease‐associated DNMT3A variants identified a substantial group of loss of function variants with reduced protein stability. These unstable DNMT3A variants are recognized by DDB1 and CUL4 associated factor 8 (DCAF8), the substrate receptor of the Cullin 4 (CUL4)/damage specific DNA binding protein 1 (DDB1) E3 ligase complex, and subsequently undergo ubiquitination and proteasomal degradation [68]. Genetic or pharmacological disruption of the DCAF8 pathway increases the abundance of instability prone DNMT3A variants, establishing regulated protein turnover as an important determinant of DNMT3Aassociated developmental disorders, clonal expansion, and leukemic risk. However, protein instability is not a universal property of pathogenic DNMT3A mutations. The recurrent AMLassociated R882H mutation primarily disrupts DNMT3A oligomerization and blocks formation of catalytically active tetramers, thereby exerting dominant negative effects on wildtype (WT) DNMT3A and producing abnormal methylation patterns [129]. The R882 mechanism should therefore be distinguished from the DCAF8sensitive class of destabilized DNMT3A variants rather than described as defective ubiquitinmediated degradation.

Additional context‐dependent pathways couple DNMT3A turnover to signaling and cell state transitions. In vascular smooth muscle cells, nuclear focal adhesion kinase (FAK) associates with the E3 ligase TRAF6 and DNMT3A, promoting DNMT3A ubiquitination and proteasomal degradation. Following vascular injury, activated FAK is redistributed toward the cytoplasm, weakening this nuclear degradation pathway and allowing DNMT3A to accumulate. Increased DNMT3A then enhances methylation of smooth muscle contractile gene promoters and promotes cellular dedifferentiation. Conversely, inhibition of FAK catalytic activity favors nuclear FAK localization, increases TRAF6dependent DNMT3A degradation, reduces promoter methylation, and helps preserve the differentiated smooth muscle phenotype [69]. This mechanism demonstrates that FAK localization, rather than FAK activation alone, determines whether DNMT3A is subjected to ubiquitindependent degradation.

Viral infection provides another example of regulated DNMT3A destruction. The Newcastle disease virus V protein interacts with DNMT3A and promotes its K48linked ubiquitination and proteasomal degradation. Reduced DNMT3A abundance alters methylation across the interferon regulatory factor 7 (IRF7) locus, decreases IRF7 expression, and attenuates downstream IFN‐β production, thereby contributing to viral evasion of the host antiviral response [70]. Although this mechanism was defined in a virus/host experimental system and should not be generalized to all mammalian immune contexts, it provides direct evidence that pathogen encoded proteins can exploit DNMT3A ubiquitination to reprogram innate immune transcription.

DNMT3A's turnover also intersects with cell cycle signaling and autophagy. In advanced prostate cancer models, PLK1 phosphorylates DNMT3A and promotes its degradation during mitosis. During interphase, DNMT3A reciprocally represses PLK1 transcription and limits autophagic activity, establishing a negative feedback loop between DNMT3A abundance, PLK1 signaling, and autophagy [130]. Although the ubiquitin linkage type was not resolved, the study reported evidence implicating F‐box and WD repeat domain‐containing protein 7 (FBXW7) in PLK1associated DNMT3A polyubiquitination and proteasomal degradation. Nevertheless, the finding directly links regulated DNMT3A turnover to the control of an autophagic program and supports combined targeting of PLK1 and DNMT3A in the relevant prostate cancer models.

DNMT3Adependent methylation programs have contextspecific pathological consequences. In gallbladder cancer, DNMT3A cooperates with yesassociated protein (YAP)/transcriptional coactivator with PDZ binding motif (TAZ) to promote metastasisassociated methylation programs [131]. In breast cancer, DNMT3Amediated silencing of dermatopontin promotes YAP‐associated malignant progression [132]. In lung adenocarcinoma, DNMT3A can promote HDAC7/ZEB1/MYC proto‐oncogene protein (MYC; c‐Myc) signaling [133], whereas DNMT3A deficiency creates a dependency on KDM1A in a subset of non‐small cell lung cancer (NSCLC) [134]. These contrasting effects highlight the tissue and locus‐specific consequences of DNMT3A dysregulation.

Collectively, DNMT3A illustrates several distinct modes of ubiquitin and epigenetic crosstalk. H2AK119Ub provides a nondegradative chromatin recognition signal that directs DNMT3A1 toward polycombassociated loci, whereas DCAF8, TRAF6, and virusinduced ubiquitination regulate DNMT3A abundance through proteasomal turnover. Pathogenic DNMT3A variants may disrupt protein stability, catalytic activity, oligomerization, or chromatin targeting through mechanistically distinct routes. At a broader level, DNMT3A turnover can intersect with cell cycle and autophagy regulation, as illustrated by the PLK1dependent pathway described above. These findings do not establish selective autophagy as a general route for DNMT3A protein degradation. Instead, DNMT3A integrates degradative ubiquitination, nondegradative histone ubiquitin recognition, and cell cycle‐dependent protein turnover in a highly contextdependent manner.

#### 3.1.4 TET

TET provides a complementary model of how ubiquitination and deubiquitination can regulate epigenetic enzymes through both degradative and nondegradative mechanisms. TET1, TET2, and TET3 catalyze the iterative oxidation of ^5^‐methylcytosine and thereby contribute to DNA demethylation, transcriptional plasticity, lineage differentiation, and genome regulation. Their biological output depends not only on catalytic activity but also on protein abundance, chromatin recruitment, isoform usage, and cellular context. CRL4^VprBP^, composed of VprBP, DDB1, CUL4, and ROC1, binds all three TET proteins and monoubiquitinates a conserved lysine within their catalytic region. Rather than targeting TET proteins for degradation, this modification enhances their association with DNA and chromatin and supports methylcytosine oxidation [16]. Thus, monoubiquitination can activate TETdependent epigenetic regulation by promoting substrate engagement rather than protein turnover.

TET1 displays additional isoformspecific regulation by ubiquitin signaling. Fulllength TET1 contains an N terminal CXXC type zinc finger region that favors its retention at euchromatic and CpGrich regions, whereas the short TET1 (TET1s) isoform lacks this N terminal region. During late S phase, TET1s interacts with and is ubiquitinated by CRL4^VprBP^, after which ubiquitinated TET1s is recognized by UHRF1 and recruited to replicating heterochromatin [135]. This recruitment increases methylcytosine oxidation in heterochromatic regions, promotes chromatin decondensation, and can increase the expression of normally repressed repetitive elements, including long interspersed nuclear element‐1 (LINE‐1) sequences. In contrast, fulllength TET1 is restrained from the same heterochromatic compartment by its N terminal zinc finger region. These findings show that ubiquitination can determine the replication stage and isoformspecific localization of TET1 without changing its abundance.

Beyond serving as a direct ubiquitin substrate, TET1 can indirectly reprogram ubiquitin‐dependent turnover by controlling the expression or activity of E3 ligase pathways. Under iron overload conditions, TET1dependent demethylation increases RNF217 expression. RNF217 subsequently ubiquitinates the iron exporter ferroportin, promoting its degradation and thereby contributing to systemic iron homeostasis [136]. Pharmacological experiments indicated that ferroportin turnover in this setting can involve both proteasomal and lysosomal degradation. This represents an intersection between ubiquitindependent substrate recognition and two proteolytic compartments, although it does not establish canonical macroautophagy or selective autophagic degradation of TET1 itself. Loss of TET1 or RNF217 stabilizes ferroportin and impairs the normal physiological response to iron overload [136].

TET1 can also regulate the ubiquitination of downstream signaling proteins through catalytic activityindependent transcriptional mechanisms. In hypoxic thyroid cancer models, TET1 functions as a coactivator of the hypoxia‐inducible factor 1α (HIF‐1α)/E1Abinding protein p300 (p300) complex and increases casein kinase 2 regulatory subunit beta (CK2β) expression. Casein kinase 2 (CK2)dependent activation of protein kinase B (AKT) and inhibition of glycogen synthase kinase 3 beta (GSK3β) reduce the interaction of HIF‐1α with FBXW7γ, suppressing K48linked ubiquitination and proteasomal degradation of HIF‐1α. Stabilized HIF‐1α then reinforces the hypoxia responsive transcriptional program, creating a positive TET1/CK2/AKT/GSK3β/HIF‐1α feedback axis [137]. This example illustrates that TET1 can reshape ubiquitin‐dependent protein stability indirectly, without itself functioning as one of the E3 ligases or DUBs.

TET2 abundance is controlled by several degradation pathways. In hepatocellular carcinoma (HCC), the oncogenic fibroblast growth factor receptor 3 Δ7–9 splice variant (FGFR3Δ7–9) phosphorylates TET2 at Tyr1902 and promotes its ubiquitination and proteasomal degradation, reducing TET2dependent tumor‐suppressive activity and facilitating tumor progression [71]. Because the responsible ubiquitin ligase has not been fully defined, FGFR3Δ7–9 should be described as a kinase that primes TET2 for ubiquitination rather than as an E3 ligase. During neural stem cell differentiation, TET2 also interacts with components of the glucoseinduced degradation deficient/C terminal to LisH (GID/CTLH) E3 ligase complex. Depletion of the GID core components WDR26 or ARMC8 decreases TET2 ubiquitination and proteasomal degradation, increases global ^5−^hydroxymethylcytosine (^5^hmC), and promotes neuronal differentiation [72]. In another pathway, prolyl hydroxylase domain containing protein 2 and 3 (PHD2 and PHD3) hydroxylate conserved proline residues in TET2 and TET3, enabling recognition by the von Hippel–Lindau tumor suppressor protein (pVHL) containing ubiquitin ligase complex and subsequent polyubiquitination and proteasomal degradation [73]. These mechanisms couple TET2 abundance to oncogenic signaling, developmental differentiation, and oxygen sensing pathways.

TET2 is additionally regulated by functionally opposing DUBmediated mechanisms. CRL4^VprBP^‐mediated monoubiquitination of TET2 at Lys1299 promotes its chromatin association and catalytic activity [16]. USP15 removes this activating monoubiquitin modification and consequently reduces TET2 chromatin binding and enzymatic output [51]. In this setting, USP15 does not protect TET2 from degradation; instead, it suppresses TET2 function by reversing a nondegradative ubiquitin signal. This attenuation of TET2 activity alters chemokine expression and contributes to an immunosuppressive tumor microenvironment. By contrast, USP21 has been reported to directly interact with, deubiquitinate, and stabilize TET2 in airway epithelial cells exposed to cigarette smoke extract. Loss of USP21 increases TET2 degradation and exacerbates epithelial cell apoptosis, identifying a context‐specific USP21/TET2 protective axis [74]. These findings demonstrate that TET2 deubiquitination can either suppress catalytic activity or preserve protein abundance, depending on the ubiquitin architecture being removed.

During human immunodeficiency virus Type 1 (HIV‐1) infection, the viral protein Vpr hijacks the CRL4^VprBP^ E3 ligase complex to promote TET2 ubiquitination and proteasomal degradation, thereby sustaining interleukin‐6 (IL‐6) expression and facilitating viral replication [75].

TET2 also connects RNA cytosine oxidation to the regulation of chromatin deubiquitination. TET2 oxidizes ^5^‐methylcytosine (m^5^C) on chromatinassociated retrotransposon RNAs. In the absence of oxidation, RNA m^5^C is recognized by methyl CpGbinding domain protein 6 (MBD6), which associates with ASXL transcriptional regulator 1 (ASXL1) and recruits the BAP1 containing polycomb repressive deubiquitinase complex. This complex removes H2AK119Ub from nearby chromatin and promotes chromatin accessibility and transcriptional activation [138]. TET2mediated RNA m^5^C oxidation therefore antagonizes the MBD6/ASXL1/BAP1 axis and preserves H2AK119Ub at affected repetitive loci. Loss of TET2 increases RNA m^5^C recognition, enhances H2AK119Ub deubiquitination, produces a more open chromatin state, and activates transcriptional programs that support aberrant hematopoietic stem cell self‐renewal and leukemogenesis [138]. Thus, TET2 is both a substrate of deubiquitination and an upstream epigenetic regulator of a chromatinassociated DUB complex.

TET3 is regulated by both nondegradative monoubiquitination and degradation oriented polyubiquitination. Like TET1 and TET2, TET3 undergoes CRL4^VprBP^mediated monoubiquitination that promotes chromatin association. Conversely, PHD2/3dependent hydroxylation enables recognition of TET3 by pVHL and triggers ubiquitindependent proteasomal degradation [73]. The pathological importance of TET3 dosage is illustrated by human genetics and cancer studies. Loss of function and hypomorphic TET3 variants cause TET3related Beck/Fahrner syndrome, a neurodevelopmental disorder associated with impaired DNA demethylation [139]. In contrast, elevated TET3 can support AML by regulating glucose metabolic and leukemia stem cellassociated transcriptional programs [140]. These observations indicate that both inadequate and excessive TET3 activity can be pathogenic, depending on developmental and cellular context.

A recent preclinical study further demonstrated that pharmacological enhancement of TET3 degradation may be therapeutically exploitable. TET3 overexpressing pathogenic macrophages were identified in metabolic dysfunction associated with steatohepatitis, NSCLC, and endometriosis. In these cells, TET3 promotes inflammatory and immunosuppressive transcriptional programs involving NLR family pyrin domaincontaining 3 (NLRP3), IL‐1β, TGF‐β1, C–C motif chemokine ligand 2 (CCL2), and related mediators. Bobcat339 was reported to act as a TET3 selective molecular gluelike degrader by enhancing formation of a TET3/pVHL complex and accelerating pVHLdependent TET3 degradation. In macrophagebased experiments, Bobcat339 showed substantially greater activity toward TET3 than toward TET1 or TET2 and reproduced several effects of myeloidspecific TET3 deletion in mouse disease models [141]. Because these findings remain preclinical and cell contextdependent, Bobcat339 should be described as a reported molecular gluelike TET3 degrader rather than as an established clinical TET3 targeting drug.

Collectively, TET proteins illustrate several mechanistically distinct modes of ubiquitin epigenetic crosstalk. CRL4^VprBP^mediated monoubiquitination promotes chromatin binding and catalytic competence, whereas FGFR3, GID/CTLH, viral CRL4^VprBP^, and prolyl hydroxylase domain protein/pVHLassociated pathways regulate TET2 or TET3 abundance through proteasomal degradation. USP15 and USP21 produce opposing functional outcomes by reversing different ubiquitin signals on TET2. TET1 and TET2 can also regulate downstream ubiquitin or DUB systems through transcription or RNA oxidationdependent mechanisms, as exemplified by the TET1/RNF217/ferroportin and TET2/MBD6/BAP1 axes.

Overall, ubiquitination and deubiquitination regulate genome maintenance and DNA methylation through distinct but interconnected mechanisms. At DNA lesions, ubiquitin modifications establish signaling platforms that recruit and organize repair factors, whereas E3 ligases and DUBs control the stability, chromatin recruitment, and activity of DNMT and TET proteins. Disruption of these processes can impair DNA repair, alter methylation homeostasis, and contribute to genomic instability and diseaseassociated epigenetic dysregulation.

### 3.2 Ubiquitination and Deubiquitination of Epigenetic Enzymes in Inflammation and Immunity

Ubiquitin signaling regulates inflammation and immunity both through receptor proximal signaling proteins and by directly controlling epigenetic enzymes that shape the accessibility, magnitude, and duration of inflammatory gene programs. By modulating the abundance, activity, chromatin recruitment, and degradation fate of histone modifiers, DNA dioxygenases, and RNA modifying enzymes, E3 ligases and DUBs establish or terminate cell type‐specific inflammatory and immunoregulatory transcriptional states.

An instructive pathway links innate sensing in dendritic cells (DCs) to pathogenic T cell differentiation via lysine demethylase 4D (KDM4D) and H3K9Me2/3 demethylase. Upon Toll like receptor stimulation, the OTUdomain DUB TRABID/ZRANB1 deubiquitinates and stabilizes KDM4D. This enables KDM4D to remove repressive H3K9Me2/3 from the Il12a, Il12b, and Il23a promoters, facilitating REL proto‐oncogene, NFκB subunit (c‐Rel) recruitment and selective IL‐12 and IL‐23 induction. DCspecific Zranb1 deletion impaired Th1 and Th17 differentiation and protected mice from experimental autoimmune encephalomyelitis [142]. KDM4D is also regulated by selective autophagy: tripartite motif containing protein 14 (TRIM14) scaffolds USP14 and BRCC36/BRCC3 to remove K63linked ubiquitin chains from KDM4D, blocking optineurin (OPTN)dependent recognition and autophagic degradation. Consequently, TRIM14deficient DCs produce less IL‐12 and IL‐23 and fail to drive autoimmune inflammation [143]. Together, these studies reveal two complementary ubiquitin‐dependent mechanisms that sustain KDM4D levels, TRABIDmediated stabilization against proteasomal turnover, and TRIM14/USP14/BRCC3mediated protection from OPTNdependent autophagy.

Inflammatory resolution is controlled by USP38 through two coordinated chromatin activities. During lipopolysaccharide (LPS) stimulation, USP38 directly removes H2BK120 monoubiquitination at the Il6 and Il23a promoters, permitting recruitment of lysine demethylase 5B (KDM5B)/JARID1B. USP38 also removes K48linked polyubiquitin chains from KDM5B and protects it from proteasomal degradation. Stabilized KDM5B reduces H3K4Me3 and limits NFκB p65 subunit (p65), c‐Rel, and p50 binding, thereby limiting sustained IL‐6 and IL‐23p19 expression. Usp38deficient mice produced more of these cytokines and showed greater susceptibility to endotoxininduced acute lung injury, endotoxin shock, and dextran sulfate sodiuminduced acute colitis [144]. This H2BK120Ub/KDM5B/H3K4Me3 circuit is therefore a direct epigenetic brake on NFκBdependent inflammatory transcription rather than another example of ubiquitination of NFκB itself.

Conversely, degradation of an epigenetic repressor can amplify inflammation. In colonic CX3CR1^hi^ resident macrophages, inflammatory stimulation increases FBXW7 expression and promotes K48linked polyubiquitination and proteasomal degradation of enhancer of zeste homolog 2 (EZH2). Loss of EZH2 reduces histone H3 lysine 27 trimethylation (H3K27Me3) at the CCL2 and CCL7 promoters, thereby derepressing chemokine expression and promoting the recruitment of CX3CR1^int^ proinflammatory mononuclear phagocytes. Myeloid FBXW7 deletion or adenoassociated virus‐mediated FBXW7 knockdown reduced CCL2/CCL7, limited inflammatory cell accumulation, and alleviated dextran sulfate sodium or 2,4,6‐trinitrobenzene sulfonic acidinduced colitis [76]. This axis shows that ubiquitindependent loss of a histone methyltransferase can directly remodel a tissue immune niche.

Histone acetyltransferases provide a second class of ubiquitinregulated inflammatory effectors. CREBbinding protein (CBP) is targeted at Lys2103 by the S phase kinase‐associated protein 1 (SKP1)/Cullin 1 (CUL1)/F‐box and leucine‐rich repeat protein 19 (FBXL19) E3 ligase complex for K48linked ubiquitination and proteasomal degradation, whereas USP14 deubiquitinates and stabilizes CBP. LPS both reduces the CBP/FBXL19 interaction and FBXL19 abundance and enhances USP14dependent deubiquitination and stabilization of CBP, allowing CBP accumulation, histone H4 acetylation, and cytokine transcription. Genetic or pharmacological inhibition of USP14 reduced CBP abundance, histone acetylation, cytokine release, and systemic and pulmonary inflammation in mouse models of sepsis and acute lung injury [145]. Thus, opposing FBXL19 and USP14 activities directly couple CBP protein stability to acute inflammatory chromatin remodeling.

The related histone acetyltransferase p300 (encoded by EP300) is stabilized by distinct DUBs in chronic inflammatory microenvironments. In M2 like macrophages and lung cancer cells, USP24mediated p300 stabilization enhances histone H3 acetylation and supports an NFκB/IL‐6 transcriptional circuit that promotes tumor progression [146]. In alveolar Type II epithelial cells, ubiquitin C terminal hydrolase L3 (UCHL3) deubiquitinates p300 and facilitates p300 and C/EBPβdependent transcription of CCL2, CCL7, and CCL12, thereby promoting profibrotic macrophage polarization and pulmonary fibrosis [147]. These findings illustrate how DUBmediated stabilization of p300 can mediate communication between parenchymal cells and immune cells in chronic disease.

Linkagespecific ubiquitination of the same epigenetic enzyme can produce opposing immunological outcomes in distinct tumor contexts. In NSCLC, tripartite motif containing protein 35 (TRIM35) catalyzes nondegradative K63linked ubiquitination of LSD1/KDM1A at Lys422 and suppresses its catalytic activity. Reduced LSD1 activity upregulates endoplasmic reticulum (ER)/Golgi intermediate compartment protein 1, inhibits autophagic loss of interferon‐γ receptor 1 (IFNGR1), and strengthens interferon‐γ signaling, major histocompatibility complex Class I expression, cluster of differentiation 8 (CD8) ^+^ T cell infiltration, and tumor immune surveillance. TRIM35 low tumors were more responsive to combined LSD1 inhibition and antiprogrammed cell death protein 1 (PD‐1) therapy [148]. In breast cancer models, cyclindependent kinase (CDK) 9dependent phosphorylation instead promotes RNF20mediated K29linked polyubiquitination that stabilizes LSD1. Accumulated LSD1 lowers histone H3 lysine 4 (H3K4) methylation and represses endogenous retroviruses and interferon responses, whereas RNF20 loss increases sensitivity to anti‐PD‐1 therapy [149]. Together, these tumorspecific findings illustrate a contextdependent ubiquitin code in which K63linked modification suppresses LSD1 catalytic activity in NSCLC, whereas K29linked modification stabilizes LSD1 in breast cancer, resulting in opposing effects on antitumor immunity.

At the epitranscriptomic level, a recent study reported that LPS induces Olinked N‐acetylglucosamine (O‐GlcNAc) modification of the m^6^A demethylase fat mass and obesityassociated protein (FTO) at Ser95. This modification facilitates tripartite motif containing protein 21 (TRIM21)mediated K48linked ubiquitination and degradation of FTO. Reduced FTO increases m^6^A on suppressor of cytokine signaling 1 (Socs1) mRNA and sustains SOCS1 protein expression, thereby limiting IL‐1β, IL‐6, and TNF‐α production in macrophages. Reconstitution with the O‐GlcNAcdeficient FTO S95A mutant aggravated Salmonella Typhimurium or LPSinduced sepsis in mice [150]. Because this mechanism currently rests mainly on one recent cellular and mouse study, it should be regarded as an emerging inflammatory feedback pathway rather than an established general model.

USP15mediated removal of the activating CRL4^VprBP^dependent monoubiquitin mark limits TET2 chromatin engagement and promotes an immunosuppressive chemokine program, whereas pVHLdependent regulation of TET3 abundance influences inflammatory and immunosuppressive macrophage states [51, 141].

Ubiquitination and deubiquitination also control innate immune signaling beyond NFκB, including the cyclic GMP/AMP synthase (cGAS) stimulator of interferon genes (STING) axis. STING trafficking, oligomerization, and downstream interferon responses are tightly regulated by E3 ligases and DUBs, and this regulation shapes macrophage polarization and tumor immunity [151].

Collectively, ubiquitination and deubiquitination regulate inflammation and immunity through both the direct modulation of innate immune signaling components, such as STING, and the regulation of epigenetic and epitranscriptomic factors. Degradative and nondegradative ubiquitin signals, together with DUBmediated regulation, influence protein abundance, catalytic activity, chromatin recruitment, and inflammatory gene expression. These outcomes depend on ubiquitin architecture, substrate identity, cell lineage, and disease context.

### 3.3 Regulation of Cell Cycle Progression and Apoptosis

Ubiquitination and deubiquitination regulate cell cycle progression and apoptosis by controlling the stability and activity of key regulatory proteins. E3 ligases and DUBs modulate checkpoint proteins, transcription factors, and apoptotic regulators, including p53, MYC, p27, and myeloid cell leukemia 1 (MCL1), through ubiquitindependent protein turnover and nondegradative regulatory mechanisms. These mechanisms coordinate cell cycle transitions, cellular responses to stress, and the balance between cell survival and apoptosis.

The p53/MDM2 axis is one of the best characterized ubiquitination/deubiquitination modules linking cellular stress to cell cycle arrest and apoptosis. Under basal conditions, MDM2 binds p53 and promotes its ubiquitination, nuclear export, and proteasomal degradation, thereby maintaining low p53 protein levels [62]. Following DNA damage or oncogenic stress, disruption of MDM2dependent turnover allows p53 to accumulate and activate transcriptional programs governing cell cycle arrest, DNA repair, senescence, and apoptosis.

Several DUBs regulate this pathway. USP7 was initially identified as a p53associated DUB capable of deubiquitinating and stabilizing p53 [63]. However, USP7 also deubiquitinates and stabilizes MDM2 and MDMX, and its net effect on p53 signaling therefore depends on substrate abundance, cellular context, and stress conditions [152, 153]. USP10 directly deubiquitinates p53 and reverses MDM2‐induced p53 nuclear export and degradation, particularly after DNA damage [154]. USP11 similarly stabilizes p53 and supports p53 accumulation following genotoxic stress [155]. The final output of the p53 pathway is therefore determined by a dynamic balance between MDM2mediated ubiquitination and substrate selective deubiquitination.

MYC represents another proliferationdriving transcription factor controlled by competing E3 ligases and DUBs. SKP1 CUL1 F‐box protein complex (SCF) containing FBXW7 (SCF^FBXW7^) recognizes phosphorylationdependent degrons in MYC, including the Thr58 centered phosphodegron, and promotes MYC ubiquitination and degradation [156, 157]. Loss of FBXW7 function or disruption of the MYC degron stabilizes MYC and reinforces oncogenic transcription. SKP2 also promotes MYC ubiquitination but can simultaneously enhance MYCdependent transcriptional activity, illustrating that ubiquitination may remodel transcription factor function in addition to controlling turnover [158].

DUBs counteract MYC degradation. USP28 antagonizes FBXW7dependent MYC turnover and supports MYC stabilization in DNA damage and cancer contexts [159]. USP36, a nucleolar DUB, deubiquitinates and stabilizes MYC to sustain ribosome biogenesis and proliferationassociated transcription [160]. The relative activities of FBXW7, SKP2, USP28, and USP36 therefore help determine whether MYC is degraded, transcriptionally activated, or pathologically accumulated.

Cell cycle transitions are further controlled by the SCF and APC/C ubiquitin ligase systems. SCF complex containing SKP2 (SCF^SKP2^) promotes ubiquitination and degradation of p27, relieving inhibition of cyclin/CDK complexes and facilitating G1/S progression [161]. By contrast, the APC/C coordinates mitosis and mitotic exit. APC/C containing CDC20 (APC/C^CDC20^) targets securin and mitotic cyclins for degradation, permitting sister chromatid separation and anaphase onset, whereas APC/C^CDH1^ maintains low mitotic cyclin activity during late mitosis and G1 [162, 163]. These E3 ligases operate as temporally ordered ubiquitin switches that impose directionality on the cell cycle.

Apoptotic regulators are similarly controlled by ubiquitindependent turnover. MCL1 is a short lived antiapoptotic B cell lymphoma 2 (BCL‐2) family protein whose abundance strongly influences mitochondrial apoptosis. The HECT type E3 ligase HUWE1, also known as Mule or ARF/BP1, promotes MCL1 polyubiquitination and degradation, thereby lowering the apoptotic threshold under stress conditions [164]. Conversely, ubiquitinspecific peptidase 9, X linked (USP9X) removes K48linked ubiquitin chains from MCL1 and stabilizes it, promoting tumor cell survival and resistance to apoptosis [165]. The HUWE1/MCL1/USP9X axis forms a direct E3/DUB module that controls mitochondrial apoptosis competence.

Inhibitor of apoptosis proteins also use ubiquitindependent mechanisms to restrain cell death. Xlinked inhibitor of apoptosis protein (XIAP) contains a RING E3 ligase domain and can ubiquitinate Caspase‐3 and other apoptosis‐associated substrates, reinforcing suppression of executioner caspase activity [166]. Cellular inhibitor of apoptosis protein 1 (cIAP1) and cellular inhibitor of apoptosis protein 2 (cIAP2) regulate receptorinteracting serine/threonine protein kinase 1 (RIPK1) ubiquitination and receptor proximal signaling, favoring prosurvival NFκB activation while limiting the formation of apoptosis or necroptosis inducing complexes [167]. Thus, ubiquitination can promote apoptosis by eliminating survival proteins or suppress apoptosis by stabilizing prosurvival signaling complexes and limiting caspase activity.

Epigenetic regulators also intersect with proliferationassociated ubiquitin networks. EZH2 supports MYCdependent transcription and limits MYC and MYCN proto‐oncogene protein (MYCN) turnover, linking transcriptional regulation to protein stability [168, 169]. In addition, USP7mediated deubiquitination stabilizes EZH2 [18]. These findings connect ubiquitindependent regulation of EZH2 and MYC/MYCN protein stability with proliferation control.

Overall, ubiquitination and deubiquitination regulate cell cycle progression and apoptosis by controlling p53, MYC, CDK inhibitors, mitotic regulators, BCL‐2 family proteins, inhibitors of apoptosis proteins (IAPs), and epigenetic cofactors. K48linked ubiquitination commonly promotes proteasomal degradation of checkpoint or apoptotic regulators, whereas DUBs stabilize selected substrates and prolong their signaling functions. Nondegradative ubiquitination can additionally alter transcriptional activity or organize prosurvival signaling complexes. Disruption of these pathways promotes uncontrolled proliferation, apoptosis resistance, therapy resistance, and malignant transformation and provides a mechanistic foundation for therapeutic strategies targeting MDM2, selected DUBs, antiapoptotic proteins, and ubiquitindependent degradation systems.

### 3.4 Regulation of RNA Modification and Editing

The m^6^A machinery comprises methyltransferase writers, demethylase erasers, and RNAbinding readers that regulate RNA processing, stability, and translation [170, 171]. Ubiquitination and deubiquitination, together with other posttranslational modifications, modulate the stability, complex assembly, and activity of these regulators, thereby influencing m^6^A deposition, removal, and recognition.

The m^6^A regulatory network is not limited to cancer; it also plays important roles in skeletal muscle development, myoblast proliferation and differentiation, and muscle regeneration, with potential implications for muscle‐related diseases [172]. Given that METTL3, FTO, ALKBH5, and YTH domain family (YTHDF) proteins are subject to ubiquitindependent turnover, their regulation by ubiquitination and deubiquitination may also be relevant to skeletal muscle homeostasis and disease.

#### 3.4.1 METTL3/METTL14/Wilms tumor 1 associating protein (WTAP) Complex Writer Axis

The m^6^A writer complex comprises a METTL3/METTL14 heterodimer, in which METTL3 provides catalytic activity and METTL14 supports RNA substrate recognition, together with regulatory components such as WTAP [170]. Its abundance and assembly are controlled by kinase signaling, E3 ligases, and DUBs.

The stability of the m^6^A writer complex is regulated through crosstalk between kinases and DUBs. ERK phosphorylates METTL3 at Ser43, Ser50, Ser525 and WTAP at Ser306 and Ser341. These phosphorylation events promote their interaction with USP5, which removes ubiquitin modifications and stabilizes the METTL3/METTL14/WTAP writer complex, thereby increasing global m^6^A deposition. This pathway directly links extracellular kinase signaling to DUBmediated control of epitranscriptomic activity [19].

METTL3 is also targeted by degradationoriented ubiquitination. In pancreatic ductal adenocarcinoma (PDAC), TRIM21 promotes K48linked polyubiquitination and proteasomal degradation of METTL3, reducing METTL3dependent oncogenic signaling and increasing ferroptosis and responsiveness to anti‐PD‐1 treatment [88]. In alcoholassociated steatohepatitis, STUB1dependent METTL3 ubiquitination and degradation disrupt hepatic m^6^A homeostasis and contribute to inflammatory and metabolic injury [89]. These studies indicate that E3 ligasemediated reduction of METTL3 abundance can generate distinct biological outcomes depending on the context of the disease.

METTL3 stability is further regulated by crosstalk between O‐GlcNAcylation and ubiquitination. In HCC, METTL3 is O‐GlcNAcylated at Thr186, Ser192, and Ser193. This modification weakens its interaction with FBXW7, reduces ubiquitination, and increases its protein halflife. O‐GlcNAcylation also enhances METTL3 binding to WTAP, thereby supporting writer complex assembly and maintaining m^6^A levels [90]. Thus, METTL3 abundance is controlled not only by direct E3 ligase and DUB activities but also by competing modifications that alter their accessibility to the ubiquitin machinery.

METTL14 is regulated through related but distinct degradation pathways. STUB1 directly interacts with METTL14 and promotes its ubiquitination at Lys148, Lys156, and Lys162, resulting in proteasomal degradation and reduced cellular m^6^A levels. METTL3 protects METTL14 from STUB1mediated turnover by competing for METTL14 binding, indicating that assembly of the writer complex itself contributes to METTL14 stability [91].

In the liver, HMGCoA reductase degradation protein 1 (HRD1)mediated ERassociated degradation (ERAD) regulates METTL14 protein stability. During ER stress, unfolded protein accumulation suppresses HRD1mediated METTL14 ubiquitination and degradation, allowing METTL14 to accumulate. Increased METTL14 promotes m^6^Adependent decay of C/EBP homologous protein (CHOP) mRNA, thereby limiting CHOPmediated apoptosis and protecting against ER proteotoxic liver injury [92]. This mechanism links ER protein quality control to adaptive regulation of the RNA methylome.

WTAP is also independently regulated by ubiquitination‐related mechanisms. In glioblastoma (GBM), O‐GlcNAc transferase (OGT)mediated O‐GlcNAcylation increases WTAP stability and supports oncogenic m^6^A programs; the study further links WTAP stability to USP7dependent deubiquitination [173]. Conversely, activation of type I interferon signaling promotes K48‐linked ubiquitination and proteasomal degradation of WTAP. WTAP loss reduces m^6^A modification of IRF3 and interferon alpha and beta receptor subunit 1 (IFNAR1) transcripts, thereby suppressing IRF3 translation and destabilizing IFNAR1 mRNA as part of a feedback mechanism limiting antiviral signaling [93].

Collectively, the abundance and activity of the writer machinery are determined by a balance among E3 ligases, DUBs, writer complex assembly, phosphorylation, and O‐GlcNAcylation. These regulatory layers can change global or transcript selective m^6^A deposition without altering the expression of the corresponding writer genes.

#### 3.4.2 FTO

FTO is an RNA demethylase whose abundance is controlled by DUBs and by degradation through the ubiquitinproteasome system (UPS) or the autophagylysosome pathway (ALP). It therefore illustrates how ubiquitindependent protein homeostasis can regulate RNA modification through distinct degradation routes.

Direct deubiquitination provides one mechanism for maintaining FTO stability. In gemcitabine resistant PDAC cells, USP7 interacts with FTO and stabilizes it at the protein level without altering FTO mRNA abundance. USP7 depletion increases FTO ubiquitination and accelerates its degradation, whereas WT USP7, but not a catalytically inactive mutant, reduces FTO ubiquitination and prolongs its protein halflife. Mechanistically, USP7 preferentially removes K48linked polyubiquitin chains from FTO, whereas K63linked ubiquitination is largely unaffected. The USP7/FTO axis consequently contributes to FTO accumulation and gemcitabine resistance in PDAC [94].

An opposing degradation pathway involves the deltex E3 ubiquitin ligase 2 (DTX2). DTX2 ubiquitinates FTO, followed by recruitment of ubiquitin recognition factor in ERassociated degradation 1 (UFD1) and proteasomal elimination of FTO. Vitamin E succinate (VES) enhances the interaction between FTO and DTX2, thereby increasing FTO ubiquitination and degradation. Genetic or VESinduced FTO depletion enhances tumor intrinsic immune signaling, T cell cytotoxicity, and responsiveness to immune checkpoint therapy. These findings establish the DTX2/UFD1 axis as a pharmacologically exploitable UPSdependent pathway regulating FTO abundance [95].

FTO degradation can also be routed between the UPS and ALP in response to hypoxia. Under hypoxic conditions, FTO is translocated from the nucleus to the cytoplasm, where its interaction with the E3 ligase neural precursor cell expressed, developmentally downregulated 4 (NEDD4) is enhanced. Hypoxia induces both K48 and K63linked polyubiquitination of FTO. K48linked chains are primarily associated with proteasomal degradation, whereas K63linked chains promote p62/SQSTM1dependent delivery of FTO to the ALP. Depletion of ATG7 reduces both K48 and K63linked FTO ubiquitination and impairs FTO degradation through both routes [96]. However, ATG7 is an autophagyrelated E1 like enzyme that activates ubiquitin like conjugation systems, not canonical ubiquitinactivating E1. This pathway links hypoxiainduced subcellular redistribution, ubiquitin chain architecture, and FTO's degradative fate.

A related p62dependent mechanism operates during chronic low level arsenic exposure. Under basal conditions, FTO interacts with p62/SQSTM1 through the UBA domain of p62 and localizes to LC3positive autophagic structures. Genetic disruption of p62, ATG5, or ATG7 increases FTO stability, whereas p62 restoration promotes FTO degradation. Chronic low level arsenic exposure suppresses p62 expression and impairs selective autophagic degradation of FTO, resulting in FTO accumulation, reduced RNA m^6^A levels, and malignant transformation. FTO accumulation can in turn inhibit autophagy, generating a positive feedback loop that sustains FTO elevation [97]. Although this study demonstrated that ubiquitination and the UBA domain of p62 contribute to FTO recognition, the precise ubiquitin chain topology responsible for FTO routing was not fully resolved.

Collectively, FTO stability is regulated by opposing ubiquitindependent mechanisms. USP7 removes K48linked ubiquitin chains and stabilizes FTO, whereas DTX2/UFD1 and hypoxia responsive NEDD4associated pathways promote its degradation. Depending on ubiquitin chain architecture and cellular context, ubiquitinated FTO can be routed to either the UPS or p62dependent ALP. These direct protein homeostasis mechanisms should be distinguished from downstream pathways in which FTO regulates autophagyrelated transcripts through its m^6^A demethylase activity.

#### 3.4.3 ALKBH5

ALKBH5 is an m^6^A RNA demethylase whose abundance is controlled by opposing E3 ligases and DUBs, linking protein turnover to RNA methylation and cellular responses.

In GBM, USP36 directly interacts with ALKBH5, reduces its ubiquitination, and stabilizes the demethylase. USP36 depletion shortens the ALKBH5 protein halflife, suppresses the proliferation and self‐renewal of GBM stem cells (GSCs), reduces intracranial tumor growth, and sensitizes GSCs to temozolomide (TMZ). The USP36/ALKBH5 axis therefore promotes GSC maintenance and GBM progression by preventing ubiquitindependent ALKBH5 degradation [98].

An opposing regulatory mechanism involves bromodomain containing protein 4 (BRD4) and the E3 ligase TRIM21 in breast cancer. BRD4 functions as a molecular scaffold that facilitates the association of TRIM21 with ALKBH5, thereby promoting ALKBH5 ubiquitination and degradation. Genetic or pharmacological inhibition of BRD4 reduces ALKBH5 ubiquitination and increases its protein abundance. The resulting changes in ALKBH5 activity regulate m^6^A modification of extra spindle pole bodies like 1 mRNA and influence breast cancer progression [99].

ALKBH5 stability is also regulated through phosphorylationdependent recruitment of a DUB. In GSCs, HECT, C2, and WW domain containing E3 ubiquitin protein ligase 2 (HECW2) promotes K48linked ubiquitination and proteasomal degradation of ALKBH5, with lysine 295 (K295) serving as an important ubiquitination site. Following ionizing radiation, mammalian sterile 20 like kinase 4 (MST4) phosphorylates ALKBH5 at serine 64 and serine 69 (S64/S69). This phosphorylation enhances the interaction of ALKBH5 with USP14, promotes its deubiquitination, and counteracts HECW2mediated degradation. The MST4/USP14/ALKBH5 axis consequently supports GSC stem like properties, homologous recombination‐mediated DNA DSB repair, tumorigenicity, and resistance to radiotherapy [100].

A further degradationoriented pathway has been described in ulcerative colitis (UC). In dextran sulfate sodiuminduced UC mouse models, RNF180 is increased, whereas ALKBH5 abundance is reduced. RNF180mediated ubiquitination decreases ALKBH5 expression, thereby relieving ALKBH5dependent repression of sucrose nonfermenting 2 (SNF2)related chromatin remodeling ATPase 5 (SMARCA5) and exacerbating colonic inflammation and the imbalance between T helper 17/regulatory T cells (Th17/Treg). RNF180 depletion or ALKBH5 restoration ameliorates inflammatory injury and restores Th17/Treg balance, supporting the functional importance of the RNF180/ALKBH5/SMARCA5 axis in UC [101].

Collectively, ALKBH5 abundance is determined by opposing ubiquitination and deubiquitination pathways. USP36 and the MST4/USP14 module stabilize ALKBH5, whereas TRIM21, HECW2, and RNF180 promote their ubiquitination or reduce its abundance. These mechanisms connect oncogenic signaling, the DDR, and inflammatory cues to the stability and functional output of an RNA m^6^A demethylase.

#### 3.4.4 YTH m^6^A RNABinding Protein 1, YTH m^6^A RNA‐Binding Protein 2, and YTH m^6^A RNABinding Protein 3

YT521‐B homology (YTH) m^6^A RNAbinding protein 1 (YTHDF1), YTH m^6^A RNAbinding protein 2 (YTHDF2), and YTH m^6^A RNAbinding protein 3 (YTHDF3) constitute the principal cytoplasmic branch of the YTH domain m^6^A reader family. By recognizing m^6^Amodified transcripts, these proteins regulate translation, RNA decay, stress adaptation, and disease‐associated gene expression programs. Their biological output is determined not only by RNA‐binding specificity but also by posttranslational mechanisms that control protein abundance and activity. Direct ubiquitindependent regulation is well established for YTHDF1 and YTHDF2, whereas the E3 ligases and deubiquitinases responsible for basal YTHDF3 turnover remain less completely defined.

YTHDF1 stability is coordinately regulated by the opposing activities of DUBs and E3 ligases. USP5 directly interacts with YTHDF1 and removes K11linked polyubiquitin chains, thereby prolonging its protein halflife and preventing proteasomal degradation [174]. Insulin/mechanistic target of rapamycin complex 1 (mTORC1) signaling promotes USP5 phosphorylation and dimerization, strengthening the USP5/YTHDF1 interaction. Conversely, the Cullin 7 FBXW8 E3 ligase complex (CUL7^FBXW8^) binds YTHDF1, catalyzes K11linked polyubiquitination, and directs it toward proteasomal degradation. This USP5/YTHDF1/CUL7^FBXW8^ module regulates tumor intrinsic immune transcriptional programs and modulates responsiveness to programmed death ligand 1 (PD‐L1) directed immunotherapy. Thus, K11linked ubiquitin chains, which are frequently associated with cell cycle regulation and proteolysis, can also control the abundance of an m^6^A reader and its immunological output.

In addition to being a ubiquitinregulated substrate, YTHDF1 can reshape downstream ubiquitindependent protein turnover through its RNAreading activity. In prostate cancer, YTHDF1 recognizes m^6^Amodified RNF7 transcripts and enhances their translation. Increased RNF7, a RING component of Cullin/RING E3 ligase machinery, promotes proteasomal degradation of the CDK inhibitor p27, thereby facilitating cell cycle progression, tumor cell proliferation, migration, and xenograft growth [175]. Depletion of YTHDF1 or RNF7 increases p27 abundance and enhances cisplatininduced apoptosis. The YTHDF1/RNF7/p27 pathway therefore demonstrates bidirectional integration with the ubiquitin system: YTHDF1 abundance is controlled by ubiquitination and deubiquitination, whereas YTHDF1 can also regulate translation of an E3 ligase component and indirectly determine the stability of another protein.

YTHDF2 is subject to several contextdependent ubiquitinproteasome pathways. During cell cycle progression, CUL1, SKP2, Cullin 4A (CUL4A), and DDB1associated ubiquitin ligase machinery promotes YTHDF2 proteolysis, whereas CDK1 activity preserves YTHDF2 protein stability [176]. Stabilized YTHDF2 recognizes m^6^Amodified WEE1 like protein kinase (WEE1) transcripts and accelerates their decay, reducing WEE1mediated inhibitory phosphorylation of CDK1 and promoting G_2_/M transition. CDK1, YTHDF2, and WEE1 consequently form a feed forward circuit that coordinates transcript decay with mitotic entry. Because CUL1/SKP2 and CUL4A/DDB1 represent distinct cullinassociated ligase systems, these proteins should be presented as identified components of YTHDF2 regulatory E3 machinery rather than as a single four component E3 ligase complex.

Diseaseassociated E3 ligases also regulate YTHDF2 stability with contextspecific consequences. In ovarian cancer, FBXW7mediated degradation of YTHDF2 limits the decay of proapoptotic Bcl‐2 modifying factor (BMF) mRNA, enhancing apoptosis and suppressing tumor growth [177]. In prostate cancer, F‐box protein 2 (FBXO2) promotes YTHDF2 ubiquitination and proteasomal degradation, thereby preserving the tumor suppressive CDK inhibitor 1C (CDKN1C) transcript and restraining malignant progression [178]. These mechanisms illustrate how distinct E3 ligases converge on the same m^6^A reader while regulating different downstream transcripts.

OTU deubiquitinase, ubiquitin aldehyde‐binding 1 (OTUB1) produces the opposite effect by protecting YTHDF2 from ubiquitination. OTUB1 binds YTHDF2 and inhibits ubiquitin transfer to the reader independently of its conventional catalytic deubiquitinase activity, thereby stabilizing YTHDF2 [53]. Stabilized YTHDF2 recognizes m^6^Amodified serine protease 8 (PRSS8) mRNA and promotes its decay. Reduced PRSS8 expression weakens E‐cadherinassociated restriction of nuclear β‐catenin and supports prostate cancer cell proliferation. The OTUB1/YTHDF2 pathway therefore represents noncanonical antiubiquitination rather than direct cleavage of pre‐existing ubiquitin chains from YTHDF2 [53]. Together with the FBXO2 pathway, it demonstrates that opposing E3 and OTUB1dependent mechanisms can generate divergent biological outputs by controlling the abundance of the same m^6^A reader.

Competing posttranslational modifications can further determine YTHDF2 stability. During hepatitis B virus (HBV) infection, increased hexosamine biosynthetic pathway activity promotes OGT‐mediated O‐GlcNAcylation of YTHDF2 at Ser263. This modification suppresses YTHDF2 ubiquitination, prolongs its protein halflife, and protects it from proteasomal degradation [179]. Stabilized YTHDF2 increases the abundance of m^6^Amodified MCM2 and MCM5 transcripts and promotes cell cycle progression and HBVassociated HCC development. This mechanism illustrates how O‐GlcNAcylation can compete with degradationoriented ubiquitination and translate nutrient and viral signals into altered m^6^A reader abundance.

Compared with YTHDF1 and YTHDF2, the direct ubiquitindependent regulation of YTHDF3 remains less completely characterized. Recent evidence indicates that lactate accumulation promotes alanyl transfer RNA (tRNA) synthetase 2, mitochondrial (AARS2)dependent lactylation of YTHDF3 in bladder cancer. This modification reduces ubiquitinassociated turnover and stabilizes YTHDF3, although the E3 ligase responsible for degradation of the unmodified protein has not yet been identified [180]. Stabilized YTHDF3 promotes m^6^Adependent decay of lysine demethylase 6B (KDM6B) RNA, reduces KDM6B abundance, increases H3K27Me3dependent repression of CDKN1A, and contributes to cisplatin resistance. The lactate AARS2/YTHDF3/KDM6B/CDKN1A pathway therefore connects metabolic reprogramming with protein lactylation, ubiquitinrelated proteostasis, RNA methylation, and chromatin regulation.

These findings show that YTHDF protein abundance is controlled by E3 ligasedependent proteasomal turnover, catalytic deubiquitination, noncatalytic inhibition of ubiquitination, and crosstalk with other posttranslational modifications. Conversely, readerdependent translation of an E3 ligase component can alter downstream substrate turnover, linking m^6^A recognition to protein stability.

#### 3.4.5 YTH DomainContaining Protein 1 and YTH DomainContaining Protein 2

YTH domaincontaining protein 1 (YTHDC1) and YTH domaincontaining protein 2 (YTHDC2) constitute the nuclear and helicase containing branches of the YTH domain m^6^A reader family, respectively. YTHDC1 is predominantly localized in the nucleus and regulates m^6^Adependent pre‐mRNA splicing, nuclear export, transcript stability, and chromatinassociated RNA processing. YTHDC2 is primarily cytoplasmic and combines an m^6^A recognition domain with RNA helicase activity, supporting RNA metabolism, meiotic progression, and germ cell development. Emerging evidence indicates that YTHDC1 is subject to multiple ubiquitin‐dependent regulatory mechanisms, whereas the direct ubiquitindependent control of YTHDC2 remains insufficiently defined.

YTHDC1 can be directly targeted for ubiquitindependent proteasomal degradation. In bladder cancer, the RING containing E3 ligase XIAP binds YTHDC1, promotes its ubiquitination, shortens its protein halflife, and directs it toward proteasomedependent degradation [181]. Loss of YTHDC1 slows the decay of m^6^Amodified matrix metallopeptidase 2 (MMP2) mRNA, increases MMP2 expression, and promotes bladder cancer cell invasion and pulmonary metastasis. Mutations or deletion of the XIAP RING domain impair YTHDC1 ubiquitination and degradation, supporting YTHDC1 as a direct XIAP substrate. The XIAP/YTHDC1/MMP2 axis therefore provides direct evidence that the abundance of a nuclear m^6^A reader can be controlled by an E3 ligase in a disease relevant manner.

A second degradation pathway involves RNF183. Under high glucose conditions, increased glucose transporter 3 (GLUT3) expression enhances RNF183 abundance in bladder cancer cells. RNF183 directly interacts with YTHDC1 and catalyzes its K48linked polyubiquitination, resulting in proteasomedependent degradation [182]. RNF183 depletion prolongs the YTHDC1 halflife and decreases its polyubiquitination, whereas WT RNF183, but not a ligase inactive mutant, accelerates YTHDC1 turnover. Because YTHDC1 destabilizes SLC2A3/GLUT3 mRNA, the GLUT3/RNF183/YTHDC1 pathway forms a positive feedback circuit that reinforces glycolytic reprogramming and malignant progression in bladder cancer.

Metabolic posttranslational modifications further influence this RNF183dependent pathway. In hyperglycemic bladder cancer models, increased lactate production promotes AARS1mediated lactylation of YTHDC1, which enhances RNF183mediated YTHDC1 ubiquitination and degradation [183]. Reduced YTHDC1 decreases the stability of m^6^Amodified JUND mRNA, lowers nectin cell adhesion molecule 4 (NECTIN4) expression, and diminishes responsiveness to the NECTIN4 targeting antibody/drug conjugate enfortumab vedotin. This mechanism illustrates how lactylation can facilitate E3 ligase recognition and convert a metabolic signal into ubiquitindependent loss of an RNA modification reader.

YTHDC1 stability can also be preserved by proteins that antagonize its ubiquitination. In MYCN amplified neuroblastoma, KRABassociated protein 1 (KAP1), also termed TRIM28, directly interacts with YTHDC1 and protects it from ubiquitinproteasomedependent degradation [184]. KAP1 depletion increases YTHDC1 ubiquitination, accelerates its turnover, and reduces the stability of m^6^Amodified MYCN mRNA. Conversely, KAP1 forms a complex with YTHDC1 and METTL3, stabilizing YTHDC1 and sustaining MYCN expression and neuroblastoma growth. The E3 ligase responsible for basal YTHDC1 ubiquitination in this setting was not identified; KAP1 should therefore be described as a protective interaction partner rather than as a YTHDC1specific deubiquitinase.

In addition to being a ubiquitin substrate, YTHDC1 can indirectly reshape downstream ubiquitindependent protein stability. In colorectal cancer (CRC) cells, YTHDC1 suppresses expression of the HECT family E3 ligase UBE3A. Loss of YTHDC1 increases UBE3A expression, enhances RAD51 recombinase (RAD51) ubiquitination and degradation, and promotes apoptosis [185]. This pathway suggests that YTHDC1 participates bidirectionally in the ubiquitin network: its own abundance is controlled by E3 ligases, while its RNA regulatory activity can alter the expression of an E3 ligase and thereby influence turnover of a DNA repair protein. Nevertheless, a direct DUB that specifically deubiquitinates and stabilizes YTHDC1 has not yet been firmly established.

Collectively, these findings highlight a bidirectional relationship between YTHDC1mediated RNA regulation and ubiquitindependent protein turnover. Control of YTHDC1 abundance alters RNA fate, while YTHDC1 can influence downstream protein stability by regulating E3 ligase expression. The biological outcome varies with the tumor context, as illustrated by its tumor suppressive effects in the bladder cancer models described here and its support of MYCN expression and neuroblastoma growth.

#### 3.4.6 N‐Acetyltransferase 10

N‐acetyltransferase 10 (NAT10) is a multifunctional acetyltransferase with both RNA and protein acetyltransferase activities. As the principal writer of N^4^‐acetylcytidine (ac^4^C), NAT10 modifies multiple RNA species and influences RNA stability, translation, ribosome biogenesis, and cell state regulation. NAT10 also participates in bidirectional crosstalk with the ubiquitin system: its own abundance is controlled by ubiquitination and deubiquitination, whereas NAT10dependent acetylation can either inhibit or facilitate the ubiquitination of downstream proteins.

NAT10 protein stability is directly regulated by opposing ubiquitindependent mechanisms. In head and neck squamous cell carcinoma (SCC), RNAbinding protein with serinerich domain 1 (RNPS1) binds NAT10 and interferes with zinc finger SWIM type containing 6 (ZSWIM6)mediated ubiquitination and proteasomal degradation. Stabilized NAT10 maintains tRNA ac^4^C modification and supports translation programs associated with tumor cell proliferation and survival [102]. This mechanism identifies ZSWIM6 as a degradationoriented E3 ligase for NAT10 and RNPS1 as a protective binding partner that limits E3 ligasedependent substrate turnover.

A deubiquitinationdependent mechanism has also been reported in activated macrophages. LPS stimulation increases the interaction between NAT10 and USP39, and USP39 removes K48linked polyubiquitin chains from NAT10, thereby suppressing its proteasomal degradation and prolonging its halflife [103]. NAT10 Lys195 and Lys426 were implicated as important ubiquitination sites in this pathway. Stabilized NAT10 subsequently increases ac^4^C modification of ETS2 mRNA, enhances its stability and translation, and promotes proinflammatory macrophage activation and inflammationassociated cardiac dysfunction [103]. Notably, this study found that NAT10 turnover in activated macrophages was proteasomedependent rather than autophagy or lysosome dependent.

NAT10 can, in turn, control the ubiquitin‐dependent stability of other proteins through substrate acetylation. In activated T cells, NAT10 acetylates receptor for activated C kinase 1 (RACK1) at Lys185, preventing its K48linked polyubiquitination and proteasomal degradation. Stabilized RACK1 supports ribosome assembly, metabolic reprogramming, and T cell proliferation, thereby contributing to cycling T cellmediated autoimmune and inflammatory phenotypes [186]. This pathway demonstrates that acetylation can antagonize degradationoriented ubiquitination and stabilize a functionally important signaling and ribosomal scaffold.

Acetylation can also produce the opposite effect by facilitating substrate ubiquitination. In vascular smooth muscle cells exposed to a diabetic environment, NAT10 acetylates Parkinson disease protein 7 (DJ‐1) at Lys93, increasing the interaction between DJ‐1 and the E3 ligase tripartite motif containing protein 32 (TRIM32). TRIM32 subsequently ubiquitinates DJ‐1 primarily at Lys41 and promotes its proteasomal degradation [187]. Loss of the protective DJ‐1 protein activates STAT1dependent RAGE transcription and promotes vascular smooth muscle cell osteogenic conversion and plaque microcalcification. Thus, NAT10dependent acetylation may either block ubiquitination, as observed for RACK1, or create a molecular context that facilitates E3 ligase recognition, as observed for DJ‐1.

NAT10 has additionally been linked to the ubiquitindependent stability of NFκB/p65. During cutaneous wound repair, NAT10 maintains nuclear p65 abundance by restraining its polyubiquitination and nuclear degradation, thereby sustaining IL‐6/IL‐8/STAT3 signaling and keratinocyte migration [188]. However, the responsible E3 ligase and whether NAT10 directly acetylates p65 were not established in that study. This pathway should therefore be described as NAT10dependent suppression of p65 ubiquitination rather than as a fully resolved acetylation ubiquitination switch.

Collectively, NAT10 illustrates several modes of acetylationubiquitination crosstalk. ZSWIM6 promotes NAT10 degradation, whereas RNPS1 and USP39 stabilize NAT10 by limiting its ubiquitination or removing K48‐linked chains. NAT10 subsequently regulates downstream proteins in a substrate‐dependent manner: acetylation protects RACK1 from degradation driven by K48‐linked ubiquitination but facilitates TRIM32‐dependent ubiquitination and degradation of DJ‐1. These mechanisms connect ac^4^C regulation and protein acetylation with proteasomal protein turnover, inflammatory signaling, translation, and disease progression. Current evidence does not establish ubiquitin‐dependent selective autophagy as a direct mechanism controlling NAT10 or its substrates in these pathways.

#### 3.4.7 Adenosine Deaminase Acting on RNA 1

Adenosine deaminase acting on RNA 1 (ADAR1) catalyzes adenosine‐to‐inosine (A‐to‐I) editing within double stranded RNA and thereby regulates RNA structure, transcript processing, translation, and discrimination between endogenous and immunogenic RNA. ADAR1 is expressed mainly as the constitutive nuclear p110 isoform and the interferon inducible p150 isoform. Emerging evidence indicates that ADAR1 abundance and editing activity are regulated by noncanonical ubiquitin signals that may either protect the enzyme from degradation or promote its removal.

The HECT family E3 ligase SMADspecific E3 ubiquitin protein ligase 2 (SMURF2) directly binds and oligoubiquitinates the 110 kDa ADAR1 isoform (ADAR1p110) at Lys744. Unlike canonical degradationoriented ubiquitination, this modification stabilizes ADAR1p110, protects it from both proteasomal and lysosomal turnover, and enhances its A‐to‐I RNA editing activity [104]. Mutation of Lys744 to arginine reduces ADAR1p110 stability, increases its susceptibility to proteolysis through both pathways, and impairs editing activity. The SMURF2/ADAR1p110 axis therefore provides a direct example in which ubiquitination prevents, rather than initiates, protein clearance and simultaneously influences proteasomal and lysosomal degradation routes. The study did not identify a specific lysosomal cargo receptor, so the lysosomal branch should not be described as a fully defined ubiquitindependent selective autophagy mechanism.

ADAR1 can also be regulated by a degradation oriented E3 ligase pathway. In human epidermal growth factor receptor 2 (HER2)positive breast cancer, the long noncoding RNA long intergenic nonprotein coding RNA 624 (LINC00624) binds ADAR1 and inhibits its beta transducin repeat containing protein (β‐TrCP)mediated ubiquitination and degradation, thereby increasing ADAR1 abundance [105]. Stabilized ADAR1 supports immunosuppressive transcriptional and RNA editing programs, reduces antitumor immune activity, and contributes to resistance to HER2targeted therapy. This mechanism complements the SMURF2 pathway: SMURF2 directly installs a stabilizing ubiquitin modification on ADAR1p110, whereas LINC00624 stabilizes ADAR1 by interfering with ubiquitination initiated by the degradationoriented SCF complex containing β‐TrCP (SCF^β‐TrCP^) machinery.

Collectively, ADAR1 demonstrates that ubiquitination is not intrinsically degradative. SMURF2mediated oligoubiquitination at Lys744 protects ADAR1p110 from proteasomal and lysosomal degradation and enhances editing activity, whereas β‐TrCPassociated ubiquitination promotes ADAR1 turnover and is antagonized by LINC00624. The fate of ADAR1 therefore depends on the responsible E3 ligase, the architecture and position of the ubiquitin modification, competing molecular interactions, and the degradation pathway available in each cellular context (Figure 2).

**FIGURE 2:**
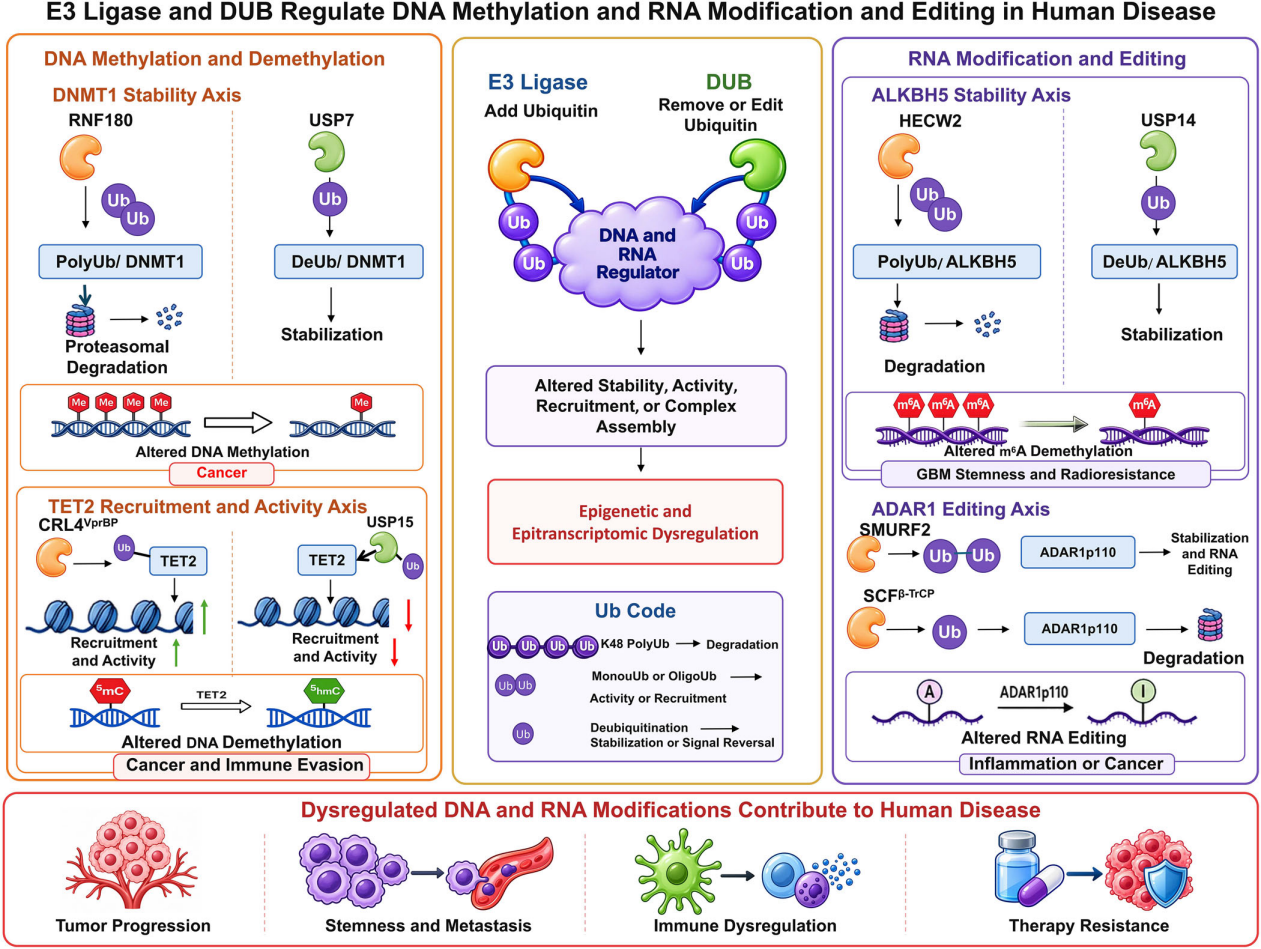
E3 ligases and DUBs regulate DNA methylation and RNA modification in human disease. This schematic summarizes how E3 ligases and DUBs regulate DNA and RNA modification regulators. RNF180 promotes DNMT1 degradation via ubiquitination; USP7 stabilizes DNMT1 by deubiquitination. CRL4^VprBP^mediated monoubiquitination controls TET chromatin binding, while other ubiquitination events drive TET degradation. E3 ligases and DUBs also target m^6^A regulators, including METTL3/14/WTAP, FTO, ALKBH5, and YTHDF1, impacting RNA metabolism, tumor progression, immune regulation, and therapy resistance.

### 3.5 UbiquitinDependent Regulation of Histone Modification and Chromatin Remodeling

#### 3.5.1 Histone Modifying Enzymes: UbiquitinDependent Turnover, Deubiquitination, and Selective Autophagy Crosstalk

Histone modifying enzymes provide prominent examples of how ubiquitination and deubiquitination regulate epigenetic states through mechanistically distinct routes. Ubiquitindependent proteolysis controls the abundance of histone methyltransferases, demethylases, and deacetylases, whereas nondegradative ubiquitination and deubiquitination can alter their chromatin recruitment, interaction networks, substrate recognition, or catalytic output. Certain histone modifying enzymes additionally recognize ubiquitinated cargo or regulate its routing between the proteasome and autophagy lysosome system. These direct mechanisms should be distinguished from transcriptional regulation of ATGs, which does not by itself establish ubiquitindependent autophagic degradation.

##### 3.5.1.1 EZH2

EZH2 is the catalytic subunit of PRC2 and catalyzes H3K27 methylation to establish transcriptionally repressive chromatin. In addition to its canonical PRC2dependent activity, EZH2 has noncanonical oncogenic functions that cannot necessarily be suppressed by catalytic inhibition. In mixed lineage leukemia (MLL) rearranged acute leukemia, EZH2 occupies nonPRC2 target genes lacking H3K27Me3 and associates with MYC and the transcriptional coactivator p300 through a cryptic transactivation domain (TAD) within EZH2. The resulting EZH2/MYC/p300 complex promotes transcriptional activation and leukemic growth independently of canonical PRC2‐mediated repression [168]. This finding provides a rationale for eliminating the entire EZH2 protein, which may suppress both catalytic and noncatalytic functions, rather than inhibiting only its methyltransferase activity.

EZH2 abundance is controlled by opposing ubiquitination and deubiquitination pathways. USP7 physically associates with PRC2, directly deubiquitinates and stabilizes EZH2, and promotes PRC2 chromatin loading and transcriptional repression. USP7 depletion consequently reduces EZH2 abundance and weakens PRC2dependent gene silencing, demonstrating that DUB activity can regulate both protein stability and chromatin occupancy [189].

An opposing pathway operates in melanoma. UHRF1dependent methylation of the ubiquitinconjugating enzyme E2 L6 (UBE2L6) promoter suppresses UBE2L6 expression and indirectly stabilizes EZH2. When expressed, the E2 enzyme UBE2L6 cooperates with the E3 ligase ubiquitin protein ligase E3 component N recognin 4 (UBR4) to ubiquitinate EZH2 at Lys381 and promote its proteasomal degradation. UBR4 depletion reduces EZH2 ubiquitination and prolongs its halflife, whereas restoration of UBE2L6 decreases EZH2 abundance and suppresses melanoma cell growth and invasiveness [17]. UHRF1 therefore functions as an upstream epigenetic suppressor of the UBE2L6/UBR4 pathway rather than as the E3 ligase that directly ubiquitinates EZH2.

EZH2 can also regulate the ubiquitindependent stability of other oncogenic proteins. In neuroblastoma and small cell lung carcinoma, EZH2 associates with MYC or MYCN and protects these proteins from SCF^FBXW7^dependent polyubiquitination and proteasomal degradation. EZH2 depletion consequently accelerates MYC/MYCN turnover and suppresses tumor formation [169]. This mechanism is distinct from the EZH2/TAD/MYC/p300 transcriptional complex: the former regulates MYC/MYCN protein stability, whereas the latter uses EZH2 as a noncanonical transcriptional coactivator.

Together, these mechanisms establish EZH2 as both a substrate and an upstream modulator of ubiquitindependent protein stability. By contrast, EZH2dependent regulation of DRAM1, ATG7, GABARAP, or other ATGs primarily represents transcriptional control of autophagy and does not establish direct ubiquitindependent autophagic degradation of EZH2.

##### 3.5.1.2 Mixed‐lineage leukemia 1 (MLL1)/Lysine Methyltransferase 2A

MLL1, also termed lysine methyltransferase 2A (KMT2A), is an H3K4 methyltransferase that controls developmental, hematopoietic, and lineag‐specific transcriptional programs, including expression of HOX genes. Its abundance is dynamically controlled by the UPS, coupling MLL1dependent transcription with cell cycle progression and differentiation.

During the cell cycle, WT MLL1 undergoes biphasic degradation mediated by SCF^SKP2^ and APC/C^CDC20^. These ligases regulate MLL1 abundance at different cell cycle stages and thereby coordinate MLL1dependent gene expression with orderly cell cycle execution. This regulatory circuit is disrupted in leukemogenic MLL fusion proteins, which are not subjected to the same periodic degradation and consequently sustain aberrant transcriptional programs [77]. Proteolytic cleavage provides an additional regulatory layer: when the cleaved MLL1 N and C terminal subunits dissociate, they are routed through distinct degradation pathways, indicating that complex assembly protects MLL1 from uncontrolled turnover [190].

MLL1 degradation is also regulated during hematopoietic differentiation. Increased expression of the substrate recognition factor ankyrin repeat and SOCS box containing protein 2 (ASB2) promotes elongin B/C Cullin 5 SOCS box complex containing ASB2 (ECS^ASB2^)dependent ubiquitination and proteasomal degradation of MLL1, reducing the expression of MLL1 target genes and facilitating hematopoietic differentiation [78]. Thus, physiological removal of MLL1 can support lineage progression by terminating stem or progenitorassociated transcriptional programs.

In MLL rearranged leukemia, WT MLL1 is additionally destabilized by an inflammatory signaling pathway involving IL‐1, interleukin‐1 receptor‐associated kinases 1 and 4 (IRAK1 and IRAK4), and ubiquitin‐conjugating enzyme E2 O (UBE2O). UBE2O interacts with an internal region of WT MLL1 and promotes its proteasomal degradation but has a weaker effect on MLL fusion proteins lacking the relevant region. Pharmacological inhibition of IRAK signaling stabilizes WT MLL1, allowing it to compete with MLL fusion proteins at selected chromatin targets and reducing leukemic dependence on fusion driven transcription [79]. This mechanism illustrates that stabilization, rather than degradation, of a WT epigenetic regulator can also have therapeutic value.

MLL1 can further alter the ubiquitindependent stability of downstream transcription factors. The MLL1/WDR5 (WD repeat domain 5) complex interacts with SRY box transcription factor 2 (SOX2) and promotes its ubiquitination and proteolysis, potentially involving SOX2 methylation. PHF20L1 associates SOX2 and counteracts MLL1/WDR5associated ubiquitination and degradation, with its malignant brain tumor domain potentially contributing to this protective effect [191]. Because the responsible E3 ligase was not identified, MLL1/WDR5 should be described as increasing SOX2 susceptibility to ubiquitination rather than functioning directly as an E3 ligase.

MLL fusion proteins can additionally be eliminated through an ALP. In MLL rearranged leukemia, lysosome‐associated membrane protein 5 (LAMP5) antisense RNA 1 (LAMP5‐AS1) recruits the H3K79 methyltransferase disruptor of telomeric silencing 1 like (DOT1L) to the LAMP5 locus and increases LAMP5 expression. LAMP5 suppresses autophagic flux and protects MLL fusion proteins from degradation. Depletion of LAMP5‐AS1 or disruption of the LAMP5‐AS1/DOT1L/LAMP5 axis restores autophagy, decreases fusion protein abundance, promotes apoptosis, and prolongs survival in preclinical models [192, 193]. However, ubiquitination of MLL fusion proteins has not been established as the cargo recognition signal; this pathway should therefore be regarded as an alternative lysosomal degradation route rather than a fully defined ubiquitindependent selective autophagy mechanism.

Collectively, MLL1 is regulated by multiple degradationoriented mechanisms involving SCF^SKP2^, APC/C^CDC20^, ECS^ASB2^, and UBE2O. In parallel, MLL1/WDR5 can alter the ubiquitination susceptibility of SOX2, whereas MLL fusion proteins can evade both physiological UPS regulation and autophagic degradation. These mechanisms connect MLL1 protein fate with differentiation, cell cycle control, stemness, and leukemogenesis.

##### 3.5.1.3 Histone Deacetylase 4

Histone deacetylase 4 (HDAC4) is a Class IIa HDAC that functions predominantly as a signalresponsive transcriptional corepressor and protein interaction scaffold. Its nucleocytoplasmic distribution is regulated by phosphorylationdependent binding to 14‐3‐3 proteins, allowing extracellular signals to modulate HDAC4dependent transcription and nonhistone substrate regulation.

HDAC4 itself is subject to signaldependent ubiquitination. In osteoblasts, parathyroid hormone signaling promotes SMURF2dependent ubiquitination and proteasomal degradation of HDAC4. Loss of HDAC4 releases MEF2C from transcriptional repression and increases RANKL expression, linking regulated HDAC4 turnover to osteoclastogenic signaling. Sympathetic signaling, by contrast, favors HDAC4 accumulation and alters its transcriptional interactions [80]. This pathway demonstrates that E3 ligasedependent control of HDAC4 abundance can redirect the transcriptional output of the same cell type.

HDAC4 also regulates the ubiquitination of downstream substrates through acetylation/ubiquitination crosstalk. In nasopharyngeal carcinoma, HDAC4 deacetylates the phosphatase phospholysine phosphohistidine inorganic pyrophosphate phosphatase (LHPP) at Lys6, increasing its association with the E3 ligase TRIM21. TRIM21 subsequently promotes K48linked polyubiquitination and proteasomal degradation of LHPP, removing a tumorsuppressive protein and supporting cancer cell proliferation, invasion, and metastasis [194]. This mechanism illustrates how deacetylation can increase susceptibility to E3 ligasemediated degradation.

HDAC4 additionally regulates gasdermin D (GSDMD) through coordinated acetylation, phosphorylation, and ubiquitination. Acetylation of GSDMD at Lys248 enhances its pyroptotic activity, whereas HDAC4mediated deacetylation reduces GSDMD ubiquitination and suppresses pyroptosis. Protein phosphatase 1dependent dephosphorylation of HDAC4 counteracts its activity toward GSDMD, generating a multilayered posttranslational regulatory circuit [195]. HDAC4 is the deacetylase component of this pathway and should not be interpreted as the E3 ligase responsible for GSDMD ubiquitination.

A more direct intersection with lysosomal protein turnover occurs in GC. HDAC4 represses MEF2Adependent transcription of ATG4B, impairs autophagic flux, and reduces p62/SQSTM1dependent autophagic degradation of mitogen activated protein kinase kinase kinase 3 (MEKK3). Accumulated MEKK3 activates p38 MAPK signaling and promotes GC growth and metastasis [196]. Because the ubiquitin chain architecture on MEKK3 and the precise cargo recognition mechanism were not fully defined, this axis represents p62dependent autophagic turnover but not yet a completely resolved ubiquitindependent selective autophagy pathway.

Thus, HDAC4 participates in proteostasis at several levels: it is itself a SMURF2 substrate, it modifies the susceptibility of LHPP and GSDMD to ubiquitination, and it regulates p62‐dependent MEKK3 turnover through transcriptional control of autophagic flux.

##### 3.5.1.4 HDAC6

HDAC6 is a predominantly cytoplasmic deacetylase whose C terminal ZnF/UBP domain recognizes unanchored ubiquitin C termini associated with protein aggregates. This interaction contributes to ubiquitindependent protein quality control and is not strictly restricted to a specific ubiquitin linkage type.

HDAC6 directly links ubiquitinated misfolded proteins to the aggresome/autophagy pathway. It binds polyubiquitinated protein cargo and dynein motors, enabling retrograde transport of misfolded proteins along microtubules to perinuclear aggresomes. Loss of HDAC6 disrupts aggresome formation and increases sensitivity to proteotoxic stress [197]. HDAC6 also coordinates stress responses to ubiquitinated protein accumulation, including activation of heat shock factordependent proteostasis programs [198].

Following aggresome formation, HDAC6 recruits cortactindependent actin remodeling machinery to ubiquitinated aggregates. The resulting F‐actin network supports autophagosome lysosome fusion and degradation of aggregate‐associated cargo. HDAC6 is therefore required for ubiquitin selective quality control autophagy but is largely dispensable for starvationinduced bulk autophagy [199].

The relationship between HDAC6 and valosin containing protein (also known as p97/VCP) provides an additional mechanism for routing ubiquitinated substrates between proteasomal processing and autophagic clearance. In TMZ resistant GBM cells, HDAC6 and p97/VCP activities are associated with increased reliance on autophagic clearance of ubiquitinated misfolded proteins and adaptation to ER stress. HDAC6 inhibition with tubastatin A disrupts the dynein/HDAC6 machinery, alters ubiquitination and autophagic turnover, and reduces stress tolerance [200]. Because this evidence derives from a pharmacological GBM model, the HDAC6/p97/VCP relationship should not be generalized as a universal binary switch for all substrates.

HDAC6 also regulates the ubiquitin‐binding activity of p62/SQSTM1. Under nutrient stress, TIP60‐dependent acetylation of p62 at Lys420 and Lys435 increases its affinity for ubiquitin, promotes p62 body formation, and facilitates sequestration and autophagic clearance of ubiquitinated proteins. HDAC6 reverses these acetylation events and thereby modulates p62/ubiquitin interactions and aggregate turnover [201].

The p62 UBA domain is itself subject to ubiquitination. Kelch like ECHassociated protein 1 (Keap1)/Cullin 3 (CUL3) ubiquitinates p62 at Lys420, increasing its capacity to sequester ubiquitinated proteins, form inclusions, associate with autophagic machinery, and support aggregate clearance [202]. Thus, p62 cargo recognition is controlled by both ubiquitination and reversible acetylation within the same UBA region regulatory environment. Although Lys420 can undergo either modification, direct competition or ordered switching between ubiquitination and acetylation has not been conclusively established.

Together, the Keap1/CUL3 and TIP60/HDAC6 pathways illustrate how E3 ligase activities and reversible acetylation jointly regulate the ability of p62 to recognize, condense, and deliver ubiquitinated cargo to autophagosomes. Keap1/CUL3 directly modifies the receptor, whereas HDAC6 regulates receptor acetylation, cargo transport, aggresome formation, and autophagosome maturation.

HDAC6 can additionally regulate conventional proteasomal degradation through substrate deacetylation. In colon cancer, HDAC6dependent deacetylation of A‐kinase anchoring protein 12 (AKAP12) enhances its ubiquitination and degradation, reduces AKAP12 abundance, and promotes metastatic phenotypes [203]. HDAC6 is therefore best characterized as a deacetylase, ubiquitin‐binding adaptor, dynein cargo linker, regulator of p62 cargo recognition, and facilitator of quality control autophagy rather than as an E3 ligase.

##### 3.5.1.5 LSD1/KDM1A

LSD1/KDM1A is a flavindependent demethylase that removes H3K4Me1/2 and, in selected transcriptional complexes, H3K9Me1/2. Its abundance is controlled by competing E3 ligase and DUB activities, allowing changes in LSD1 stability to regulate differentiation, stemness, epithelialmesenchymal transition (EMT), and tumor progression.

During neural differentiation, jade family PHD finger 2 (JADE2) promotes LSD1 polyubiquitination and proteasomal degradation. Removal of LSD1 relieves repression of proneural genes and facilitates neurogenesis [204]. SCF complex containing F‐box protein 24 (FBXO24) (SCF^FBXO24^) provides a second degradation pathway: FBXO24 recognizes LSD1, promotes its ubiquitination and proteasomal turnover, and suppresses LSD1dependent breast cancer proliferation, EMT, cancer stem cell (CSC) like traits, and tumorigenesis [205]. These findings demonstrate that E3 ligasemediated LSD1 degradation can support physiological differentiation or constrain oncogenic chromatin programs.

Several DUBs generate the opposite outcome. USP7 interacts with LSD1, reduces its ubiquitination, and stabilizes the protein in GBM, thereby supporting tumor cell proliferation, invasion, and suppression of p53 signaling [206]. Nuclear GSK3β phosphorylates LSD1 and promotes its association with USP22; USP22mediated deubiquitination stabilizes LSD1 and maintains glioma stem cell properties [207]. USP28 similarly deubiquitinates and stabilizes LSD1 in breast cancer, sustaining stem celllike characteristics and tumorigenicity [208]. Together, these mechanisms demonstrate that several cancer‐associated DUBs converge on LSD1 but operate in distinct signaling and cellular contexts.

LSD1 stability is further controlled by crosstalk between arginine methylation and deubiquitination. CARM1 asymmetrically dimethylates LSD1 at Arg838, enhancing its interaction with USP7 and promoting USP7dependent deubiquitination and stabilization. Stabilized LSD1 represses E‐cadherin and activates vimentin transcription through locusspecific H3K4Me2 and H3K9Me2 demethylation, thereby promoting breast cancer invasion and metastasis [209]. More recently, lactate driven LSD1 lactylation was shown to enhance its interaction with FOSlike 1, AP‐1 transcription factor subunit (FosL1) and protect it from TRIM21mediated degradation in BRAF/MEK inhibitor resistant melanoma [210]. Stabilized lactylated LSD1 cooperates with FosL1 to repress TFRCdependent iron uptake and ferroptosis, thereby supporting acquired therapeutic resistance. These pathways illustrate how distinct metabolic or enzymatic modifications can create protective interaction interfaces that favor DUB recruitment or prevent E3 ligasedependent turnover.

OTUD7B provides an additional link between ubiquitin chain editing, LSD1 complex integrity, and protein turnover. OTUD7B removes K63linked polyubiquitin chains from LSD1 at Lys226 and Lys277, thereby preserving the integrity of the LSD1/REST corepressor 1 (CoREST) complex. Loss of OTUD7B increases K63linked LSD1 ubiquitination, disrupts its association with CoREST, and promotes p62/SQSTM1dependent proteasomal degradation of LSD1. Consequently, OTUD7B deficiency reduces genomewide LSD1 occupancy and suppresses breast cancer invasion and metastasis [211]. This mechanism illustrates that p62dependent recognition of K63linked ubiquitin chains can direct proteasomal degradation rather than necessarily indicating selective autophagy.

Ubiquitination can also regulate LSD1 activity without altering its abundance. In NSCLC, TRIM35mediated K63‐linked ubiquitination of LSD1 at Lys422 suppresses its demethylase activity without promoting degradation [148]. This nondegradative modification also influences antitumor immunity through downstream regulation of IFNGR1 autophagic turnover, without implying autophagic degradation of LSD1 itself.

Although LSD1 activity has been associated with autophagyrelated transcription and neurological disease, current evidence does not establish direct ubiquitin‐dependent autophagic degradation of LSD1.

Collectively, LSD1 is regulated through degradation orientedubiquitination, stabilizing deubiquitination, nondegradative K63linked ubiquitination, and crosstalk with methylation and lactylation. JADE2 and SCF^FBXO24^ promote LSD1 proteasomal turnover, whereas USP7, USP22, USP28, and OTUD7B preserve its abundance, complex integrity, or chromatin occupancy. TRIM35‐mediated K63linked ubiquitination instead suppresses catalytic activity without inducing degradation. These mechanisms demonstrate that ubiquitin signals control not only LSD1 abundance but also its binding partner specificity, enzymatic output, downstream autophagyrelated signaling, and disease‐associated transcriptional programs.

##### 3.5.1.6 Lysine Demethylase 4A (KDM4A)/Jumonji DomainContaining Protein 2A

KDM4A is a Jumonji C domain containing demethylase that removes H3K9Me2/3 and H3K36Me2/3. Its abundance is controlled by several SCF family E3 ligase complexes. Under basal conditions, SCF complex containing FBXO22 (SCF^FBXO22^) promotes KDM4A ubiquitination and proteasomal degradation, thereby regulating global H3K9 and H3K36 methylation and coordinating KDM4A abundance with cell cycle progression [212]. SCF complex containing FBXL4 (SCF^FBXL4^) independently contributes to cell cycledependent KDM4A turnover and prevents inappropriate accumulation of demethylase during S phase progression [213]. These parallel SCF pathways indicate that basal KDM4A abundance is controlled by more than one substrate recognition module.

KDM4A degradation also plays a direct role in DNA DSB responses. Through its tandem Tudor domains, KDM4A recognizes H4K20Me2 and competes with 53BP1 for access to this pre‐existing histone mark. Following DNA damage, RNF8 and RNF168dependent ubiquitination promotes KDM4A degradation, exposes H4K20Me2, and enables efficient recruitment of 53BP1 to damaged chromatin [214]. KDM4A turnover therefore regulates both demethylase abundance and the accessibility of pre‐existing histone modification to a DNA repair reader.

Conversely, USP7 directly deubiquitinates and stabilizes KDM4A in bladder cancer. The USP7/KDM4A axis promotes tumor cell proliferation, supports the DDR, and reduces cisplatin sensitivity. Genetic depletion or pharmacological inhibition of USP7 increases KDM4A ubiquitination and proteasomal degradation, aggravates cisplatin‐induced DNA damage, and improves treatment response [215]. KDM4A loss following USP7 depletion is reversed by the proteasome inhibitor MG132 but not by chloroquine‐mediated autophagy inhibition, demonstrating that this axis controls proteasomal rather than autophagy lysosome turnover.

Collectively, KDM4A abundance is determined by opposing E3 ligase and DUB activities. SCF^FBXO22^, SCF^FBXL4^, RNF8, and RNF168 promote its ubiquitination and proteasomal degradation under basal, cell cycle, or DNA damage conditions, whereas USP7 preserves KDM4A abundance in bladder cancer. These mechanisms connect KDM4A protein turnover to global histone methylation, S phase control, 53BP1 recruitment, genome stability, and chemotherapy resistance. Current evidence does not establish a direct autophagydependent pathway for KDM4A degradation.

#### 3.5.2 ATPDependent Chromatin Remodelers: UbiquitinDependent Turnover and Nondegradative Signaling

Whereas histone modifying enzymes establish or remove covalent chromatin marks, ATPdependent chromatin remodeling complexes determine how these marks are presented within nucleosomal structures. These complexes use the energy derived from ATP hydrolysis to reposition, restructure, or evict nucleosomes, thereby regulating DNA accessibility to transcription, replication, and DNA repair machinery. Major remodeler families include switch/sucrose nonfermentable (SWI/SNF), imitation switch (ISWI), chromodomain helicase DNA binding (CHD), and inositol requiring 80 (INO80), which differ in subunit composition, genomic localization, and remodeling output [216]. Their functions depend not only on ATPase activity but also on the abundance, assembly, chromatin recruitment, and interaction networks of individual subunits. Ubiquitination can therefore regulate chromatin remodeling through both degradationoriented and nondegradative mechanisms. Among these remodelers, mammalian SWI/SNF complexes are particularly relevant to disease because genes encoding their subunits are collectively mutated in multiple human cancers, making mammalian SWI/SNF one of the most frequently disrupted chromatin regulatory machineries in malignancy [217, 218].

Autophagy can also influence chromatinassociated ubiquitin signaling without directly degrading a chromatin remodeler. In autophagydeficient cells, accumulated p62/SQSTM1 enters the nucleus and binds RNF168, thereby suppressing RNF168dependent H2A ubiquitination after DNA damage. Reduced chromatin ubiquitination subsequently impairs the recruitment of RAP80, BRCA1, RAD51, and other repair factors to DNA DSBs [219]. This mechanism provides a direct intersection among autophagic turnover, ubiquitindependent chromatin signaling, and genome maintenance. However, it should not be interpreted as evidence that chromatin remodeling proteins generally undergo p62dependent autophagic degradation.

##### 3.5.2.1 SWI/SNFRelated, MatrixAssociated, ActinDependent Regulator of Chromatin Subfamily A Member 4/BrahmaRelated Gene 1 and SWI/SNF

SWI/SNFrelated, matrixassociated, actindependent regulator of chromatin subfamily A member 4 (SMARCA4), also termed Brahmarelated gene 1 (BRG1), is one of the two mutually exclusive catalytic ATPase subunits of mammalian SWI/SNF complexes, the other being SMARCA2/BRM. SMARCA4 uses ATP hydrolysis to alter nucleosome positioning and chromatin accessibility and functions within canonical BRG1/BRMassociated factor (BAF), polybromoassociated BAF, and noncanonical BAF assemblies. Beyond local nucleosome repositioning, SMARCA4 regulates higher order chromatin contacts and domain scale regulatory accessibility. Loss or restoration of SMARCA4 can therefore reorganize broad chromatin domains while producing more selective downstream transcriptional responses [220, 221]. Its biological output is highly context dependent: SMARCA4 frequently acts as a tumor suppressor when genetically lost, whereas elevated or stabilized SMARCA4 can sustain oncogenic transcription in selected cellular settings.

SWI/SNF complex stability is regulated by ubiquitindependent turnover at both catalytic and structural subunit levels. The E3 ligase checkpoint with forkhead and RING finger domains (CHFR) interacts with BRG1, SMARCB1/SNF5, and BAF60A and promotes their ubiquitination and proteasomal degradation. The core BAF subunit BAF155, termed SRG3 in mice, counteracts this process by limiting the interaction of these components with CHFR and thereby stabilizing the assembled remodeling complex [81]. This mechanism illustrates how incorporation into a multisubunit complex can protect chromatin remodelers from E3 ligasemediated degradation.

A more substratespecific degradation pathway operates in GC. CK1δ phosphorylates BRG1 at Ser31 and Ser35, generating a phosphodegron recognized by the SCF^FBXW7^ ubiquitin ligase complex. FBXW7 subsequently promotes BRG1 ubiquitination and proteasomal degradation. Loss of FBXW7 or disruption of the phosphodegron stabilizes BRG1, reduces E‐cadherin expression, and promotes GC cell migration and metastasis [82]. Thus, phosphorylationdependent ubiquitination can convert upstream signaling into changes in SWI/SNF abundance and chromatin output.

BRG1 can also undergo nondegradative ubiquitination. In Wnt responsive cells, the HECT family E3 ligase thyroid hormone receptor interactor 12 (TRIP12) ubiquitinates BRG1 and enhances its association with nuclear β‐catenin without disrupting its incorporation into the SWI/SNF complex. The resulting BRG1/β‐catenin interaction promotes SWI/SNF recruitment to Wnt target genes and supports transcriptional activation [222]. This pathway demonstrates that ubiquitination of a remodeling ATPase can regulate transcription factor engagement and genomic targeting rather than protein destruction.

A related nondegradative mechanism has been identified for SWI/SNFrelated, matrix‐associated, actindependent regulator of chromatin, subfamily A like 1 (SMARCAL1), a SNF2 family DNA translocase that remodels stalled replication forks but is distinct from canonical SMARCA4 containing BAF complexes. Following replication stress, RING finger and WD repeat domain 3 (RFWD3) directly ubiquitinates SMARCAL1. This modification does not trigger SMARCAL1 degradation; instead, it weakens the interaction of SMARCAL1 with replication protein A (RPA)coated single stranded DNA, limits excessive fork remodeling and MUS81dependent cleavage, and thereby preserves replication fork stability [223]. The modified substrate in this pathway is therefore SMARCAL1 rather than SMARCA4.

Together, these findings establish several distinct modes of SWI/SNFrelated ubiquitin regulation. CHFR and SCF^FBXW7^ control complex abundance through proteasomal degradation, TRIP12 alters BRG1 recruitment to β‐cateninresponsive chromatin, and RFWD3 regulates SMARCAL1 residence at stalled replication forks without reducing its abundance.

##### 3.5.2.2 ATRich Interaction Domain 1A

ATrich interaction domain 1A (ARID1A) is a large structural and targeting subunit of canonical BAF complexes. Its ARID domain contributes to DNA interaction, whereas extensive intrinsically disordered regions support assembly with transcription factors and other SWI/SNF components. ARID1A is particularly important for maintaining enhancer accessibility and coordinating tumor suppressive transcriptional programs [224].

ARID1A abundance is directly controlled by opposing ubiquitination and deubiquitination. In SCC, the E3 ligase TRIM32 binds ARID1A and promotes its ubiquitination and proteasomal degradation. The DUB USP11 counteracts TRIM32 by removing ubiquitin from ARID1A and stabilizing the protein. Disruption of this balance alters ARID1A‐dependent expression of syndecan‐2 and affects cancer cell stemness, proliferation, metastasis, and chemoresistance [83]. The TRIM32/USP11 axis therefore represents a clear example of an E3/DUB pair directly determining the stability and functional output of a chromatin remodeling subunit.

ARID1A turnover is also coupled with DNA damage signaling. In GC cells, DNA damage activates an ataxia telangiectasia mutated (ATM)dependent SCF^β‐TrCP^ pathway that promotes ARID1A ubiquitination and proteasomal degradation. β‐TrCP recognizes a phosphorylated DSGXXS degron in ARID1A, with Ser1316 and Ser1320 being required for substrate recognition and degradation. Genetic or pharmacological inhibition of ATM impairs DNA damage‐induced interaction between ARID1A and β‐TrCP and stabilizes ARID1A. However, whether ATM directly phosphorylates these residues or acts through an intermediate kinase remains unresolved [84]. This pathway links genotoxic stress to the regulated turnover of a SWI/SNF targeting subunit.

Oncogenic nutrient signaling provides an additional mechanism of ARID1A regulation. In HCC, activated mTORC1 interacts with ARID1A and promotes its ubiquitination and proteasomal degradation. The resulting mTORC1/ARID1A axis drives oncogenic chromatin remodeling and YAPdependent transcription, thereby enhancing liver cancer cell growth and tumor development. Conversely, increased ARID1A expression counteracts AKT driven liver tumorigenesis. Dysregulation of this pathway also contributes to resistance to mTORtargeted therapies, linking metabolic signaling, chromatin remodeling, and therapeutic response [85].

Collectively, ARID1A abundance is regulated primarily by UPSdependent mechanisms: the TRIM32/USP11 axis, an ATM activated SCF^β‐TrCP^ pathway triggered by DNA damage, and mTORC1associated ubiquitination. These mechanisms link oncogenic, metabolic, and genotoxic signals to the stability of ARID1A, a tumor suppressive chromatin remodeler, thereby shaping malignant transcriptional programs, tumor progression, and treatment response.

##### 3.5.2.3 Chromodomain Helicase DNABinding Protein 4/Nucleosome Remodeling and Deacetylase

Chromodomain helicase DNAbinding protein 4 (CHD4) is an ATPdependent chromatin remodeling enzyme and a core catalytic component of the nucleosome remodeling and deacetylase (NuRD) complex. CHD4 contains tandem plant homeodomain fingers, tandem chromodomains, and a central SNF2like ATPase motor. These structural elements cooperate in histone and nucleosome recognition and regulate ATP‐dependent DNA translocation and nucleosome remodeling. Within the NuRD complex, CHD4mediated nucleosome repositioning is coordinated with HDAC1/2‐dependent histone deacetylation, thereby contributing to the regulation of chromatin accessibility, transcription, and genome stability [225].

CHD4 abundance is directly regulated by ubiquitindependent proteolysis. In triple negative breast cancer (TNBC), FBXW7, the substrate recognition component of an SCF type E3 ligase, binds CHD4 and promotes its ubiquitination and proteasomal degradation. Reduced FBXW7 expression stabilizes CHD4, promotes β‐catenin nuclear accumulation, and activates Wnt/β‐catenin signaling. Consequently, loss of the FBXW7/CHD4 regulatory axis enhances EMT, CSC properties, self‐renewal, tumor initiation, and tumor progression. Conversely, FBXW7 restoration decreases CHD4 abundance and suppresses TNBC stemness and tumorigenicity [86].

A distinct CHD4 stabilizing mechanism involves the circular RNA WBSCR22 (circWBSCR22). In CRC, circWBSCR22 binds up‐frameshift protein 1 (UPF1), an RNA helicase with ATPase activity, thereby interfering with UPF1dependent ubiquitination and degradation of CHD4. The resulting stabilization of CHD4 promotes CRC cell proliferation, EMT, invasion, and metastatic progression, whereas circWBSCR22 depletion restores CHD4 turnover and suppresses tumor growth and metastasis [87].

Additional studies illustrate the pathological consequences of increased CHD4 abundance or activity without establishing further direct CHD4 ubiquitination mechanisms. In CRC, elevated CHD4 expression is associated with malignant progression, increased cell motility, tumor growth, and reduced sensitivity to platinumbased chemotherapy. CHD4 depletion suppresses tumor growth in vivo, whereas combined treatment with an HDAC1 inhibitor and platinum compounds reduces CHD4 expression and increases platinuminduced cytotoxicity [226]. In GC, CHD4 promotes acquired chemoresistance by facilitating MEK1/2/ERK1/2 interaction, sustained MEK/ERK signaling, and multidrug efflux, thereby reducing intracellular cisplatin accumulation [227]. CHD4 also promotes GC metastasis through the myosin heavy chain 9 (MYH9)/GSK3β/β‐catenin axis. In this pathway, CHD4 facilitates MYH9dependent phosphorylation and ubiquitinationmediated degradation of GSK3β, leading to β‐catenin stabilization, Wnt pathway activation, and EMT [228]. Importantly, the latter mechanism concerns ubiquitination of the downstream substrate GSK3β rather than ubiquitinationdependent turnover of CHD4 itself.

Collectively, current evidence identifies FBXW7mediated proteasomal degradation and circWBSCR22regulated, UPF1dependent ubiquitination as the principal documented mechanisms controlling CHD4 protein stability. By contrast, DUBs that directly remove ubiquitin from CHD4 have not yet been clearly identified. Dysregulation of CHD4 turnover or accumulation can reinforce Wnt/β‐catenin signaling, CSC properties, EMT, metastasis, and chemoresistance. These findings establish CHD4 as a ubiquitinregulated chromatin remodeler while emphasizing the distinction between direct regulation of CHD4 stability and downstream signaling pathways controlled by CHD4.

Ubiquitination regulates ATPdependent chromatin remodeling through both degradative and nondegradative mechanisms. Degradative ubiquitination controls remodeler abundance and complex composition: CHFR targets BAF subunits; SCF^FBXW7^ regulates BRG1 and CHD4 turnover; TRIM32 and β‐TrCP drive ARID1A degradation; and UPF1dependent ubiquitination modulates CHD4 stability. DUBs counterbalance these events; for example, USP11 stabilizes ARID1A. Nondegradative ubiquitination, in turn, regulates remodeler activity, protein interactions, and chromatin engagement: TRIP12mediated ubiquitination enhances BRG1/β‐catenin binding and recruitment to Wnt responsive loci, while RFWD3mediated ubiquitination displaces SMARCAL1 from RPAcoated DNA to prevent aberrant fork remodeling. Thus, ubiquitination outcomes depend on the target remodeler, ubiquitin chain topology, regulatory cofactors, and local chromatin context (Figure 3).

**FIGURE 3:**
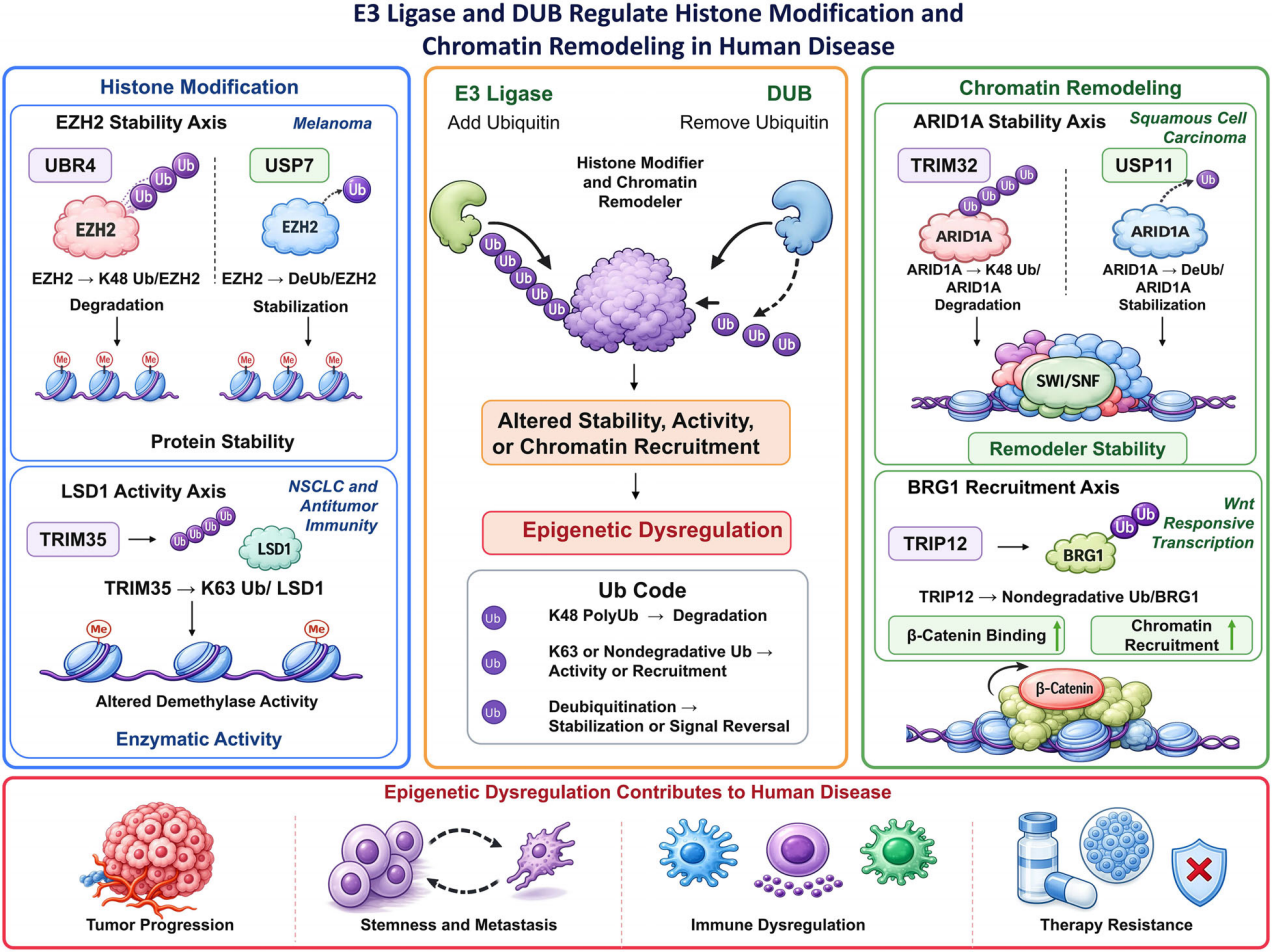
E3 ligases and DUBs regulate histone modification and chromatin remodeling in human disease. This schematic shows ubiquitindependent regulation of histone modifiers and chromatin remodelers. E3 ligases promote the degradation of regulators like EZH2 and ARID1A; DUBs stabilize them by reversing ubiquitination. Nondegradative ubiquitination modulates their chromatin recruitment and activity. Together, these mechanisms shape epigenetic regulation, tumor progression, immune responses, and therapy resistance.

Across the cellular networks discussed above, ubiquitindependent regulation cannot be interpreted solely in terms of protein degradation or stabilization. For example, USP15 removes an activating monoubiquitin mark from TET2, reducing its chromatin association and enzymatic output, whereas USP21 deubiquitinates and stabilizes TET2 in cigarette smoke exposed airway epithelial cells [51, 74]. These examples illustrate that deubiquitination can attenuate substrate activity or promote substrate stability, depending on the ubiquitin modification involved and the cellular context. More broadly, comparing ubiquitindependent changes in protein abundance, activity, recruitment, and molecular interactions reveals shared regulatory principles across distinct cellular networks (Table 2).

**TABLE 2:** Disease impact of ubiquitindependent enzyme regulation.

Enzymes	E3 ligases	Deubiquitinating enzymes	Diseases	Disease impact of Ub‐dependent enzyme regulation	Underlying ubiquitin‐dependent regulatory mechanism
DNMT1	RNF180	/	GC	Suppressing (E3mediated degradation) [66]	/
	UBE3A	/	Hepatic steatosis	Suppressing (E3mediated degradation) [67]	/
DNMT3A	CRL4^DCAF8^	/	Leukemia (DNMT3A destabilizing variants)	Promoting (variantspecific E3mediated degradation) [68]	DCAF8dependent degradation of unstable DNMT3A variants
TET2	Unidentified	/	HCC	Promoting (kinaseprimed ubiquitindependent degradation) [71]	FGFR3Δ7–9dependent Tyr1902 phosphorylation
	Unidentified	USP21	Cigarette smoke‐induced airway injury	Suppressing (DUBmediated stabilization) [74]	USP21 mediated TET2 deubiquitination and stabilization; USP21 loss exacerbates airway injury
	CRL4^VprBP^	/	HIV‐1 infection	Promoting (E3mediated degradation) [75]	Vpr mediated CRL4^VprBP^ recruitment
EZH2	UBR4	/	Melanoma	Suppressing (E3mediated degradation) [17]	Lys381 ubiquitination
	SCF^FBXW7^	/	Inflammatory bowel disease	Promoting (E3mediated degradation) [76]	K48linked polyubiquitination
MLL1	UBE2O (E2/E3 hybrid enzyme)	/	MLL rearranged leukemia	Promoting (E2/E3mediated degradation) [79]	UBE2Obinding internal region
SMARCA4	SCF^FBXW7^	/	GC	Suppressing (E3mediated degradation) [82]	Ser31/Ser35 phosphodegron
ARID1A	TRIM32	USP11	SCC	Contextdependent (E3/DUBdependent regulation) [83]	TRIM32mediated ARID1A degradation promotes SCC progression, whereas USP11mediated stabilization suppresses SCC progression.
	SCF^β‐TrCP^	/	GC	Not established (E3mediated degradation) [84]	Phosphorylated DSGXXS degron at Ser1316/Ser1320
	Unidentified (mTORC1‐associated)	/	HCC	Promoting (mTORC1associated ubiquitindependent degradation) [85]	mTORC1associated ubiquitination
CHD4	SCF^FBXW7^	/	TNBC	Suppressing (E3mediated degradation) [86]	/
	Unidentified (UPF1 dependent)	/	CRC	Promoting (inhibition of CHD4 degradation) [87]	circWBSCR22 blocks UPF1dependent ubiquitination and CHD4 degradation
METTL3	TRIM21	/	PDAC	Suppressing (E3mediated degradation) [88]	K48linked polyubiquitination
	STUB1	/	Alcohol‐associated steatohepatitis	Promoting (E3mediated degradation) [89]	STUB1mediated K48‐linked polyubiquitination and proteasomal degradation
	FBXW7	/	HCC	Promoting (O‐GlcNAcylationmediated protection from degradation) [90]	O‐GlcNAcylation at Thr186/Ser192/Ser193 limits FBXW7 binding
METTL14	HRD1	/	ER proteotoxic liver disease	Promoting (HRD1mediated METTL14 degradation) [92]	ER stress suppresses HRD1mediated METTL14 ubiquitination and ERAD, allowing METTL14 accumulation and promoting m^6^Adependent CHOP mRNA decay.
WTAP	Unidentified	USP7	GBM	Promoting (DUBmediated stabilization) [173]	O‐GlcNAcylationdependent stabilization and USP7associated deubiquitination
FTO	Unidentified	USP7	PDAC	Promoting (DUBmediated stabilization) [94]	USP7mediated deubiquitination and stabilization
	DTX2	/	Melanoma (immunotherapy resistance)	Suppressing (ubiquitindependent degradation) [95]	DTX2/UFD1mediated recognition
	Unidentified	/	Arsenicassociated tumorigenesis	Suppressing (p62mediated selective autophagy) [97]	p62/SQSTM1 recognition of ubiquitinated FTO
ALKBH5	Unidentified	USP36	GBM	Promoting (DUBmediated stabilization) [98]	USP36mediated deubiquitination and stabilization
	HECW2	USP14	GBM radiotherapy resistance	Suppressing (HECW2mediated degradation); promoting (USP14mediated stabilization) [100]	HECW2mediated Lys295 ubiquitination; MST4dependent Ser64/Ser69 phosphorylation promotes USP14mediated stabilization
	RNF180	/	UC	Promoting (E3mediated degradation) [101]	/
NAT10	ZSWIM6	/	Head and Neck SCC	Suppressing (E3‐mediated degradation) [102]	RNPS1 Blocks ZSWIM6mediated Degradation; NAT10 Stabilization Promotes Tumor Progression
	Unidentified	USP39 (reported)	Inflammationassociated cardiac dysfunction	Promoting (USP39associated stabilization) [103]	USP39associated NAT10 stabilization; Lys195/Lys426 ubiquitination
ADAR1	SCF^β‐TrCP^	/	HER2positive breast cancer	Suppressing (E3mediated degradation) [105]	LINC00624 blocks β‐TrCPmediated ADAR1 degradation; ADAR1 stabilization promotes tumor progression and therapy resistance

## 4 Dysregulation of Ubiquitination and Deubiquitination in Human Diseases

Dysregulation of ubiquitination and deubiquitination contributes to human disease by altering the abundance, localization, activity, or interactions of key regulatory proteins. Diseaseassociated defects arise from mutations or dysregulated expression of E3 ligases and DUBs; impaired substrate recognition; faulty ubiquitin decoding; defective proteasomal degradation; or misrouting of ubiquitinated cargo, converting normally reversible regulatory signals into persistent oncogenic, neurotoxic, or inflammatory states.

The pathological consequences of ubiquitination/deubiquitination dysregulation are highly context dependent. In cancer, altered protein turnover promotes oncogene stabilization, tumor suppressor loss, epigenetic plasticity, immune evasion, and therapy resistance. In neurodegenerative diseases, defective ubiquitindependent quality control causes the accumulation of aggregation prone proteins and damaged mitochondria. In inflammatory and autoimmune disorders, failure to terminate ubiquitindependent signaling sustains cytokine production, interferon responses, inflammasome activation, and pathogenic lymphocyte expansion. The following sections therefore focus on diseaseassociated abnormalities and phenotypic consequences of representative ubiquitination/deubiquitination networks.

UPS dysfunction extends beyond cancer, neurodegeneration, and autoimmunity to conditions such as skeletal muscle atrophy, where hyperactivation of the UPS and ALP drives protein loss [229]. In a mouse model of Type 1 diabetesassociated sarcopenia, receptorinteracting protein kinase 3 deficiency improved proteostasis by attenuating inflammatory and cellular stress responses and limiting overactivation of both the UPS and ALP [230]. These findings illustrate the contribution of dysregulated proteostasis to skeletal muscle loss.

### 4.1 Cancer: Aberrant Protein Stability, Cellular Plasticity, and Therapy Resistance

Cancerassociated dysregulation of ubiquitination/deubiquitination frequently converges on oncogenes, tumor suppressors, and apoptotic regulators. The p53, MYC, and MCL1 networks provide representative examples. Increased p53 turnover weakens cell cycle arrest, senescence, DNA repair, and apoptosis, whereas stabilization of MYC sustains proliferationassociated transcriptional and metabolic programs. Excessive accumulation of the antiapoptotic protein MCL1 raises the threshold for mitochondrial apoptosis and promotes resistance to cytotoxic and targeted therapies [62, 156, 160, 164, 165]. These observations illustrate that malignant transformation does not necessarily require complete pathway inactivation. Relatively modest changes in protein halflife may provide a selective advantage by prolonging oncogenic signaling, delaying tumor suppressive responses, or preserving survival proteins during therapeutic stress.

Ubiquitination/deubiquitination dysregulation also promotes epigenetic plasticity by altering DNA methylation and demethylation programs. In CCA, STUB1mediated K63linked ubiquitination of UHRF1 enhances its nuclear localization and cooperation with DNMT1, reinforcing promoter hypermethylation and malignant progression. Conversely, RNF180dependent ubiquitination and degradation of DNMT1 restrain aberrant promoter methylation and suppress GC proliferation and metastasis [66, 125]. Diseaseassociated DNMT3A abnormalities further demonstrate that altered protein stability and impaired catalytic function can converge on similar malignant methylation phenotypes through distinct mechanisms. Structurally unstable DNMT3A variants undergo CUL4/DCAF8dependent degradation, whereas the recurrent R882 mutation primarily disrupts oligomerization and catalytic activity [68, 129]. These alterations disturb lineagespecific methylation programs and contribute to clonal expansion and leukemic transformation.

TET2 provides a complementary example on the DNA demethylation side. In HCC, FGFR3Δ7–9induced phosphorylation promotes TET2 ubiquitination and degradation, reducing PTEN promoter hydroxymethylation and activating AKT signaling [71]. TET2 can also be functionally inhibited without being eliminated: USP15 removes an activating monoubiquitin modification from TET2, suppressing chemokine and interferonassociated transcription and contributing to an immunosuppressive tumor microenvironment [51]. Thus, cancerassociated ubiquitin dysregulation can disable an epigenetic tumor suppressor through either degradation or reversal of a nondegradative activating signal.

Altered turnover of histonemodifying enzymes further reinforces oncogenic chromatin states. Opposing ubiquitination and deubiquitination regulate the abundance of EZH2, LSD1/KDM1A, and KDM4A, influencing transcriptional repression, EMT, CSC properties, the DDR, ferroptosis, invasion, and chemotherapy resistance [17, 18, 205, 208, 210, 212, 215]. These effects are highly contextdependent because ubiquitin signaling may change protein abundance, chromatin occupancy, catalytic activity, or interaction with transcriptional partners. Accordingly, the same chromatin regulator may support different malignant programs across tumor lineages.

KMT2A/MLL rearranged leukemia illustrates a distinct mechanism in which an oncogenic chromatin regulator escapes lysosomal turnover. DOT1Ldependent induction of LAMP5 suppresses autophagic degradation of MLL fusion oncoproteins and preserves leukemiadriving transcriptional programs [192]. The long noncoding RNA LAMP5‐AS1 reinforces this pathway, whereas disruption of the LAMP5‐AS1/DOT1L/LAMP5 axis restores MLL fusion protein clearance [193]. This mechanism demonstrates that malignant cells may maintain an oncogenic protein not only by preventing proteasomal degradation but also by suppressing lysosomal turnover.

Chromatin remodeling complexes constitute another important point of vulnerability. ARID1A is destabilized by TRIM32 and protected by USP11 in SCC, whereas mTORC1‐associated ARID1A degradation promotes oncogenic chromatin remodeling and YAPdependent transcription in HCC [83, 85].

CHD4 and SMARCA4/BRG1 further illustrate how remodeler dosage and ubiquitindependent functional remodeling influence cancer progression. FBXW7dependent degradation of CHD4 restrains Wnt/β‐catenin signaling and CSC properties in TNBC, whereas increased CHD4 stability or activity is associated with EMT, metastatic progression, and chemotherapy resistance in CRC and GC [86, 87, 226, 227, 228]. BRG1 degradation can regulate metastatic behavior, while nondegradative ubiquitination enhances its interaction with β‐catenin and recruitment to Wnt responsive chromatin [82, 222]. Ubiquitination therefore controls chromatin remodeling not only by determining remodeler abundance but also by altering molecular interactions and genomic targeting.

Regulation by ubiquitination and deubiquitination of RNA modifying proteins provides an additional source of cancer cell plasticity. Stabilization of the METTL3/METTL14/WTAP writer complex can enhance m^6^A deposition, whereas degradation of individual writer components may reprogram transcript stability, translation, ferroptosis, and antitumor immunity [19, 88, 89, 93]. The abundance of the demethylases FTO and ALKBH5 is similarly controlled by opposing E3 ligases and DUBs, linking their stability to treatment resistance, DNA repair, CSC maintenance, and immune regulation [94, 95, 98, 99, 100]. Ubiquitindependent regulation of YTH domain readers further changes the translation or decay of selected m^6^Amodified transcripts, thereby affecting cell cycle progression, metastatic behavior, metabolic adaptation, and responses to immunotherapy [174, 177, 178, 181, 182, 183, 184].

Beyond m^6^A, ubiquitindependent regulation of other RNA modifying enzymes can enhance oncogenic translation or immune escape. RNPS1 protects the ac^4^C writer NAT10 from ZSWIM6mediated degradation, sustaining tRNA ac^4^C modification and cancerassociated protein synthesis [102]. The RNA editing enzyme ADAR1 can likewise be stabilized through SMURF2dependent regulation or protected from β‐TrCPassociated degradation by LINC00624, reinforcing immunosuppressive RNA editing programs and resistance to targeted treatment [104, 105]. These findings indicate that altered protein turnover can reshape the cancer transcriptome and proteome without requiring mutation or transcriptional upregulation of the corresponding RNA modifying enzyme.

A major consequence shared by these pathways is therapeutic adaptation. Ubiquitination/deubiquitination dysregulation can stabilize antiapoptotic proteins, preserve CSC states, enhance DNA repair, suppress ferroptosis, alter drug efflux, or attenuate antitumor immunity. Such changes may produce intrinsic resistance before treatment or emerge through therapeutic selection. Moreover, an E3 ligase or DUB cannot be classified as uniformly oncogenic or tumor suppressive because its biological output depends on substrate availability, tissue lineage, subcellular localization, oncogenic background, and the functional integrity of the UPS.

Overall, cancerassociated ubiquitination/deubiquitination dysregulation promotes malignancy through several convergent outcomes: prolonged oncogenic signaling, loss of tumorsuppressive checkpoints, resistance to apoptosis, epigenetic and transcriptional plasticity, altered RNA metabolism, immune escape, and treatment resistance. The decisive factor is not simply whether a substrate is ubiquitinated, but whether diseaseassociated changes redirect it toward degradation, stabilization, altered localization, molecular interactions, or nondegradative signaling. These dependencies provide the biological rationale for therapeutic targeting of E3 ligases, DUBs, and induced protein degradation pathways (Figure 4).

**FIGURE 4:**
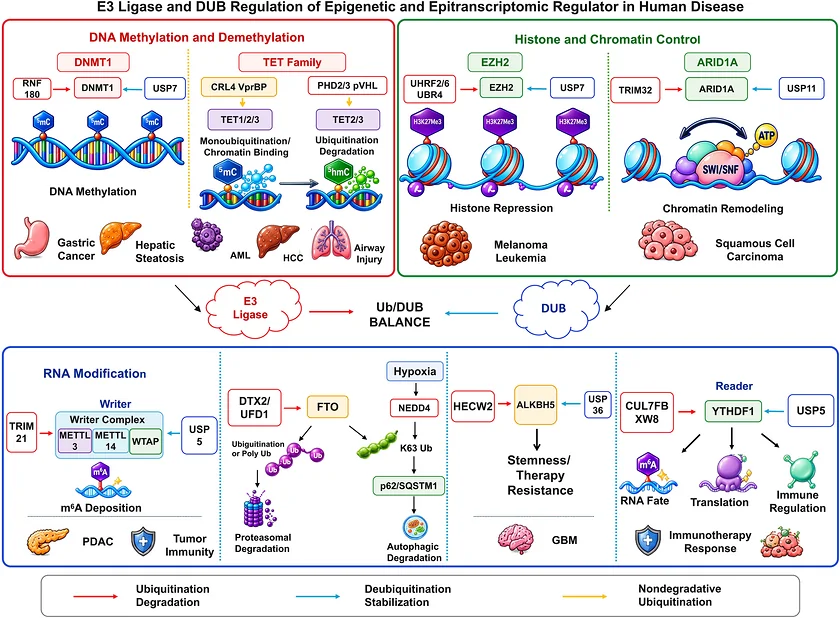
E3 ligase and DUB regulation of epigenetic and epitranscriptomic regulators in human disease. This schematic integrates E3 ligase and DUB regulation of epigenetic and epitranscriptomic regulators, including DNA methylation enzymes, histone/chromatin modifiers, and m^6^A RNA modifiers. E3 ligases drive proteasomal or autophagic degradation, or nondegradative ubiquitination; DUBs stabilize proteins or reverse ubiquitin signals. Their balance governs protein stability, chromatin dynamics, RNA fate, immune responses, and disease progression.

### 4.2 Neurodegenerative Diseases: Proteostasis Failure and PTEN‐induced kinase 1 (PINK1)/ParkinDependent Mitophagy

Neurodegenerative diseases are characterized by progressive neuronal dysfunction and loss accompanied by protein aggregation, mitochondrial damage, oxidative stress, defective intracellular transport, and chronic neuroinflammation. Because neurons are longlived, postmitotic, and highly dependent on polarized transport and energy production, disruption of UPS, ubiquitindependent cargo delivery, or autophagic lysosomal clearance can progressively impair synaptic function and neuronal survival. These defects contribute to the accumulation of amyloid‐β and tau in Alzheimer's disease (AD), α‐synuclein (α‐syn) in Parkinson's disease (PD), and TAR DNAbinding protein 43 (TDP‐43) in amyotrophic lateral sclerosis (ALS) and frontotemporal dementia (FTD) [231, 232, 233].

PINK1/Parkindependent mitophagy provides the clearest genetic link between ubiquitin signaling and mitochondrial quality control in neurodegeneration. Pathogenic variants in Parkin RBR E3 ubiquitin protein ligase (PRKN) and PINK1 cause autosomal recessive early onset parkinsonism [234, 235]. Following mitochondrial damage, PINK1 accumulates on the outer mitochondrial membrane, phosphorylates ubiquitin, and activates Parkin, thereby amplifying mitochondrial ubiquitination [236, 237, 238]. These ubiquitin signals recruit the selective autophagy receptors OPTN and calciumbinding and coiled coil domain containing protein 2, while TANKbinding kinase 1 (TBK1)dependent receptor phosphorylation promotes engagement of the autophagic machinery and removal of damaged mitochondria [239, 240]. Failure of this pathway permits dysfunctional mitochondria to persist and increases oxidative stress and neuronal vulnerability.

The mitochondrial DUB USP30 counteracts Parkindependent ubiquitination and limits mitophagic signaling [241]. Genetic reduction or pharmacological inhibition of USP30 enhances mitochondrial ubiquitination and mitophagy in experimental systems. In Parkindeficient human neurons, USP30 inhibition increased mitophagic flux and reduced oxidative stress, suggesting that removal of this inhibitory checkpoint may partially compensate for impaired Parkin activity [242]. Nevertheless, this therapeutic concept remains preclinical, and sustained neuroprotection in patients has not been established.

Defective recognition and clearance of ubiquitinated cargo constitute a second disease‐associated axis. HDAC6 contributes to the handling of ubiquitinated TDP‐43, huntingtin, and α‐syn in experimental models of ALS, Huntington's disease, and PD [243, 244, 245, 246]. Although HDAC6 belongs to the HDAC family, its principal function in this context involves cytoplasmic cargo transport, aggregate sequestration, and autophagic clearance rather than chromatin regulation. Human genetics further supports the importance of this pathway: pathogenic ubiquilin‐2 (UBQLN2) variants cause Xlinked ALS, frequently accompanied by dementia and ubiquitinpositive inclusions, whereas diseaseassociated variants in SQSTM1 and OPTN and haploinsufficiency of TBK1 are linked to ALS and FTD [247, 248, 249, 250]. These abnormalities converge on defective ubiquitin recognition, cargo delivery, receptor activation, or autophagic clearance.

Dysregulated ubiquitindependent turnover of epigenetic enzymes may provide an additional link between proteostasis failure and neuronal transcriptional dysfunction. In ADrelated neuronal and transgenic mouse models, c‐Abldependent phosphorylation reduces the polyubiquitination and proteasomal degradation of HDAC2. The resulting HDAC2 accumulation increases transcriptional repression at synaptic gene promoters and contributes to impaired neuronal gene expression [251]. In a PD cellular model, SIRT1mediated deacetylation promotes TET2 ubiquitination and proteasomal degradation, thereby reducing TET2dependent hydroxymethylation and attenuating CDKN2Aassociated cell cycle arrest and neuronal injury [252]. These findings indicate that neurodegenerative stress can alter the ubiquitindependent stability of chromatin regulators. However, neither pathway has been established as a direct component of PINK1/Parkin signaling, and the SIRT1/TET2 degradation mechanism still requires validation in animal models and patients.

Beyond PINK1/Parkindependent mitophagy, neurodegenerative disease pathogenesis involves broader proteostasis failure, ER stress, and impaired UPS autophagy crosstalk. In ALS, precision medicine and biomarker guided therapeutic development are increasingly focused on protein homeostasis and genetic stratification [253], while in PD, ER stress and unfolded protein response signaling intersect with α‐syn aggregation, mitochondrial dysfunction, and impaired autophagic flux [254].

Collectively, neurodegenerative diseases reflect failures at several distinct stages of ubiquitindependent quality control. Impaired UPS activity promotes the accumulation of misfolded proteins; defective ubiquitin recognition and cargo transport favor persistent inclusions; disruption of the PINK1/Parkin/USP30 balance compromises mitochondrial surveillance; and altered stability of epigenetic enzymes may reinforce maladaptive neuronal transcriptional states. The genetic heterogeneity of PRKN, PINK1, UBQLN2, SQSTM1, OPTN, and TBK1associated disorders indicates that a single general proteostasis enhancing intervention is unlikely to be uniformly effective. Therapeutic strategies will instead require pathwayspecific and genotypeinformed approaches that distinguish defects in substrate ubiquitination, ubiquitin decoding, aggregate clearance, mitophagy, and secondary epigenetic dysregulation.

### 4.3 Inflammatory and Autoimmune Diseases: Defective Ubiquitin Editing and Loss of Immune Tolerance

Inflammatory and autoimmune diseases can arise when normally transient ubiquitin signals are not appropriately terminated or when immune pathways remain active in the absence of infection. The strongest causal evidence currently comes from monogenic autoinflammatory disorders and interferonopathies affecting NFκB regulation, linear ubiquitin signaling, nucleic acid sensing, and inflammasome activation. These disorders demonstrate that defects in ubiquitin generation, editing, or signal termination can convert protective immune responses into persistent cytokine production, tissue injury, immunodeficiency, and loss of immune tolerance.

Tumor necrosis factorαinduced protein 3 (TNFAIP3, also known as A20) haploinsufficiency provides direct evidence that defective ubiquitin editing can cause systemic inflammation. Heterozygous lossoffunction variants in TNFAIP3 cause an early onset autoinflammatory disorder characterized by recurrent fever, oral and genital ulceration, gastrointestinal inflammation, arthritis, and Behçet like manifestations [255]. Reduced A20 function prolongs receptorinduced inflammatory signaling and lowers the activation threshold of both innate and adaptive immune responses.

OTULINrelated autoinflammation provides a complementary example involving linear ubiquitin signaling. Biallelic hypomorphic variants in OTULIN impair the removal of M1linked ubiquitin chains and cause otulipenia, a severe systemic autoinflammatory disorder presenting in early life [256]. Conversely, inherited deficiencies of the LUBAC components HOIL‐1 or HOIP reduce linear ubiquitination and cause combinations of immunodeficiency, recurrent infection, autoinflammation, amylopectinosis, and lymphatic abnormalities [257, 258]. These disorders demonstrate that both excessive persistence and insufficient generation of linear ubiquitin signals can disrupt immune homeostasis.

Persistent nucleic acid sensing and inflammasome activation produce distinct inflammatory phenotypes. Activating variants in TMEM173 cause STINGassociated vasculopathy with onset in infancy (SAVI), which is characterized by constitutive type I interferon signaling, systemic inflammation, cutaneous vasculopathy, and progressive interstitial lung disease [259]. Although the initiating lesion is constitutive STING activation rather than a mutation in a ubiquitin enzyme, SAVI illustrates the pathological consequences of sustained signaling within a pathway whose assembly and termination are regulated by ubiquitination and deubiquitination. At the inflammasome level, regulation by ubiquitination and deubiquitination modifies the abundance and activation threshold of NLRP3 [260, 261]. More directly, activating NLRP3 variants cause cryopyrinassociated periodic syndromes, including familial cold autoinflammatory syndrome and Muckle Wells syndrome [262]. These disorders demonstrate that persistent interferon or inflammasome signaling is sufficient to drive recurrent fever, tissue inflammation, vasculopathy, hearing loss, and systemic injury.

Failure to distinguish endogenous from foreign nucleic acids provides another direct route to human inflammatory disease. ADAR1mediated A‐to‐I editing prevents endogenous double stranded RNA from activating melanoma differentiation‐associated protein 5 and Type I interferon signaling [263]. Pathogenic ADAR1 variants cause Aicardi Goutières syndrome, a monogenic interferonopathy associated with neurological inflammation, intracranial calcification, skin lesions, and persistent expression of interferon stimulated genes [264]. This human genetic evidence establishes defective self‐RNA tolerance as a cause of chronic interferon driven inflammation.

Human evidence directly linking altered ubiquitindependent stability of epigenetic enzymes to inflammatory disease remains more limited. In inflammatory bowel disease, increased FBXW7 expression in inflamed intestinal tissue has been associated with disease severity, whereas mechanistic studies indicate that FBXW7dependent EZH2 degradation in resident macrophages reduces H3K27Me3mediated repression of chemokine genes and promotes CCL2/CCL7dependent recruitment of inflammatory cells [76]. In idiopathic pulmonary fibrosis, p300 abundance and UCHL3/p300 colocalization are increased in patient lung epithelium, consistent with experimental evidence that UCHL3‐mediated deubiquitination and stabilization of p300 enhances chemokine transcription and profibrotic epithelial macrophage communication [147]. However, direct quantification of altered EZH2 or p300 ubiquitination in patient derived immune or epithelial cells remains uncommon, and most causal evidence is derived from cellular and animal models.

Additional epigenetic and epitranscriptomic pathways described earlier may modify inflammatory phenotypes but currently have weaker human causal support. The NAT10/RACK1 axis promotes pathogenic cycling T cell expansion, the SMURF2/ADAR1p110 axis may reinforce RNA editing capacity, and the RNF180/ALKBH5/SMARCA5 axis aggravates colonic inflammation and T helper 17/regulatory T cell imbalance in experimental models [101, 104, 186]. These pathways should therefore be interpreted as potential disease modifying mechanisms rather than established primary causes of defined human autoimmune diseases.

Collectively, human genetics establishes defective ubiquitin editing, abnormal linear ubiquitination, persistent nucleic acid sensing, and reduced inflammasome activation thresholds as direct causes of inflammatory disease. Patientassociated findings involving the FBXW7/EZH2 and UCHL3/p300 pathways extend this framework to ubiquitindependent regulation of epigenetic enzymes, although their causal mechanisms remain supported largely by experimental models. Distinguishing direct genetic causality from disease‐associated and preclinical evidence is essential when prioritizing ubiquitination/deubiquitination pathways for therapeutic intervention.

## 5 Therapeutic Strategies Targeting the Ubiquitin System

Therapeutic targeting of the ubiquitin system can eliminate disease driving proteins, expose substratespecific DUB dependencies, or disrupt pathological E3 substrate interactions. These strategies are especially relevant for transcription factors, chromatin regulators, RNAbinding proteins, signaling adaptors, and multiprotein complex components, whose noncatalytic functions often evade conventional active site inhibitors. Key approaches include targeted protein degradation (via PROTACs or molecular glues), selective DUB inhibition, direct modulation of disease relevant E3 ligases, and expanding the repertoire of E3 ligases usable for induced degradation [20, 27, 265].

These mechanistic distinctions also inform therapeutic strategy selection. Targeted protein degradation, DUB inhibition, and E3 ligase modulation are not necessarily interchangeable, because they differ in their effects on target abundance, protein function, and potentially multiple substrates or signaling pathways. Selecting an appropriate intervention therefore requires identifying the disease relevant function of the target and evaluating the consequences of perturbing its ubiquitindependent regulation.

### 5.1 PROTACs and Molecular Glues: Redirecting E3 Ligases Toward Pathogenic Proteins

PROTACs are heterobifunctional molecules comprising a targetbinding ligand, a linker, and an E3 ligase ligand. Formation of a productive target/PROTAC/E3 ternary complex promotes target ubiquitination and proteasomal degradation, with degradation efficiency and selectivity strongly influenced by ternary complex geometry and cooperativity [27, 266]. Molecular glues are generally smaller compounds that create or stabilize an interaction between an E3 ligase and a neosubstrate without requiring a bifunctional linker. Aryl sulfonamides, for example, recruit RBM39 to CRL4^DCAF15^ [267, 268]. By eliminating the entire target protein, these approaches can suppress catalytic and noncatalytic functions, although their activity depends on ternary complex geometry, target lysine accessibility, intracellular colocalization, and an intact UPS.

Targeted degradation has been extended to epigenetic and epitranscriptomic regulators. EED directed PROTACs induce EED degradation together with depletion of the associated PRC2 components EZH2 and SUZ12 [24]. The pVHL recruiting degrader KW0113 induces dose and timedependent degradation of DNMT1 in AML models, whereas WD6305 reduces METTL3 and METTL14 abundance, decreases m^6^A levels, and suppresses leukemia cell proliferation [25, 26]. These examples show that targeted degradation can disrupt DNA methylation, polycomb repression, and RNA methylation machinery rather than inhibit an individual catalytic site (Figure 5).

**FIGURE 5:**
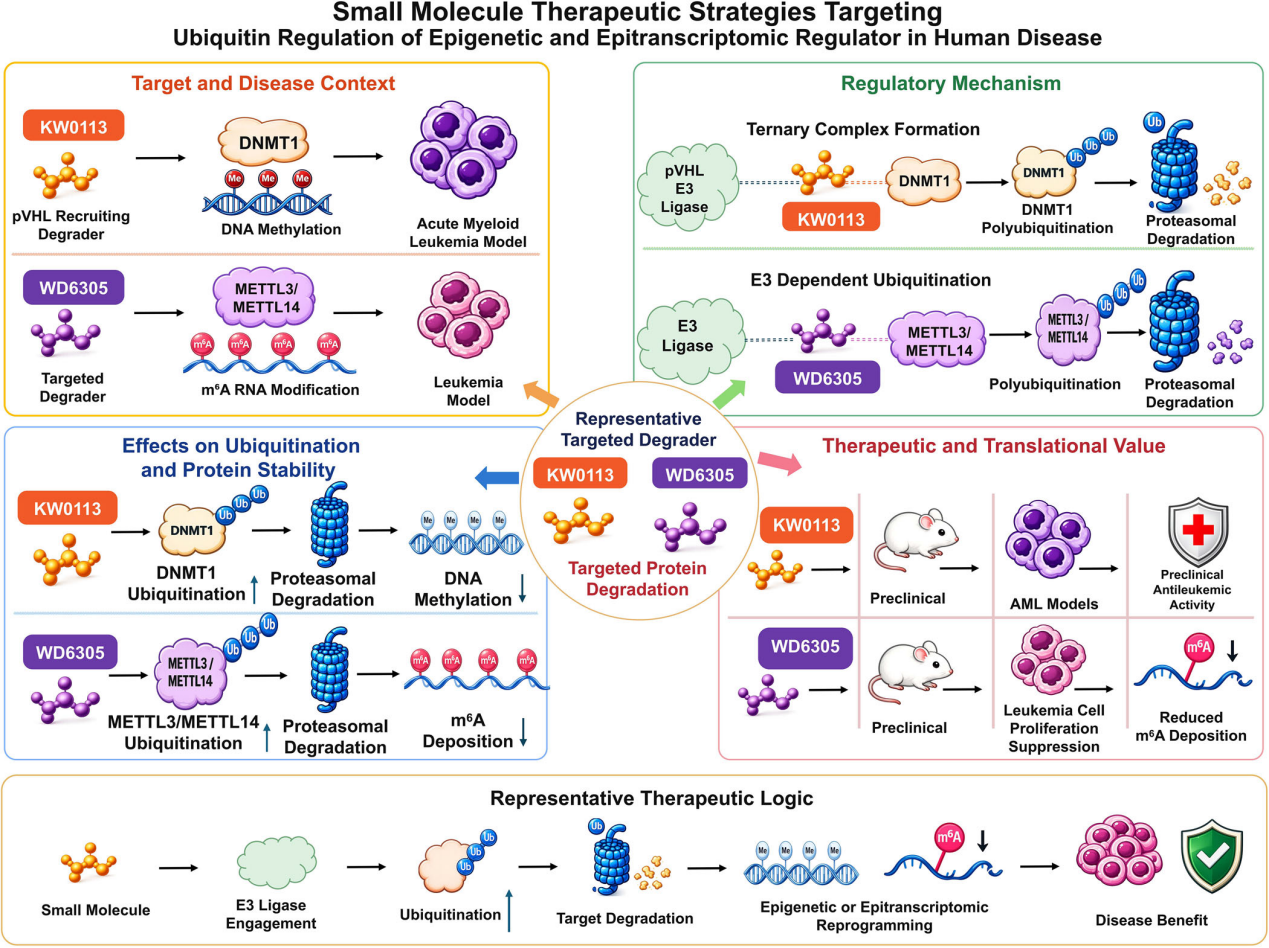
Targeted protein degradation of epigenetic and epitranscriptomic regulators. This schematic summarizes E3 ligasebased targeted protein degradation strategies for epigenetic and epitranscriptomic regulators. Representative degraders, KW0113 and WD6305, induce ubiquitination and proteasomal degradation of DNMT1 or the METTL3/METTL14 complex, altering DNA methylation or m^6^A levels and exerting therapeutic effects in leukemia models.

Chromatin remodeling dependencies provide another application. A947 preferentially degrades SMARCA2 and suppresses tumor growth in SMARCA4 mutant models that depend on the remaining SWI/SNF ATPase [269]. Its selectivity is driven primarily by productive ternary complex formation, illustrating how induced degradation can distinguish between closely related paralogous proteins.

Targeted degradation may also be applicable to immunoregulatory epigenetic proteins. Bobcat339 has been reported to enhance the association of TET3 with pVHL, promote TET3 degradation in macrophages, and reduce disease progression in mouse models of endometriosis [270]. However, direct structural evidence demonstrating simultaneous engagement of Bobcat339, TET3, and pVHL remains unavailable. It should therefore be described as a reported pVHLdependent TET3 degrader rather than a fully validated molecular glue.

These programs provide proof of concept for the degradation of epigenetic, epitranscriptomic, and chromatin remodeling proteins but remain preclinical. Their translation will require confirmation of disease relevant target dependency, degradation depth and duration, and activity in appropriate cellular and animal models.

### 5.2 DUB Inhibitors: Selectively Disrupting Substrate Deubiquitination

Pharmacological inhibition of DUBs can expose diseasespecific substrate dependencies by preventing the removal or remodeling of ubiquitin modifications. Its therapeutic effect is context dependent because inhibition of a DUB that stabilizes an oncogenic protein may be beneficial, whereas inhibition of a DUB that preserves genome stability or limits inflammatory signaling may be detrimental. Target selection therefore requires definition of the relevant substrate dependency and the expected direction of pathway regulation in disease relevant cells [30, 32].

USP7 is one of the most extensively investigated DUB targets for pharmacological inhibition. The selective inhibitors FT671 and FT827 bind a pocket adjacent to the USP7 catalytic center. In tumor protein p53 gene (TP53) WT experimental models, FT671 destabilized MDM2, increased p53 and CDK inhibitor 1A (p21) abundance, and inhibited xenograft growth [271]. These findings provide structural and preclinical evidence that selective USP7 inhibition can restore p53dependent signaling, although clinical efficacy has not been established and the response remains dependent on TP53 status and downstream pathway competence.

USP1 inhibition represents a mechanistically defined strategy based on DNA repair dependency. The USP1/UAF1 complex regulates FANCD2, PCNA, and cellular responses to replication stress. The selective inhibitor KSQ‐4279 enhanced the activity of poly (ADPribose) polymerase (PARP) inhibitors and produced tumor regression in BRCA mutant or homologous recombinationdeficient preclinical models, including models with acquired resistance to PARP inhibition [272]. USP1 inhibition should therefore be regarded as a biomarkerguided strategy for tumors with defined DNA repair defects rather than a broadly applicable anticancer treatment.

Inhibition of proteasomeassociated DUB activity represents a less selective approach. VLX1570 disrupts proteasomal deubiquitination, leading to accumulation of ubiquitinated proteins and proteotoxic stress. In a Phase I study involving 14 patients with relapsed or refractory multiple myeloma, two patients treated at the highest administered dose developed rapidly progressive respiratory insufficiency that culminated in death, leading to termination of the study [273]. Although a contribution from the formulation could not be excluded, the limited exposure and early termination prevented reliable assessment of antitumor efficacy. This experience illustrates the narrow therapeutic window associated with broad disruption of proteasomal protein processing.

Together, these studies distinguish selective inhibition of substrate defined DUB dependencies from broad induction of proteotoxic stress. Further development should prioritize selective target engagement, clearly defined substrate or pathway dependence, and therapeutic windows demonstrated in disease relevant models.

### 5.3 Direct Modulation of E3 Ligases and Expansion of the E3 Recruiter Space

E3 ligases can be therapeutically targeted by blocking pathological substrate interactions or redirecting them to diseasepromoting substrates. This section focuses on direct modulation of disease‐relevant E3 ligases and emerging recruiters beyond cereblon (CRBN) and pVHL.

MDM2 exemplifies direct E3 ligase modulation. Nutlins bind MDM2's p53 recognition pocket, blocking the MDM2/p53 interaction and thereby reducing p53 ubiquitination to restore p53dependent transcription and cell cycle arrest in TP53 WT models [274]. These compounds inhibit substrate recognition rather than the catalytic RING domain. However, in the Phase III MIRROS trial, idasanutlin combined with cytarabine did not improve overall survival compared with placebo plus cytarabine in the TP53 WT intention to treat population, despite a higher overall response rate [275]. These results indicate that MDM2 inhibition and p53 stabilization alone are insufficient to ensure clinical benefit and require functional downstream p53 signaling and appropriate patient selection.

IAPs provide a distinct model of E3 directed therapy. cIAP1 and cIAP2 are E3 ligases that regulate RIPK1, NFκB signaling, and cell survival. SMAC mimetics induce cIAP autoubiquitination and proteasomal degradation, triggering TNFdependent apoptosis in susceptible tumor cells [276]. Unlike MDM2 antagonists, this strategy exploits the catalytic activity of an E3 ligase to eliminate the ligase itself. Its efficacy nevertheless depends on TNF production and intact downstream cell death machinery.

Emerging covalent recruiters expand the E3 ligase repertoire available for induced degradation. Nimbolide covalently engages Cys8 of RNF114 and has been incorporated into bifunctional degraders that redirect RNF114 toward new substrates [277]. However, nimbolide can also interfere with recognition of endogenous RNF114 substrates and stabilize p21. Intended target degradation must therefore be evaluated together with perturbation of the native substrate network of the recruited ligase.

Chemoproteomic studies have identified additional covalent recruiters for DDB1 and CUL4‐associated factor 16 (DCAF16), DCAF11, and FEM1B. These ligases have supported experimental degradation of nuclear proteins, androgen receptors, BRD4, and other targets [278, 279, 280]. Although these findings expand the range of recruitable E3 ligases, the resulting degraders remain predominantly preclinical, and their disease relevant expression, degradation selectivity, and effects on endogenous substrates require further characterization.

Together, substrate recognition antagonism, ligase auto degradation, and induced E3 recruitment represent mechanistically distinct strategies for therapeutic E3 ligase modulation. Their translation will require preservation of essential endogenous ligase functions, selective degradation of the intended target, and validation of the relevant disease dependency.

### 5.4 Clinical Translation: Efficacy, Selectivity, Toxicity, and Resistance

Molecular glue drugs provided the earliest clinical validation of pharmacologically induced protein degradation. CRBN‐dependent degradation of IKZF1 and IKZF3 explains a central component of lenalidomide activity in multiple myeloma and demonstrates that an approved small molecule can therapeutically remodel E3 substrate recognition [22]. More recently, heterobifunctional protein degradation demonstrated clinical efficacy in the Phase III VERITAC‐2 trial. Vepdegestrant significantly prolonged progressionfree survival (PFS) relative to fulvestrant in patients with estrogen receptor 1 (ESR1) mutated, estrogen receptorpositive/HER2negative advanced breast cancer, but not in the overall trial population. In the ESR1mutated subgroup, median PFS was 5.0 months with vepdegestrant and 2.1 months with fulvestrant, corresponding to a hazard ratio of 0.58 (95% confidence interval, 0.43–0.78) [281]. The statistically significant PFS benefit observed in the ESR1mutated population further emphasizes the importance of molecularly defined target dependency and biomarker guided patient selection.

Clinical development has also extended to chromatin regulators. In the Phase I study, the heterobifunctional bromodomain containing protein 9 (BRD9) degrader FHD‐609 produced extensive BRD9 degradation in tumor tissue from patients with advanced synovial sarcoma or SMARCB1deficient tumors. However, singleagent antitumor activity was limited, and QTc prolongation was observed, demonstrating that deep target degradation does not necessarily translate into a wide therapeutic window or substantial clinical efficacy [282].

Targeted degradation has also entered clinical development outside oncology. In a Phase I study, the IRAK4 degrader KT‐474 produced substantial IRAK4 loss in blood and skin, inhibited downstream inflammatory signaling, and showed preliminary activity in small cohorts of patients with hidradenitis suppurativa or atopic dermatitis [283]. The limited cohort size, openlabel patient component, and short treatment duration precluded definitive assessment of efficacy. The study therefore established pharmacodynamic target engagement but did not demonstrate durable clinical benefit.

Although most clinical stage degraders target oncology, ubiquitin‐based degradation strategies are being explored in nonmalignant diseases. For example, PROTAC technology and precise modulation of cGAS/STING signaling have been proposed for kidney diseases, highlighting opportunities for tissue selective degraders and biomarker guided patient selection beyond cancer [284].

DUB inhibitors remain less clinically mature. The VLX1570 experience illustrates the narrow therapeutic window associated with broad disruption of proteasomal deubiquitination [273]. These findings underscore the distinction between substrate defined DUB dependencies and nonspecific proteotoxic stress.

Clinical selectivity is determined by the combined effects of target dependency, productive target E3 engagement, intracellular colocalization, E3 competence, degradation kinetics, and tissue exposure. A degrader may therefore bind its target without producing sufficient degradation or may achieve different degradation depths across tissues. Molecular glues and E3 recruiters can also induce unexpected neosubstrate or off target degradation. Proteome wide profiling and tissuespecific pharmacodynamic measurements are therefore required to distinguish intended target loss from broader perturbation of the proteome.

Toxicity may result from excessive removal of an essential target, loss of noncatalytic functions in normal tissues, unintended neosubstrate degradation, disruption of immune homeostasis, or broad proteotoxic stress. These liabilities cannot be predicted adequately from binarybinding affinity or disease cell potency alone and require evaluation of normal tissue dependencies, degradation depth and duration, reversibility, and recovery after treatment withdrawal.

Resistance may involve either the target protein or the degradation machinery. Cancer cells resistant to BET directed PROTACs have acquired genomic alterations in components of CRBN or pVHLassociated ubiquitin ligase complexes [285]. Functional studies similarly indicate that resistance frequently converges on impairment of the ubiquitin transfer pathway engaged by the degrader [286]. The composition and plasticity of cullin/RING ligase complexes also influence sensitivity to ligandinduced degradation [287]. Additional mechanisms may include mutation of the target‐binding interface, disruption of degraderinduced protein–protein interactions (PPIs), reduced E3 expression, defective neddylation or ubiquitin transfer, altered intracellular drug exposure, and activation of compensatory survival pathways.

Clinical translation therefore requires integrated biomarkers that assess target dependency; E3 ligase abundance and functional competence; target E3 colocalization; degradation depth and duration; downstream pathway suppression; and resistanceassociated alterations. For DUB inhibitors, biomarkers must also confirm selective DUB engagement and identify substrate networks driving therapeutic responses. Combination strategies should be guided by experimentally validated dependencies, not added empirically.

Overall, clinical experience demonstrates that target degradation alone is not a sufficient measure of therapeutic success. Durable benefit requires a disease relevant target dependency, adequate and selective degradation in the appropriate tissue, an acceptable therapeutic window, and biomarker guided selection of patients most likely to respond.

## 6 Challenges and Future Perspectives

Despite rapid progress in targeted protein degradation and pharmacological modulation of E3 ligases and DUBs, the central challenge is no longer whether the ubiquitin system can be therapeutically targeted, but how to match a genuine disease dependency with the right ubiquitin regulator, cellular compartment, and molecularly defined patient population. Meeting this challenge requires causal validation of ubiquitin regulatory events; functional biomarkers of target dependency and regulator competence; and expansion of the recruitable E3 ligase repertoire.

A major limitation is that many reported ubiquitination events remain descriptive rather than causal. Detection of a ubiquitin remnantmodified lysine identifies a candidate ubiquitination site but does not by itself establish the responsible E3 ligases or DUBs, ubiquitin chain architecture, modification stoichiometry, or functional consequence. Ubiquitin remnant proteomics can identify large numbers of candidate sites but cannot directly assign them to specific ubiquitin regulators or distinguish degradative from nondegradative events [288]. Likewise, DUB abundance does not necessarily reflect catalytic activity. Ubiquitinbased activity probes can assess active site competence, inhibitor occupancy, and selectivity, but cannot alone establish physiological substrate engagement [289]. Emerging approaches, including multiplex clustered regularly interspaced short palindromic repeats (CRISPR) screening and proximitybased identification of ubiquitin sites, can connect E3 ligases to candidate substrates and, in selected settings, map degraderinduced ubiquitination sites at amino acid resolution [290, 291]. Candidate regulatory events should therefore be validated through genetic perturbation, site directed mutagenesis, rescue experiments, quantitative protein turnover measurements, and analysis of downstream biological effects.

The same applies to therapeutic biomarkers. Target abundance alone rarely predicts response: highly expressed proteins are not necessarily disease dependencies, and low abundance proteins may be essential in genetically defined populations. Predictive biomarkers must confirm target dependence and functional competence of required ubiquitin system components, including E3 ligases, DUBs, E2 enzymes, the neddylation machinery, and the proteasome. Pharmacodynamic biomarkers must demonstrate target loss or altered ubiquitination in disease relevant tissue and link that effect to suppression of the pathological pathway. Bloodbased measurements cannot be assumed to reflect target modulation in tumors, skin, brain, or other affected compartments.

For cullin/RING ligases, abundance is an incomplete biomarker because expression does not establish complex assembly or neural precursor cell expressed, developmentally downregulated 8 (NEDD8)dependent catalytic competence. Conformationspecific profiling of NEDD8modified cullins has shown that active CUL1/CUL4 ligase repertoires differ among cell types and cellular states and can influence targeted degradation efficiency [292]. Functional assessment of cullin/RING ligase activity may therefore be more informative than transcript or total protein abundance when selecting a cullinbased recruiter or predicting response.

Expanding the E3 ligase repertoire remains a major opportunity to extend targeted degradation to previously inaccessible proteins, tissues, and cellular compartments. An integrative analysis prioritized 76 candidate E3 ligases for PROTAC development based on confidence scores, ligandability, expression patterns, anddata on PPIs, while also characterizing structural availability, functional essentiality, and subcellular localization [28]. The human E3‐ome compendium subsequently defined 672 high confidence E3 ligases and mapped their molecular classification, tissue and cell type distribution, subcellular context, and diseaseassociated variation [293]. These resources substantially expand the theoretical E3 landscape, but they should not be interpreted as collections of biochemically validated or chemically recruitable ligases. Most E3 ligases still lack validated recruiters and have not been shown to support efficient degradation of therapeutic targets.

The value of a new recruiter should be determined by biological compatibility rather than simply by the number of additional E3 ligases it makes available. Individual ligases differ in tissue distribution, subcellular localization, endogenous substrates, E2 partnerships, catalytic regulation, and compatibility with target surfaces. DCAF16 illustrates how nuclear localization can support degradation of nuclear proteins [278], whereas PROTACs recruiting DDB1 and CUL4associated factor 1 (DCAF1) have supported degradation of nuclear BRD9 together with cytosolic and membraneassociated kinases and have retained activity in selected models with intrinsic pVHL deficiency or acquired resistance to CRBNbased degraders [294]. Highresolution structures of DCAF1/PROTAC/WDR5 complexes further showed that DCAF1 loop plasticity, linkerdependent interactions, and target orientation influence ternary complex assembly and substrate presentation [295]. These findings reinforce the need to match each ligase to the localization and geometry of the intended target rather than treating E3 ligases as interchangeable ubiquitin sources.

New recruiters may also perturb the physiological substrate network of the engaged ligase. Nimbolide covalently engages Cys8 of RNF114 and enables RNF114 recruitment toward new targets, but it can also disrupt recognition of endogenous RNF114 substrates and stabilize p21 [277]. Candidate recruiters should therefore be evaluated for ligase activity, genetic essentiality, endogenous substrate perturbation, and proteome wide consequences in disease relevant and vulnerable normal cells. Ligases involved in immune homeostasis, development, genome maintenance, or mitochondrial quality control may require caution during prolonged treatment.

Future development should integrate chemical proteomics, structural biology, functional genomics, quantitative protein turnover assays, and disease relevant models. CRISPR screens can identify E3 ligases, E2 enzymes, cullins, neddylation components, proteasome subunits, and DUBs essential for target degradation, or for response to DUB inhibitors. Single cell and tissue atlases can determine whether these components are expressed in pathological cell populations and whether they are relatively depleted from vulnerable normal tissues; however, activity based or functional assays remain necessary to establish catalytic competence. Patient derived models and longitudinal clinical samples can then determine whether target modulation produces the expected pathway response and whether treatment selects alterations in the target, recruited ubiquitin regulator, or downstream signaling network.

Ultimately, the field must shift from descriptive ubiquitination maps and empirical degrader screening to contextspecific manipulation of protein fate. Success requires evidence that: the target is a genuine disease dependency; the selected E3 ligases or DUBs are functionally active in relevant tissues; and intended molecular and biological effects occur at clinically achievable exposures. Expanding the ubiquitin toolkit broadens the scope of targetable proteins but durable clinical benefit depends on matching the right target, ubiquitin regulator, cellular compartment, and patient population using functional biomarkers.

## 7 Conclusion

Ubiquitination and deubiquitination form a reversible signaling system that links proteostasis to epigenetic and epitranscriptomic regulation, genome maintenance, immune signaling, RNA metabolism, cell fate control, and organelle quality control. The biological outcome depends not only on whether a substrate is ubiquitinated, but also on its identity, ubiquitin chain topology and architecture, subcellular location, and recognition by ubiquitinbinding proteins. E3 ligases generate these signals, DUBs edit or terminate them, and ubiquitin readers translate them into degradative or nondegradative outcomes, shifting the paradigm beyond ubiquitin's traditional role as a mere proteasomal degradation tag.

Dysregulation drives disease through both altered protein abundance and distorted signaling duration, localization, chromatin recruitment, and molecular interactions. In cancer, ubiquitination/deubiquitination imbalance stabilizes oncogenic programs, impairs tumor suppression, and promotes cellular plasticity and therapy resistance. In neurodegeneration, defective proteasomal clearance, ubiquitindependent cargo handling, and mitochondrial quality control cause aggregate accumulation and neuronal injury. In inflammatory and autoimmune disorders, faulty ubiquitin editing sustains hyperactive receptor proximal, interferon, or inflammasome signaling, or disrupts tolerance to self‐nucleic acids. Collectively, these contexts show that ubiquitin dysregulation has highly contextspecific consequences, not predictable from protein abundance alone.

Therapeutically, PROTACs, molecular glues, DUB inhibitors, and direct E3 modulators demonstrate that protein fate can be pharmacologically reprogrammed. Yet clinical experience shows that target degradation or pathway engagement alone is insufficient for durable benefit. Sustained responses require validated disease dependency; functional E3 ligases or DUBs in relevant tissues; selective pathway modulation; an acceptable therapeutic window; and biomarker guided patient selection. Future progress hinges on replacing descriptive ubiquitination maps with causally validated, context resolved regulatory networks, and integrating structural biology, quantitative proteomics, functional genetics, and disease relevant models. This integrated approach will enable more precise use of the ubiquitin system as both a mechanistic framework and a therapeutic platform. The central insight of this synthesis is that ubiquitindependent regulation must be evaluated through both protein abundance and functional state. Connecting these molecular consequences with disease‐specific dependencies provides a basis for choosing and evaluating therapeutic interventions.

## Author Contributions

W.L. and J.Z. were responsible for drafting the initial manuscript. D.G., T.Y., and T.Z. contributed by offering editorial support, writing guidance, and valuable suggestions. The final version of the manuscript was reviewed and approved by T.Z. and Z.Y. prior to submission. All authors played a role in the conception and submission of the manuscript.

## Ethics Statement

The authors have nothing to report.

## Conflicts of Interest

The authors declare no conflicts of interest.

## Data Availability

All data supporting this review's findings come from the publicly available literature cited in the manuscript; no new primary data were generated.

## References

[1] C. H. Waddington , “Canalization of Development and the Inheritance of Acquired Characters,” Nature 150 (1942): 563–565.

[2] C. D. Allis and T. Jenuwein , “The Molecular Hallmarks of Epigenetic Control,” Nature Reviews Genetics 17 (2016): 487–500. 10.1038/nrg.2016.59 27346641

[3] A. Bird , “Perceptions of Epigenetics,” Nature 447 (2007): 396–398. 10.1038/nature05913 17522671

[4] M. A. Dawson and T. Kouzarides , “Cancer Epigenetics: From Mechanism to Therapy,” Cell 150 (2012): 12–27. 10.1016/j.cell.2012.06.013 22770212

[5] J. J. Day and J. D. Sweatt , “Epigenetic Mechanisms in Cognition,” Neuron 70 (2011): 813–829. 10.1016/j.neuron.2011.05.019 PMC3118503 21658577

[6] Z. Du , J. Song , Y. Wang , et al., “DNMT1 Stability Is Regulated by Proteins Coordinating Deubiquitination and Acetylation‐Driven Ubiquitination,” Science Signaling 3 (2010): ra80. 10.1126/scisignal.2001462 PMC3116231 21045206

[7] M. H. Glickman and A. Ciechanover , “The Ubiquitin‐Proteasome Proteolytic Pathway: Destruction for the Sake of Construction,” Physiological Reviews 82 (2002): 373–428. 10.1152/physrev.00027.2001 11917093

[8] A. Hershko and A. Ciechanover , “The Ubiquitin System,” Annual Review of Biochemistry 67 (1998): 425–479. 10.1146/annurev.biochem.67.1.425 9759494

[9] D. Finley , “Recognition and Processing of Ubiquitin‐Protein Conjugates by the Proteasome,” Annual Review of Biochemistry 78 (2009): 477–513. 10.1146/annurev.biochem.78.081507.101607 PMC3431160 19489727

[10] M. J. Clague , S. Urbé , and D. Komander , “Breaking the Chains: Deubiquitylating Enzyme Specificity Begets Function,” Nature Reviews Molecular Cell Biology 20 (2019): 338–352. 10.1038/s41580-019-0099-1 30733604

[11] D. Komander and M. Rape , “The Ubiquitin Code,” Annual Review of Biochemistry 81 (2012): 203–229. 10.1146/annurev-biochem-060310-170328 22524316

[12] K. N. Swatek and D. Komander , “Ubiquitin Modifications,” Cell Research 26 (2016): 399–422. 10.1038/cr.2016.39 PMC4822133 27012465

[13] Y. Liao , W. Zhang , Y. Liu , et al., “The Role of Ubiquitination in Health and Disease,” MedComm 5 (2024): e736. 10.1002/mco2.736 PMC11424685 39329019

[14] T. Johansen and T. Lamark , “Selective Autophagy Mediated by Autophagic Adapter Proteins,” Autophagy 7 (2011): 279–296. 10.4161/auto.7.3.14487 PMC3060413 21189453

[15] S. Pankiv , T. H. Clausen , T. Lamark , et al., “p62/SQSTM1 Binds Directly to Atg8/LC3 to Facilitate Degradation of Ubiquitinated Protein Aggregates by Autophagy,” Journal of Biological Chemistry 282 (2007): 24131–24145. 10.1074/jbc.M702824200 17580304

[16] T. Nakagawa , L. Lv , M. Nakagawa , et al., “CRL4^VprBP^ E3 Ligase Promotes Monoubiquitylation and Chromatin Binding of TET Dioxygenases,” Molecular Cell 57 (2015): 247–260. 10.1016/j.molcel.2014.12.002 PMC4304937 25557551

[17] G. Kuser‐Abali , Y. Zhang , P. Szeto , et al., “UHRF1/UBE2L6/UBR4‐Mediated Ubiquitination Regulates EZH2 Abundance and Thereby Melanocytic Differentiation Phenotypes in Melanoma,” Oncogene 42 (2023): 1360–1373. 10.1038/s41388-023-02631-8 PMC10121471 36906655

[18] J. E. Lee , C. M. Park , and J. H. Kim , “USP7 Deubiquitinates and Stabilizes EZH2 in Prostate Cancer Cells,” Genetics and Molecular Biology 43 (2020): e20190338. 10.1590/1678-4685-GMB-2019-0338 PMC7252518 32453339

[19] H. L. Sun , A. C. Zhu , Y. Gao , et al., “Stabilization of ERK‐Phosphorylated METTL3 by USP5 Increases m^6^A Methylation,” Molecular Cell 80 (2020): 633–647.e7. 10.1016/j.molcel.2020.10.026 PMC7720844 33217317

[20] K. M. Sakamoto , K. B. Kim , A. Kumagai , et al., “PROTACs: Chimeric Molecules That Target Proteins to the Skp1‐Cullin‐F Box Complex for Ubiquitination and Degradation,” Proceedings of the National Academy of Sciences of the United States of America 98 (2001): 8554–8559. 10.1073/pnas.141230798 PMC37474 11438690

[21] A. C. Lai and C. M. Crews , “Induced Protein Degradation: An Emerging Drug Discovery Paradigm,” Nature Reviews Drug Discovery 16 (2017): 101–114. 10.1038/nrd.2016.211 PMC5684876 27885283

[22] J. Krönke , N. D. Udeshi , A. Narla , et al., “Lenalidomide Causes Selective Degradation of IKZF1 and IKZF3 in Multiple Myeloma Cells,” Science 343 (2014): 301–305. 10.1126/science.1244851 PMC4077049 24292625

[23] G. Fan , S. Chen , Q. Zhang , et al., “Proteolysis‐Targeting Chimera (PROTAC): Current Applications and Future Directions,” MedComm 6 (2025): e70401. 10.1002/mco2.70401 PMC12495453 41049269

[24] J. H. Hsu , T. Rasmusson , J. Robinson , et al., “EED‐Targeted PROTACs Degrade EED, EZH2, and SUZ12 in the PRC2 Complex,” Cell Chemical Biology 27 (2020): 41–46.e17. 10.1016/j.chembiol.2019.11.004 31786184

[25] H. Wang , Z. Wang , L. Hu , et al., “Discovery of KW0113 as a First and Effective PROTAC Degrader of DNMT1 Protein,” Chemmedchem 19 (2024): e202400467. 10.1002/cmdc.202400467 39302251

[26] W. Du , Y. Huang , X. Chen , et al., “Discovery of a PROTAC Degrader for METTL3‐METTL14 Complex,” Cell Chemical Biology 31 (2024): 177–183.e17. 10.1016/j.chembiol.2023.12.009 38194973

[27] M. Békés , D. R. Langley , and C. M. Crews , “PROTAC Targeted Protein Degraders: The Past Is Prologue,” Nature Reviews Drug Discovery 21 (2022): 181–200. 10.1038/s41573-021-00371-6 PMC8765495 35042991

[28] Y. Liu , J. Yang , T. Wang , et al., “Expanding PROTACtable Genome Universe of E3 Ligases,” Nature Communications 14 (2023): 6509. 10.1038/s41467-023-42233-2 PMC10579327 37845222

[29] C. Nieto‐Jiménez , E. C. Morafraile , C. Alonso‐Moreno , et al., “Clinical Considerations for the Design of PROTACs in Cancer,” Molecular Cancer 21 (2022): 67. 10.1186/s12943-022-01535-7 PMC8900451 35249548

[30] J. A. Harrigan , X. Jacq , N. M. Martin , et al., “Deubiquitylating Enzymes and Drug Discovery: Emerging Opportunities,” Nature Reviews Drug Discovery 17 (2018): 57–78. 10.1038/nrd.2017.152 PMC7097658 28959952

[31] D. Senft , J. Qi , and Z. A. Ronai , “Ubiquitin Ligases in Oncogenic Transformation and Cancer Therapy,” Nature Reviews Cancer 18 (2018): 69–88. 10.1038/nrc.2017.105 PMC6054770 29242641

[32] Y. Xian , J. Ye , Y. Tang , et al., “Deubiquitinases as Novel Therapeutic Targets for Diseases,” MedComm 5 (2024): e70036. 10.1002/mco2.70036 PMC11645450 39678489

[33] R. J. Deshaies and C. A. P. Joazeiro , “RING Domain E3 Ubiquitin Ligases,” Annual Review of Biochemistry 78 (2009): 399–434. 10.1146/annurev.biochem.78.101807.093809 19489725

[34] L. Buetow and D. T. Huang , “Structural Insights Into the Catalysis and Regulation of E3 Ubiquitin Ligases,” Nature Reviews Molecular Cell Biology 17 (2016): 626–642. 10.1038/nrm.2016.91 PMC6211636 27485899

[35] D. M. Wenzel , A. Lissounov , P. S. Brzovic , et al., “UBCH7 Reactivity Profile Reveals Parkin and HHARI to Be RING/HECT Hybrids,” Nature 474 (2011): 105–108. 10.1038/nature09966 PMC3444301 21532592

[36] R. Yau and M. Rape , “The Increasing Complexity of the Ubiquitin Code,” Nature Cell Biology 18 (2016): 579–586. 10.1038/ncb3358 27230526

[37] A. Williamson , A. Werner , and M. Rape , “The Colossus of Ubiquitylation: Decrypting a Cellular Code,” Molecular Cell 49 (2013): 591–600. 10.1016/j.molcel.2013.01.028 PMC3589588 23438855

[38] S. Elsasser , D. Chandler‐Militello , B. Müller , et al., “Rad23 and Rpn10 Serve as Alternative Ubiquitin Receptors for the Proteasome,” Journal of Biological Chemistry 279 (2004): 26817–26822. 10.1074/jbc.M404020200 15117949

[39] T. Wu , Y. Merbl , Y. Huo , et al., “UBE2S Drives Elongation of K11‐Linked Ubiquitin Chains by the Anaphase‐Promoting Complex,” Proceedings of the National Academy of Sciences of the United States of America 107 (2010): 1355–1360. 10.1073/pnas.0912802107 PMC2802591 20080579

[40] I. Dikic , S. Wakatsuki , and K. J. Walters , “Ubiquitin‐Binding Domains—From Structures to Functions,” Nature Reviews Molecular Cell Biology 10 (2009): 659–671. 10.1038/nrm2767 PMC7359374 19773779

[41] C. Kraft , M. Peter , and K. Hofmann , “Selective Autophagy: Ubiquitin‐Mediated Recognition and Beyond,” Nature Cell Biology 12 (2010): 836–841. 10.1038/ncb0910-836 20811356

[42] F. Ohtake , H. Tsuchiya , Y. Saeki , et al., “K63 Ubiquitylation Triggers Proteasomal Degradation by Seeding Branched Ubiquitin Chains,” Proceedings of the National Academy of Sciences of the United States of America 115 (2018): E1401–E1408. 10.1073/pnas.1716673115 PMC5816176 29378950

[43] F. Tokunaga , S. Sakata , Y. Saeki , et al., “Involvement of Linear Polyubiquitylation of NEMO in NFκB Activation,” Nature Cell Biology 11 (2009): 123–132. 10.1038/ncb1821 19136968

[44] M. Gatti , S. Pinato , A. Maiolica , et al., “RNF168 Promotes Noncanonical K27 Ubiquitination to Signal DNA Damage,” Cell Reports 10 (2015): 226–238. 10.1016/j.celrep.2014.12.021 25578731

[45] H. J. Meyer and M. Rape , “Enhanced Protein Degradation by Branched Ubiquitin Chains,” Cell 157 (2014): 910–921. 10.1016/j.cell.2014.03.037 PMC4028144 24813613

[46] T. E. T. Mevissen and D. Komander , “Mechanisms of Deubiquitinase Specificity and Regulation,” Annual Review of Biochemistry 86 (2017): 159–192. 10.1146/annurev-biochem-061516-044916 28498721

[47] D. Kwasna , S. A. Abdul Rehman , J. Natarajan , et al., “Discovery and Characterization of ZUFSP/ZUP1, a Distinct Deubiquitinase Class Important for Genome Stability,” Molecular Cell 70 (2018): 150–164.e6. 10.1016/j.molcel.2018.02.023 PMC5896202 29576527

[48] J. C. Scheuermann , A. G. de Ayala Alonso , K. Oktaba , et al., “Histone H2A Deubiquitinase Activity of the Polycomb Repressive Complex PR‐DUB,” Nature 465 (2010): 243–247. 10.1038/nature08966 PMC3182123 20436459

[49] L. Feng , J. Wang , and J. Chen , “The Lys63‐Specific Deubiquitinating Enzyme BRCC36 Is Regulated by Two Scaffold Proteins Localizing in Different Subcellular Compartments,” Journal of Biological Chemistry 285 (2010): 30982–30988. 10.1074/jbc.M110.135392 PMC2945589 20656690

[50] S. A. Abdul Rehman , Y. A. Kristariyanto , S. Y. Choi , et al., “MINDY‐1 Is a Member of an Evolutionarily Conserved and Structurally Distinct New Family of Deubiquitinating Enzymes,” Molecular Cell 63 (2016): 146–155. 10.1016/j.molcel.2016.05.009 PMC4942677 27292798

[51] L. L. Chen , M. D. Smith , L. Lv , et al., “USP15 Suppresses Tumor Immunity via Deubiquitylation and Inactivation of TET2,” Science Advances 6 (2020): eabc9730. 10.1126/sciadv.abc9730 PMC7500937 32948596

[52] N. A. Snyder and G. M. Silva , “Deubiquitinating Enzymes (DUBs): Regulation, Homeostasis, and Oxidative Stress Response,” Journal of Biological Chemistry 297 (2021): 101077. 10.1016/j.jbc.2021.101077 PMC8424594 34391779

[53] X. Zhao , S. Lv , N. Li , et al., “YTHDF2 Protein Stabilization by the Deubiquitinase OTUB1 Promotes Prostate Cancer Cell Proliferation via PRSS8 mRNA Degradation,” Journal of Biological Chemistry 300 (2024): 107152. 10.1016/j.jbc.2024.107152 PMC11002313 38462165

[54] K. Husnjak , S. Elsasser , N. Zhang , et al., “Proteasome Subunit Rpn13 Is a Novel Ubiquitin Receptor,” Nature 453 (2008): 481–488. 10.1038/nature06926 PMC2839886 18497817

[55] B. Sobhian , G. Shao , D. R. Lilli , et al., “RAP80 Targets BRCA1 to Specific Ubiquitin Structures at DNA Damage Sites,” Science 316 (2007): 1198–1202. 10.1126/science.1139516 PMC2706583 17525341

[56] Y. Sato , A. Yoshikawa , H. Mimura , et al., “Structural Basis for Specific Recognition of Lys63‐Linked Polyubiquitin Chains by Tandem UIMs of RAP80,” EMBO Journal 28 (2009): 2461–2468. 10.1038/emboj.2009.160 PMC2735169 19536136

[57] S. Rahighi , F. Ikeda , M. Kawasaki , et al., “Specific Recognition of Linear Ubiquitin Chains by NEMO Is Important for NFκB Activation,” Cell 136 (2009): 1098–1109. 10.1016/j.cell.2009.03.007 19303852

[58] T. Gu , D. Hao , J. Woo , et al., “The Disordered N‐Terminal Domain of DNMT3A Recognizes H2AK119ub and Is Required for Postnatal Development,” Nature Genetics 54 (2022): 625–636. 10.1038/s41588-022-01063-6 PMC9295050 35534561

[59] H. Ouyang , Y. O. Ali , M. Ravichandran , et al., “Protein Aggregates Are Recruited to Aggresome by Histone Deacetylase 6 via Unanchored Ubiquitin C Termini,” Journal of Biological Chemistry 287 (2012): 2317–2327. 10.1074/jbc.M111.273730 PMC3268394 22069321

[60] S. Panier and D. Durocher , “Regulatory Ubiquitylation in Response to DNA Double‐Strand Breaks,” DNA Repair 8 (2009): 436–443. 10.1016/j.dnarep.2009.01.013 19230794

[61] L. Deng , C. Wang , E. Spencer , et al., “Activation of the IκB Kinase Complex by TRAF6 Requires a Dimeric Ubiquitin‐Conjugating Enzyme Complex and a Unique Polyubiquitin Chain,” Cell 103 (2000): 351–361. 10.1016/s0092-8674(00)00126-4 11057907

[62] Y. Haupt , R. Maya , A. Kazaz , et al., “Mdm2 Promotes the Rapid Degradation of p53,” Nature 387 (1997): 296–299. 10.1038/387296a0 9153395

[63] M. Li , D. Chen , A. Shiloh , et al., “Deubiquitination of p53 by HAUSP Is an Important Pathway for p53 Stabilization,” Nature 416 (2002): 648–653. 10.1038/nature737 11923872

[64] Y. Zhao , J. Huang , K. Zhao , et al., “Ubiquitination and Deubiquitination in the Regulation of N6‐Methyladenosine Functional Molecules,” Journal of Molecular Medicine 102 (2024): 337–351. 10.1007/s00109-024-02417-9 38289385

[65] F. Leng , J. Yu , C. Zhang , et al., “Methylated DNMT1 and E2F1 Are Targeted for Proteolysis by L3MBTL3 and CRL4^DCAF5^ Ubiquitin Ligase,” Nature Communications 9 (2018): 1641. 10.1038/s41467-018-04019-9 PMC5915600 29691401

[66] N. Zhang , X. Gao , Q. Yuan , et al., “E3 Ubiquitin Ligase RNF180 Prevents Excessive PCDH10 Methylation to Suppress the Proliferation and Metastasis of Gastric Cancer Cells by Promoting Ubiquitination of DNMT1,” Clinical Epigenetics 15 (2023): 77. 10.1186/s13148-023-01492-y PMC10163782 37147733

[67] S. Wang , L. Zha , X. Cui , et al., “Epigenetic Regulation of Hepatic Lipid Metabolism by DNA Methylation,” Advanced Science 10 (2023): e2206068. 10.1002/advs.202206068 PMC10369300 37282749

[68] Y. H. Huang , C. W. Chen , V. Sundaramurthy , et al., “Systematic Profiling of DNMT3A Variants Reveals Protein Instability Mediated by the DCAF8 E3 Ubiquitin Ligase Adaptor,” Cancer Discovery 12 (2022): 220–235. 10.1158/2159-8290.CD-21-0560 PMC8758508 34429321

[69] K. Jeong , J. M. Murphy , J. H. Kim , et al., “FAK Activation Promotes SMC Dedifferentiation via Increased DNA Methylation in Contractile Genes,” Circulation Research 129 (2021): e215–e233. 10.1161/CIRCRESAHA.121.319066 PMC8639767 34702049

[70] P. Gao , X. Li , S. Zhang , et al., “Regulatory Mechanism of DNMT3A in Interferon Expression Inhibition by Newcastle Disease Virus V Protein,” International Journal of Biological Macromolecules 318 (2025): 144812. 10.1016/j.ijbiomac.2025.144812 40456336

[71] Z. Jin , H. Feng , J. Liang , et al., “FGFR3Δ7–9 Promotes Tumor Progression via the Phosphorylation and Destabilization of Ten‐Eleven Translocation‐2 in Human Hepatocellular Carcinoma,” Cell Death & Disease 11 (2020): 903. 10.1038/s41419-020-03089-2 PMC7584635 33097695

[72] M. Xia , R. Yan , W. Wang , et al., “GID Complex Regulates the Differentiation of Neural Stem Cells by Destabilizing TET2,” Frontiers of Medicine 17 (2023): 1204–1218. 10.1007/s11684-023-1007-9 37707676

[73] S. Fan , J. Wang , G. Yu , et al., “TET Is Targeted for Proteasomal Degradation by the PHD–pVHL Pathway to Reduce DNA Hydroxymethylation,” Journal of Biological Chemistry 295 (2020): 16299–16313. 10.1074/jbc.RA120.014538 PMC7705310 32963106

[74] L. Luo , Z. Zeng , T. Li , et al., “TET2 Stabilized by Deubiquitinase USP21 Ameliorates Cigarette Smoke‐Induced Apoptosis in Airway Epithelial Cells,” Iscience 27 (2024): 109252. 10.1016/j.isci.2024.109252 PMC10910280 38439981

[75] L. Lv , Q. Wang , Y. Xu , et al., “Vpr Targets TET2 for Degradation by CRL4^VprBP^ E3 Ligase to Sustain IL‐6 Expression and Enhance HIV‐1 Replication,” Molecular Cell 70 (2018): 961–970.e5. 10.1016/j.molcel.2018.05.007 PMC6071318 29883611

[76] J. He , Y. Song , G. Li , et al., “Fbxw7 Increases CCL2/7 in CX3CR1hi Macrophages to Promote Intestinal Inflammation,” Journal of Clinical Investigation 129 (2019): 3877–3893. 10.1172/JCI123374 PMC6715366 31246581

[77] H. Liu , E. H. Y. Cheng , and J. J. D. Hsieh , “Bimodal Degradation of MLL by SCF^Skp2^ and APC^Cdc20^ Assures Cell Cycle Execution: A Critical Regulatory Circuit Lost in Leukemogenic MLL Fusions,” Genes & Development 21 (2007): 2385–2398. 10.1101/gad.1574507 PMC1993870 17908926

[78] J. Wang , A. G. Muntean , and J. L. Hess , “ECS^ASB2^ Mediates MLL Degradation During Hematopoietic Differentiation,” Blood 119 (2012): 1151–1161. 10.1182/blood-2011-06-362079 PMC3277351 22174154

[79] K. Liang , A. G. Volk , J. S. Haug , et al., “Therapeutic Targeting of MLL Degradation Pathways in MLL‐Rearranged Leukemia,” Cell 168 (2017): 59–72.e13. 10.1016/j.cell.2016.12.011 PMC5351781 28065413

[80] A. Obri , M. P. Makinistoglu , H. Zhang , et al., “HDAC4 Integrates PTH and Sympathetic Signaling in Osteoblasts,” Journal of Cell Biology 205 (2014): 771–780. 10.1083/jcb.201403138 PMC4068141 24934156

[81] I. Jung , D. H. Sohn , J. Choi , et al., “SRG3/mBAF155 Stabilizes the SWI/SNF‐Like BAF Complex by Blocking CHFR‐Mediated Ubiquitination and Degradation of Its Major Components,” Biochemical and Biophysical Research Communications 418 (2012): 512–517. 10.1016/j.bbrc.2012.01.057 22285184

[82] L. Y. Huang , J. Zhao , H. Chen , et al., “SCF^FBW7^‐Mediated Degradation of BRG1 Suppresses Gastric Cancer Metastasis,” Nature Communications 9 (2018): 3569. 10.1038/s41467-018-06038-y PMC6120942 30177679

[83] Q. Luo , X. Wu , Y. Nan , et al., “TRIM32/USP11 Balances ARID1A Stability and the Oncogenic/Tumor‐Suppressive Status of Squamous Cell Carcinoma,” Cell Reports 30 (2020): 98–111.e5. 10.1016/j.celrep.2019.12.017 31914402

[84] Z. H. Jiang , T. Peng , H. L. Qian , et al., “DNA Damage‐Induced Activation of ATM Promotes β‐TRCP‐Mediated ARID1A Ubiquitination and Destruction in Gastric Cancer Cells,” Cancer Cell International 19 (2019): 162. 10.1186/s12935-019-0878-y PMC6567580 31210753

[85] S. Zhang , Y. F. Zhou , J. Cao , et al., “mTORC1 Promotes ARID1A Degradation and Oncogenic Chromatin Remodeling in Hepatocellular Carcinoma,” Cancer Research 81 (2021): 5652–5665. 10.1158/0008-5472.CAN-21-0206 PMC8595749 34429326

[86] G. Xiao , W. Lu , J. Yuan , et al., “Fbxw7 Suppresses Carcinogenesis and Stemness in Triple‐Negative Breast Cancer Through CHD4 Degradation and Wnt/β‐Catenin Pathway Inhibition,” Journal of Translational Medicine 22 (2024): 99. 10.1186/s12967-024-04897-2 PMC10809768 38268032

[87] J. Luo , S. Xu , J. Wang , et al., “Circular RNA circWBSCR22 Facilitates Colorectal Cancer Metastasis by Enhancing CHD4's Protein Stability,” International Journal of Biological Macromolecules 282 (2024): 137135. 10.1016/j.ijbiomac.2024.137135 39486700

[88] W. Mao , Q. Jiang , Y. Feng , et al., “TRIM21‐Mediated METTL3 Degradation Promotes PDAC Ferroptosis and Enhances the Efficacy of Anti‐PD‐1 Immunotherapy,” Cell Death & Disease 16 (2025): 240. 10.1038/s41419-025-07550-y PMC11965403 40175350

[89] J. Yao , P. Liu , X. Teng , et al., “Alcohol‐Induced Disruption of Hepatic M^6^A Modification Exacerbates Alcohol‐Associated Steatohepatitis by Impairing Liver Immune Microenvironment Homeostasis,” Hepatology 83 (2026): 851–867. 10.1097/HEP.0000000000001374 40304707

[90] Z. Chen , J. Yin , Z. Feng , et al., “O‐GlcNAcylation of METTL3 Drives Hepatocellular Carcinoma Progression by Upregulating MCM10 Expression in an M^6^A‐IGF2BP3‐Dependent Manner,” Cell Death & Disease 16 (2025): 518. 10.1038/s41419-025-07844-1 PMC12255776 40651967

[91] Z. C. Zeng , Q. Pan , Y. M. Sun , et al., “METTL3 Protects METTL14 From STUB1‐Mediated Degradation to Maintain M^6^A Homeostasis,” EMBO Reports 24 (2023): e55762. 10.15252/embr.202255762 PMC9986817 36597993

[92] J. Wei , B. T. Harada , D. Lu , et al., “HRD1‐Mediated METTL14 Degradation Regulates M^6^A mRNA Modification to Suppress ER Proteotoxic Liver Disease,” Molecular Cell 81 (2021): 5052–5065.e6. 10.1016/j.molcel.2021.10.028 PMC8751812 34847358

[93] Y. Ge , T. Ling , Y. Wang , et al., “Degradation of WTAP Blocks Antiviral Responses by Reducing the M^6^A Levels of IRF3 and IFNAR1 mRNA,” EMBO Reports 22 (2021): e52101. 10.15252/embr.202052101 PMC8567250 34467630

[94] K. Lin , E. Zhou , T. Shi , et al., “m^6^A Eraser FTO Impairs Gemcitabine Resistance in Pancreatic Cancer Through Influencing NEDD4 mRNA Stability by Regulating the PTEN/PI3K/AKT Pathway,” Journal of Experimental & Clinical Cancer Research 42 (2023): 217. 10.1186/s13046-023-02792-0 PMC10464189 37605223

[95] Y. H. Cui , J. Wei , H. Fan , et al., “Targeting DTX2/UFD1‐Mediated FTO Degradation to Regulate Antitumor Immunity,” Proceedings of the National Academy of Sciences of the United States of America 121 (2024): e2407910121. 10.1073/pnas.2407910121 PMC11665913 39661064

[96] C. Li , Z. Liu , C. Fu , et al., “Hypoxia‐Induced Degradation of FTO Promotes Apoptosis by Unmasking RACK1‐Mediated Activation of MTK1‐JNK1/2 Pathway,” Journal of Advanced Research 77 (2025): 137–153. 10.1016/j.jare.2025.01.019 PMC12627339 39805423

[97] Y. H. Cui , S. Yang , J. Wei , et al., “Autophagy of the m^6^A mRNA Demethylase FTO Is Impaired by Low‐Level Arsenic Exposure to Promote Tumorigenesis,” Nature Communications 12 (2021): 2183. 10.1038/s41467-021-22469-6 PMC8041927 33846348

[98] G. Chang , G. S. Xie , L. Ma , et al., “USP36 Promotes Tumorigenesis and Drug Sensitivity of Glioblastoma by Deubiquitinating and Stabilizing ALKBH5,” Neuro‐Oncology 25 (2023): 841–853. 10.1093/neuonc/noac238 PMC10158114 36239338

[99] H. Zhang , L. Lu , C. Yi , et al., “BRD4 Regulates M^6^A of ESPL1 mRNA via Interaction With ALKBH5 to Modulate Breast Cancer Progression,” Acta Pharmaceutica Sinica B 15 (2025): 1552–1570. 10.1016/j.apsb.2024.12.037 PMC12069253 40370540

[100] X. Zhou , Q. Xia , B. Wang , et al., “USP14 Modulates Stem‐Like Properties, Tumorigenicity, and Radiotherapy Resistance in Glioblastoma Stem Cells Through Stabilization of MST4‐Phosphorylated ALKBH5,” Theranostics 15 (2025): 2293–2314. 10.7150/thno.103629 PMC11840735 39990235

[101] K. Wang , F. Liu , B. Muchu , et al., “E3 Ubiquitin Ligase RNF180 Mediates the ALKBH5/SMARCA5 Axis to Promote Colon Inflammation and Th17/Treg Imbalance in Ulcerative Colitis Mice,” Archives of Pharmacal Research 47 (2024): 645–658. 10.1007/s12272-024-01507-z 39060657

[102] X. Wang , R. Ling , Y. Peng , et al., “RNPS1 Stabilizes NAT10 Protein to Facilitate Translation in Cancer via tRNA ac^4^C Modification,” International Journal of Oral Science 16 (2024): 6. 10.1038/s41368-023-00276-7 PMC10800354 38246918

[103] Z. Xiao , X. Wei , P. Li , et al., “Impact of N‐Acetyltransferase 10 on Macrophage Activation and Inflammation‐Induced Cardiac Dysfunction,” Cell Death & Disease 16 (2025): 471. 10.1038/s41419-025-07796-6 PMC12216202 40593466

[104] P. Koganti , V. N. Kadali , D. Manikoth Ayyathan , et al., “The E3 Ubiquitin Ligase SMURF2 Stabilizes RNA Editase ADAR1p110 and Promotes Its Adenosine‐To‐Inosine (A‐to‐I) Editing Function,” Cellular and Molecular Life Sciences 79 (2022): 237. 10.1007/s00018-022-04272-8 PMC11072456 35403872

[105] Q. Zhang , B. Xiu , L. Zhang , et al., “Immunosuppressive lncRNA LINC00624 Promotes Tumor Progression and Therapy Resistance Through ADAR1 Stabilization,” Journal for ImmunoTherapy of Cancer 10 (2022): e004666. 10.1136/jitc-2022-004666 PMC9577936 36252997

[106] N. Mailand , S. Bekker‐Jensen , H. Faustrup , et al., “RNF8 Ubiquitylates Histones at DNA Double‐Strand Breaks and Promotes Assembly of Repair Proteins,” Cell 131 (2007): 887–900. 10.1016/j.cell.2007.09.040 18001824

[107] F. Mattiroli , J. H. A. Vissers , W. J. van Dijk , et al., “RNF168 Ubiquitinates K13–15 on H2A/H2AX to Drive DNA Damage Signaling,” Cell 150 (2012): 1182–1195. 10.1016/j.cell.2012.08.005 22980979

[108] C. Hoege , B. Pfander , G. L. Moldovan , et al., “RAD6‐Dependent DNA Repair Is Linked to Modification of PCNA by Ubiquitin and SUMO,” Nature 419 (2002): 135–141. 10.1038/nature00991 12226657

[109] P. L. Kannouche , J. Wing , and A. R. Lehmann , “Interaction of Human DNA Polymerase η With Monoubiquitinated PCNA: A Possible Mechanism for the Polymerase Switch in Response to DNA Damage,” Molecular Cell 14 (2004): 491–500. 10.1016/s1097-2765(04)00259-x 15149598

[110] A. Smogorzewska , S. Matsuoka , P. Vinciguerra , et al., “Identification of the FANCI Protein, a Monoubiquitinated FANCD2 Paralog Required for DNA Repair,” Cell 129 (2007): 289–301. 10.1016/j.cell.2007.03.009 PMC2175179 17412408

[111] V. H. Oestergaard , F. Langevin , H. J. Kuiken , et al., “Deubiquitination of FANCD2 Is Required for DNA Crosslink Repair,” Molecular Cell 28 (2007): 798–809. 10.1016/j.molcel.2007.09.020 PMC2148256 18082605

[112] M. A. Cohn , P. Kowal , K. Yang , et al., “A UAF1‐Containing Multisubunit Protein Complex Regulates the Fanconi Anemia Pathway,” Molecular Cell 28 (2007): 786–797. 10.1016/j.molcel.2007.09.031 18082604

[113] T. T. Huang , S. M. B. Nijman , K. D. Mirchandani , et al., “Regulation of Monoubiquitinated PCNA by DUB Autocleavage,” Nature Cell Biology 8 (2006): 339–347. 10.1038/ncb1378 16531995

[114] P. A. Jones and D. Takai , “The Role of DNA Methylation in Mammalian Epigenetics,” Science 293 (2001): 1068–1070. 10.1126/science.1063852 11498573

[115] A. Hermann , R. Goyal , and A. Jeltsch , “The Dnmt1 DNA‐(Cytosine‐C^5^)‐Methyltransferase Methylates DNA Processively With High Preference for Hemimethylated Target Sites,” Journal of Biological Chemistry 279 (2004): 48350–48359. 10.1074/jbc.M403427200 15339928

[116] M. Okano , D. W. Bell , D. A. Haber , et al., “DNA Methyltransferases Dnmt3a and Dnmt3b Are Essential for De Novo Methylation and Mammalian Development,” Cell 99 (1999): 247–257. 10.1016/s0092-8674(00)81656-6 10555141

[117] M. Tahiliani , K. P. Koh , Y. Shen , et al., “Conversion of ^5^‐Methylcytosine to ^5^‐Hydroxymethylcytosine in Mammalian DNA by MLL Partner TET1,” Science 324 (2009): 930–935. 10.1126/science.1170116 PMC2715015 19372391

[118] S. Ito , L. Shen , Q. Dai , et al., “Tet Proteins Can Convert ^5^‐Methylcytosine to 5‐Formylcytosine and ^5^‐Carboxylcytosine,” Science 333 (2011): 1300–1303. 10.1126/science.1210597 PMC3495246 21778364

[119] S. B. Baylin and P. A. Jones , “A Decade of Exploring the Cancer Epigenome—Biological and Translational Implications,” Nature Reviews Cancer 11 (2011): 726–734. 10.1038/nrc3130 PMC3307543 21941284

[120] M. Bostick , J. K. Kim , P. O. Estève , et al., “UHRF1 Plays a Role in Maintaining DNA Methylation in Mammalian Cells,” Science 317 (2007): 1760–1764. 10.1126/science.1147939 17673620

[121] A. Nishiyama , L. Yamaguchi , J. Sharif , et al., “Uhrf1‐Dependent H3K23 Ubiquitylation Couples Maintenance DNA Methylation and Replication,” Nature 502 (2013): 249–253. 10.1038/nature12488 24013172

[122] W. Qin , P. Wolf , N. Liu , et al., “DNA Methylation Requires a DNMT1 Ubiquitin Interacting Motif (UIM) and Histone Ubiquitination,” Cell Research 25 (2015): 911–929. 10.1038/cr.2015.72 PMC4528052 26065575

[123] S. Ishiyama , A. Nishiyama , Y. Saeki , et al., “Structure of the Dnmt1 Reader Module Complexed With a Unique Two‐Mono‐Ubiquitin Mark on Histone H3 Reveals the Basis for DNA Methylation Maintenance,” Molecular Cell 68 (2017): 350–360.e7. 10.1016/j.molcel.2017.09.037 29053958

[124] A. Nishiyama , C. B. Mulholland , S. Bultmann , et al., “Two Distinct Modes of DNMT1 Recruitment Ensure Stable Maintenance DNA Methylation,” Nature Communications 11 (2020): 1222. 10.1038/s41467-020-15006-4 PMC7060239 32144273

[125] J. Chen , D. Wang , G. Wu , et al., “STUB1‐Mediated K63‐Linked Ubiquitination of UHRF1 Promotes the Progression of Cholangiocarcinoma by Maintaining DNA Hypermethylation of PLA2G2A,” Journal of Experimental & Clinical Cancer Research 43 (2024): 260. 10.1186/s13046-024-03186-6 PMC11395162 39267107

[126] Y. Peng , Y. Liu , R. Zheng , et al., “PLK1 Maintains DNA Methylation and Cell Viability by Regulating Phosphorylation‐Dependent UHRF1 Protein Stability,” Cell Death Discovery 9 (2023): 367. 10.1038/s41420-023-01667-9 PMC10547799 37788997

[127] T. J. Ley , L. Ding , M. J. Walter , et al., “DNMT3A Mutations in Acute Myeloid Leukemia,” New England Journal of Medicine 363 (2010): 2424–2433. 10.1056/NEJMoa1005143 PMC3201818 21067377

[128] D. N. Weinberg , P. Rosenbaum , X. Chen , et al., “Two Competing Mechanisms of DNMT3A Recruitment Regulate the Dynamics of De Novo DNA Methylation at PRC1‐Targeted CpG Islands,” Nature Genetics 53 (2021): 794–800. 10.1038/s41588-021-00856-5 PMC8283687 33986537

[129] D. A. Russler‐Germain , D. H. Spencer , M. A. Young , et al., “The R882H DNMT3A Mutation Associated With AML Dominantly Inhibits Wild‐Type DNMT3A by Blocking Its Ability to Form Active Tetramers,” Cancer Cell 25 (2014): 442–454. 10.1016/j.ccr.2014.02.010 PMC4018976 24656771

[130] Z. Zhang , L. Cheng , Q. Zhang , et al., “Co‐Targeting Plk1 and DNMT3a in Advanced Prostate Cancer,” Advanced Science 8 (2021): e2101458. 10.1002/advs.202101458 PMC8261504 34051063

[131] S. Xu , Z. Yuan , C. Jiang , et al., “DNMT3A Cooperates With YAP/TAZ to Drive Gallbladder Cancer Metastasis,” Advanced Science 11 (2024): e2308531. 10.1002/advs.202308531 PMC11040361 38380551

[132] D. Ye , Y. Wang , X. Deng , et al., “DNMT3a‐Dermatopontin Axis Suppresses Breast Cancer Malignancy via Inactivating YAP,” Cell Death & Disease 14 (2023): 106. 10.1038/s41419-023-05657-8 PMC9922281 36774339

[133] M. Jiang , X. Zhou , Y. Feng , et al., “DNMT3a Promotes LUAD Cell Proliferation and Metastasis by Activating the HDAC7 Signalling Pathway,” International Journal of Biological Sciences 21 (2025): 1585–1602. 10.7150/ijbs.96509 PMC11844290 39990668

[134] Y. Zhao , Y. Zheng , J. Fu , et al., “KDM1A, a Potent and Selective Target, for the Treatment of DNMT3A‐Deficient Non‐Small Cell Lung Cancer,” British Journal of Cancer 131 (2024): 655–667. 10.1038/s41416-024-02772-x PMC11333618 38951697

[135] M. Arroyo , F. D. Hastert , A. Zhadan , et al., “Isoform‐Specific and Ubiquitination Dependent Recruitment of Tet1 to Replicating Heterochromatin Modulates Methylcytosine Oxidation,” Nature Communications 13 (2022): 5173. 10.1038/s41467-022-32799-8 PMC9440122 36056023

[136] L. Jiang , J. Wang , K. Wang , et al., “RNF217 Regulates Iron Homeostasis Through Its E3 Ubiquitin Ligase Activity by Modulating Ferroportin Degradation,” Blood 138 (2021): 689–705. 10.1182/blood.2020008986 PMC8394904 33895792

[137] Q. Yang , H. Dang , J. Liu , et al., “Hypoxia Switches TET1 From Being Tumor‐Suppressive to Oncogenic,” Oncogene 42 (2023): 1634–1648. 10.1038/s41388-023-02659-w PMC10181935 37020036

[138] Z. Zou , X. Dou , Y. Li , et al., “RNA M^5^C Oxidation by TET2 Regulates Chromatin State and Leukaemogenesis,” Nature 634 (2024): 986–994. 10.1038/s41586-024-07969-x PMC11499264 39358506

[139] D. B. Beck , A. Petracovici , C. He , et al., “Delineation of a Human Mendelian Disorder of the DNA Demethylation Machinery: TET3 Deficiency,” American Journal of Human Genetics 106 (2020): 234–245. 10.1016/j.ajhg.2019.12.007 PMC7010978 31928709

[140] A. J. Pulikkottil , S. Bamezai , T. Ammer , et al., “TET3 Promotes AML Growth and Epigenetically Regulates Glucose Metabolism and Leukemic Stem Cell‐Associated Pathways,” Leukemia 36 (2022): 416–425. 10.1038/s41375-021-01390-3 34462525

[141] B. Liu , Y. Dai , Z. Wang , et al., “TET3 Is a Common Epigenetic Immunomodulator of Pathogenic Macrophages,” Journal of Clinical Investigation 135 (2025): e194879. 10.1172/JCI194879 PMC12578387 40794443

[142] J. Jin , X. Xie , Y. Xiao , et al., “Epigenetic Regulation of the Expression of Il12 and Il23 and Autoimmune Inflammation by the Deubiquitinase Trabid,” Nature Immunology 17 (2016): 259–268. 10.1038/ni.3347 PMC4755875 26808229

[143] D. Liu , Z. Zhao , Y. She , et al., “TRIM14 Inhibits OPTN‐Mediated Autophagic Degradation of KDM4D to Epigenetically Regulate Inflammation,” Proceedings of the National Academy of Sciences of the United States of America 119 (2022): e2113454119. 10.1073/pnas.2113454119 PMC8851536 35145029

[144] Z. Zhao , Z. Su , P. Liang , et al., “USP38 Couples Histone Ubiquitination and Methylation via KDM5B to Resolve Inflammation,” Advanced Science 7 (2020): 2002680. 10.1002/advs.202002680 PMC7675183 33240782

[145] J. Wei , S. Dong , R. K. Bowser , et al., “Regulation of the Ubiquitylation and Deubiquitylation of CREB‐Binding Protein Modulates Histone Acetylation and Lung Inflammation,” Science Signaling 10 (2017): eaak9660. 10.1126/scisignal.aak9660 PMC5863726 28611184

[146] Y.‐C. Wang , Y.‐S. Wu , C.‐Y. Hung , et al., “USP24 Induces IL‐6 in Tumor‐Associated Microenvironment by Stabilizing p300 and β‐TrCP and Promotes Cancer Malignancy,” Nature Communications 9 (2018): 3996. 10.1038/s41467-018-06178-1 PMC6162259 30266897

[147] S. Y. Lee , S.‐Y. Park , S.‐H. Lee , et al., “The Deubiquitinase UCHL3 Mediates p300‐Dependent Chemokine Signaling in Alveolar Type II Cells to Promote Pulmonary Fibrosis,” Experimental & Molecular Medicine 55 (2023): 1795–1805. 10.1038/s12276-023-01066-1 PMC10474292 37524875

[148] F. Tang , C. Lu , X. He , et al., “E3 Ligase Trim35 Inhibits LSD1 Demethylase Activity Through K63‐Linked Ubiquitination and Enhances Anti‐Tumor Immunity in NSCLC,” Cell Reports 42 (2023): 113477. 10.1016/j.celrep.2023.113477 37979167

[149] B. Dong , X. Wang , X. Song , et al., “RNF20 Contributes to Epigenetic Immunosuppression Through CDK9‐Dependent LSD1 Stabilization,” Proceedings of the National Academy of Sciences of the United States of America 121 (2024): e2307150121. 10.1073/pnas.2307150121 PMC10873621 38315842

[150] L. Zhang , M. Liu , Y. Xie , et al., “FTO O‐GlcNAcylation Promotes TRIM21‐Mediated FTO Ubiquitination Degradation to Sustain the Negative Feedback Control of Macrophage Inflammation,” Frontiers in Immunology 16 (2025): 1593243. 10.3389/fimmu.2025.1593243 PMC12240770 40642069

[151] H. Liu , X. Zhu , J. Wang , et al., “Mechanisms and Therapeutic Potential of the cGAS‐STING Signaling Pathway in Macrophage Activation and Polarization,” Biochemical Pharmacology 250, no. Pt 2 (2026): 118031. 10.1016/j.bcp.2026.118031 42082120

[152] M. Li , C. L. Brooks , N. Kon , et al., “A Dynamic Role of HAUSP in the p53–Mdm2 Pathway,” Molecular Cell 13 (2004): 879–886. 10.1016/s1097-2765(04)00157-1 15053880

[153] E. Meulmeester , M. M. Maurice , C. Boutell , et al., “Loss of HAUSP‐Mediated Deubiquitination Contributes to DNA Damage‐Induced Destabilization of Hdmx and Hdm2,” Molecular Cell 18 (2005): 565–576. 10.1016/j.molcel.2005.04.024 15916963

[154] J. Yuan , K. Luo , L. Zhang , et al., “USP10 Regulates p53 Localization and Stability by Deubiquitinating p53,” Cell 140 (2010): 384–396. 10.1016/j.cell.2009.12.032 PMC2820153 20096447

[155] J. Y. Ke , C. J. Dai , W. L. Wu , et al., “USP11 Regulates p53 Stability by Deubiquitinating p53,” Journal of Zhejiang University‐SCIENCE B 15 (2014): 1032–1038. 10.1631/jzus.B1400180 PMC4265557 25471832

[156] M. Yada , S. Hatakeyama , T. Kamura , et al., “Phosphorylation‐Dependent Degradation of c‐Myc Is Mediated by the F‐Box Protein Fbw7,” EMBO Journal 23 (2004): 2116–2125. 10.1038/sj.emboj.7600217 PMC424394 15103331

[157] M. Welcker , A. Orian , J. Jin , et al., “The Fbw7 Tumor Suppressor Regulates Glycogen Synthase Kinase 3 Phosphorylation‐Dependent c‐Myc Protein Degradation,” Proceedings of the National Academy of Sciences of the United States of America 101 (2004): 9085–9090. 10.1073/pnas.0402770101 PMC428477 15150404

[158] S. Y. Kim , A. Herbst , K. A. Tworkowski , et al., “Skp2 Regulates Myc Protein Stability and Activity,” Molecular Cell 11 (2003): 1177–1188. 10.1016/s1097-2765(03)00173-4 12769843

[159] N. Popov , M. Wanzel , M. Madiredjo , et al., “The Ubiquitin‐Specific Protease USP28 Is Required for MYC Stability,” Nature Cell Biology 9 (2007): 765–774. 10.1038/ncb1601 17558397

[160] X. X. Sun , X. He , L. Yin , et al., “The Nucleolar Ubiquitin‐Specific Protease USP36 Deubiquitinates and Stabilizes c‐Myc,” Proceedings of the National Academy of Sciences of the United States of America 112 (2015): 3734–3739. 10.1073/pnas.1411713112 PMC4378440 25775507

[161] H. Sutterlüty , E. Chatelain , A. Marti , et al., “p45^SKP2^ Promotes p27^Kip1^ Degradation and Induces S Phase in Quiescent Cells,” Nature Cell Biology 1 (1999): 207–214. 10.1038/12027 10559918

[162] R. Visintin , S. Prinz , and A. Amon , “CDC20 and CDH1: A Family of Substrate‐Specific Activators of APC‐Dependent Proteolysis,” Science 278 (1997): 460–463. 10.1126/science.278.5337.460 9334304

[163] J. M. Peters , “The Anaphase Promoting Complex/Cyclosome: A Machine Designed to Destroy,” Nature Reviews Molecular Cell Biology 7 (2006): 644–656. 10.1038/nrm1988 16896351

[164] Q. Zhong , W. Gao , F. Du , et al., “Mule/ARF‐BP1, a BH3‐Only E3 Ubiquitin Ligase, Catalyzes the Polyubiquitination of Mcl‐1 and Regulates Apoptosis,” Cell 121 (2005): 1085–1095. 10.1016/j.cell.2005.06.009 15989957

[165] M. Schwickart , X. Huang , J. R. Lill , et al., “Deubiquitinase USP9X Stabilizes MCL1 and Promotes Tumour Cell Survival,” Nature 463 (2010): 103–107. 10.1038/nature08646 20023629

[166] Y. Suzuki , Y. Nakabayashi , and R. Takahashi , “Ubiquitin‐Protein Ligase Activity of X‐Linked Inhibitor of Apoptosis Protein Promotes Proteasomal Degradation of Caspase‐3 and Enhances Its Anti‐Apoptotic Effect in Fas‐Induced Cell Death,” Proceedings of the National Academy of Sciences of the United States of America 98 (2001): 8662–8667. 10.1073/pnas.161506698 PMC37492 11447297

[167] M. J. M. Bertrand , S. Milutinovic , K. M. Dickson , et al., “cIAP1 and cIAP2 Facilitate Cancer Cell Survival by Functioning as E3 Ligases That Promote RIP1 Ubiquitination,” Molecular Cell 30 (2008): 689–700. 10.1016/j.molcel.2008.05.014 18570872

[168] J. Wang , X. Yu , W. Gong , et al., “EZH2 Noncanonically Binds cMyc and p300 Through a Cryptic Transactivation Domain to Mediate Gene Activation and Promote Oncogenesis,” Nature Cell Biology 24 (2022): 384–399. 10.1038/s41556-022-00850-x PMC9710513 35210568

[169] L. Wang , C. Chen , Z. Song , et al., “EZH2 Depletion Potentiates MYC Degradation Inhibiting Neuroblastoma and Small Cell Carcinoma Tumor Formation,” Nature Communications 13 (2022): 12. 10.1038/s41467-021-27609-6 PMC8748958 35013218

[170] P. Wang , K. A. Doxtader , and Y. Nam , “Structural Basis for Cooperative Function of Mettl3 and Mettl14 Methyltransferases,” Molecular Cell 63 (2016): 306–317. 10.1016/j.molcel.2016.05.041 PMC4958592 27373337

[171] I. A. Roundtree , M. E. Evans , T. Pan , et al., “Dynamic RNA Modifications in Gene Expression Regulation,” Cell 169 (2017): 1187–1200. 10.1016/j.cell.2017.05.045 PMC5657247 28622506

[172] J. Li , Y. Pei , R. Zhou , et al., “Regulation of RNA N^6^‐Methyladenosine Modification and Its Emerging Roles in Skeletal Muscle Development,” International Journal of Biological Sciences 17 (2021): 1682–1692. 10.7150/ijbs.56251 PMC8120468 33994853

[173] J. Qiu , R. Zhao , C. Ma , et al., “O‐GlcNAcylation Stabilized WTAP Promotes GBM Malignant Progression in an N^6^‐Methyladenosine‐Dependent Manner,” Neuro‐Oncology 27 (2025): 900–915. 10.1093/neuonc/noae268 PMC12083224 39671515

[174] N. Shao , L. Xi , Y. Lv , et al., “USP5 Stabilizes YTHDF1 to Control Cancer Immune Surveillance Through mTORC1‐Mediated Phosphorylation,” Nature Communications 16 (2025): 1313. 10.1038/s41467-025-56564-9 PMC11791202 39900921

[175] Y. Shi , B. Liu , Y. Zhang , et al., “YTHDF1/RNF7/p27 Axis Promotes Prostate Cancer Progression,” Cell Death & Disease 16 (2025): 314. 10.1038/s41419-025-07648-3 PMC12008233 40251202

[176] Q. Fei , Z. Zou , I. A. Roundtree , et al., “YTHDF2 Promotes Mitotic Entry and Is Regulated by Cell‐Cycle Mediators,” PLoS Biology 18 (2020): e3000664. 10.1371/journal.pbio.3000664 PMC7170294 32267835

[177] F. Xu , J. Li , M. Ni , et al., “FBW7 Suppresses Ovarian Cancer Development by Targeting the N^6^‐Methyladenosine‐Binding Protein YTHDF2,” Molecular Cancer 20 (2021): 45. 10.1186/s12943-021-01340-8 PMC7927415 33658012

[178] X. Xu , G. Dai , C. L. Liu , et al., “Fbxo2 Suppresses Prostate Cancer Progression by Regulating YTHDF2 Ubiquitination and Degradation,” Cell Death & Disease 17 (2026): 153. 10.1038/s41419-025-08396-0 PMC12858993 41461633

[179] Y. Yang , Y. Yan , J. Yin , et al., “O‐GlcNAcylation of YTHDF2 Promotes HBV‐Related Hepatocellular Carcinoma Progression in an N^6^‐Methyladenosine‐Dependent Manner,” Signal Transduction and Targeted Therapy 8 (2023): 63. 10.1038/s41392-023-01316-8 PMC9918532 36765030

[180] K. Yu , J. Sun , J. Zhang , et al., “Lactylation Enhances YTHDF3 Stability to Promote Cisplatin Resistance via m^6^A‐Dependent KDM6B Decay in Bladder Cancer,” Cancer Letters 642 (2026): 218282. 10.1016/j.canlet.2026.218282 41621475

[181] N. Sun , S. Wang , J. Liu , et al., “XIAP Promotes Metastasis of Bladder Cancer Cells by Ubiquitylating YTHDC1,” Cell Death & Disease 16 (2025): 205. 10.1038/s41419-025-07545-9 PMC11937301 40133252

[182] B. Yan , X. Li , M. Peng , et al., “The YTHDC1/GLUT3/RNF183 Axis Forms a Positive Feedback Loop That Modulates Glucose Metabolism and Bladder Cancer Progression,” Experimental & Molecular Medicine 55 (2023): 1145–1158. 10.1038/s12276-023-00997-z PMC10318083 37258572

[183] Z. Xing , T. Yang , X. Li , et al., “High‐Glucose‐Associated YTHDC1 Lactylation Reduces the Sensitivity of Bladder Cancer to Enfortumab Vedotin Therapy,” Cell Reports 44 (2025): 115545. 10.1016/j.celrep.2025.115545 40215164

[184] Y. Yang , Y. Zhang , G. Chen , et al., “KAP1 Stabilizes MYCN mRNA and Promotes Neuroblastoma Tumorigenicity by Protecting the RNA m^6^A Reader YTHDC1 Protein Degradation,” Journal of Experimental & Clinical Cancer Research 43 (2024): 141. 10.1186/s13046-024-03040-9 PMC11092262 38745192

[185] M. Gao , Y. Wu , L. Zhang , et al., “YTHDC1 Negatively Regulates UBE3A to Influence RAD51 Ubiquitination and Inhibit Apoptosis in Colorectal Cancer Cells,” Scientific Reports 15 (2025): 8857. 10.1038/s41598-025-92925-6 PMC11909180 40087295

[186] W. P. Li , X. T. Mao , J. H. Xie , et al., “N‐Acetyltransferase 10 Is Implicated in the Pathogenesis of Cycling T Cell‐Mediated Autoimmune and Inflammatory Disorders in Mice,” Nature Communications 15 (2024): 9388. 10.1038/s41467-024-53350-x PMC11525920 39477944

[187] Z. Sun , L. Li , Y. Wu , et al., “Acetylation‐Ubiquitination Crosstalk of DJ‐1 Mediates Microcalcification Formation in Diabetic Plaques via Collagen‐Matrix Vesicles Interaction,” Cardiovascular Research 121 (2025): 296–310. 10.1093/cvr/cvae263 39786474

[188] B. Wang , J. Zhang , G. Li , et al., “N‐Acetyltransferase 10 Promotes Cutaneous Wound Repair via the NF‐κB‐IL‐6 Axis,” Cell Death Discovery 9 (2023): 324. 10.1038/s41420-023-01628-2 PMC10465497 37644005

[189] D. Su , W. Wang , Y. Hou , et al., “Bimodal Regulation of the PRC2 Complex by USP7 Underlies Tumorigenesis,” Nucleic Acids Research 49 (2021): 4421–4440. 10.1093/nar/gkab209 PMC8096222 33849069

[190] A. Yokoyama , F. Ficara , M. J. Murphy , et al., “Proteolytically Cleaved MLL Subunits Are Susceptible to Distinct Degradation Pathways,” Journal of Cell Science 124 (2011): 2208–2219. 10.1242/jcs.080523 PMC3113671 21670200

[191] Q. Wang , M. Yu , Y. Ma , et al., “PHF20L1 Antagonizes SOX2 Proteolysis Triggered by the MLL1/WDR5 Complexes,” Laboratory Investigation 98 (2018): 1627–1641. 10.1038/s41374-018-0106-8 PMC13024356 30089852

[192] W. T. Wang , C. Han , Y. M. Sun , et al., “Activation of the Lysosome‐Associated Membrane Protein LAMP5 by DOT1L Serves as a Bodyguard for MLL Fusion Oncoproteins to Evade Degradation in Leukemia,” Clinical Cancer Research 25 (2019): 2795–2808. 10.1158/1078-0432.CCR-18-1474 30651276

[193] T. Q. Chen , H. J. Huang , S. X. Zhu , et al., “Blockade of the lncRNA‐DOT1L‐LAMP5 Axis Enhances Autophagy and Promotes Degradation of MLL Fusion Proteins,” Experimental Hematology & Oncology 13 (2024): 18. 10.1186/s40164-024-00488-5 PMC10877858 38374003

[194] X. Sun , K. Zhang , X. Peng , et al., “HDAC4‐Mediated LHPP Deacetylation Enhances Its Destabilization and Promotes the Proliferation and Metastasis of Nasopharyngeal Carcinoma,” Cancer Letters 562 (2023): 216158. 10.1016/j.canlet.2023.216158 37023940

[195] W. Xu , Q. Jin , X. Li , et al., “Crosstalk of HDAC4, PP1, and GSDMD in Controlling Pyroptosis,” Cell Death & Disease 15 (2024): 115. 10.1038/s41419-024-06505-z PMC10850491 38326336

[196] W. J. Zang , Y. L. Hu , C. Y. Qian , et al., “HDAC4 Promotes the Growth and Metastasis of Gastric Cancer via Autophagic Degradation of MEKK3,” British Journal of Cancer 127 (2022): 237–248. 10.1038/s41416-022-01805-7 PMC9296555 35637410

[197] Y. Kawaguchi , J. J. Kovacs , A. McLaurin , et al., “The Deacetylase HDAC6 Regulates Aggresome Formation and Cell Viability in Response to Misfolded Protein Stress,” Cell 115 (2003): 727–738. 10.1016/s0092-8674(03)00939-5 14675537

[198] C. Boyault , Y. Zhang , S. Fritah , et al., “HDAC6 Controls Major Cell Response Pathways to Cytotoxic Accumulation of Protein Aggregates,” Genes & Development 21 (2007): 2172–2181. 10.1101/gad.436407 PMC1950856 17785525

[199] J. Y. Lee , H. Koga , Y. Kawaguchi , et al., “HDAC6 Controls Autophagosome Maturation Essential for Ubiquitin‐Selective Quality‐Control Autophagy,” EMBO Journal 29 (2010): 969–980. 10.1038/emboj.2009.405 PMC2837169 20075865

[200] Z. Y. Li , C. Zhang , Y. Zhang , et al., “A Novel HDAC6 Inhibitor Tubastatin A: Controls HDAC6‐p97/VCP‐Mediated Ubiquitination‐Autophagy Turnover and Reverses Temozolomide‐Induced ER Stress‐Tolerance in GBM Cells,” Cancer Letters 391 (2017): 89–99. 10.1016/j.canlet.2017.01.025 28131906

[201] Z. You , W. X. Jiang , L. Y. Qin , et al., “Requirement for p62 Acetylation in the Aggregation of Ubiquitylated Proteins Under Nutrient Stress,” Nature Communications 10 (2019): 5792. 10.1038/s41467-019-13718-w PMC6923396 31857589

[202] Y. Lee , T. F. Chou , S. K. Pittman , et al., “Keap1/Cullin3 Modulates p62/SQSTM1 Activity via UBA Domain Ubiquitination,” Cell Reports 19 (2017): 188–202. 10.1016/j.celrep.2017.03.030 PMC5395095 28380357

[203] Y. Deng , J. Gao , G. Xu , et al., “HDAC6‐Dependent Deacetylation of AKAP12 Dictates Its Ubiquitination and Promotes Colon Cancer Metastasis,” Cancer Letters 549 (2022): 215911. 10.1016/j.canlet.2022.215911 36122629

[204] X. Han , B. Gui , C. Xiong , et al., “Destabilizing LSD1 by Jade‐2 Promotes Neurogenesis: An Antibraking System in Neural Development,” Molecular Cell 55 (2014): 482–494. 10.1016/j.molcel.2014.06.006 25018020

[205] B. Dong , X. Song , X. Wang , et al., “FBXO24 Suppresses Breast Cancer Tumorigenesis by Targeting LSD1 for Ubiquitination,” Molecular Cancer Research 21 (2023): 1303–1316. 10.1158/1541-7786.MCR-23-0169 PMC10840093 37540490

[206] L. Yi , Y. Cui , Q. Xu , et al., “Stabilization of LSD1 by Deubiquitinating Enzyme USP7 Promotes Glioblastoma Cell Tumorigenesis and Metastasis Through Suppression of the p53 Signaling Pathway,” Oncology Reports 36 (2016): 2935–2945. 10.3892/or.2016.5099 27632941

[207] A. Zhou , K. Lin , S. Zhang , et al., “Nuclear GSK3β Promotes Tumorigenesis by Phosphorylating KDM1A and Inducing Its Deubiquitylation by USP22,” Nature Cell Biology 18 (2016): 954–966. 10.1038/ncb3396 PMC5026327 27501329

[208] Y. Wu , Y. Wang , X. H. Yang , et al., “The Deubiquitinase USP28 Stabilizes LSD1 and Confers Stem‐Cell‐Like Traits to Breast Cancer Cells,” Cell Reports 5 (2013): 224–236. 10.1016/j.celrep.2013.08.030 PMC4004762 24075993

[209] J. Liu , J. Feng , L. Li , et al., “Arginine Methylation‐Dependent LSD1 Stability Promotes Invasion and Metastasis of Breast Cancer,” EMBO Reports 21 (2020): e48597. 10.15252/embr.201948597 PMC7001506 31833203

[210] A. Li , Z. Gong , Y. Long , et al., “Lactylation of LSD1 Is an Acquired Epigenetic Vulnerability of BRAFi/MEKi‐Resistant Melanoma,” Developmental Cell 60 (2025): 1974–1990.e11. 10.1016/j.devcel.2025.02.016 40132584

[211] Z. Gong , A. Li , J. Ding , et al., “OTUD7B Deubiquitinates LSD1 to Govern Its Binding Partner Specificity, Homeostasis, and Breast Cancer Metastasis,” Advanced Science 8 (2021): e2004504. 10.1002/advs.202004504 PMC8336515 34050636

[212] M. K. M. Tan , H. J. Lim , and J. W. Harper , “SCF^FBXO22^ Regulates Histone H3 Lysine 9 and 36 Methylation Levels by Targeting Histone Demethylase KDM4A for Ubiquitin‐Mediated Proteasomal Degradation,” Molecular and Cellular Biology 31 (2011): 3687–3699. 10.1128/MCB.05746-11 PMC3165736 21768309

[213] C. Van Rechem , J. C. Black , T. Abbas , et al., “The SKP1–CUL1–F‐Box and Leucine‐Rich Repeat Protein 4 (SCF‐FbxL4) Ubiquitin Ligase Regulates Lysine Demethylase 4A (KDM4A)/Jumonji Domain‐Containing 2A (JMJD2A) Protein,” Journal of Biological Chemistry 286 (2011): 30462–30470. 10.1074/jbc.M111.273508 PMC3162406 21757720

[214] F. A. Mallette , F. Mattiroli , G. Cui , et al., “RNF8‐ and RNF168‐Dependent Degradation of KDM4A/JMJD2A Triggers 53BP1 Recruitment to DNA Damage Sites,” EMBO Journal 31 (2012): 1865–1878. 10.1038/emboj.2012.47 PMC3343333 22373579

[215] H. Yang , X. Liu , J. Nie , et al., “USP7 Promotes Chemotherapy Resistance and DNA Damage Response Through Stabilizing and Deubiquitinating KDM4A in Bladder Cancer,” Cell Death & Disease 17 (2026): 123. 10.1038/s41419-025-08297-2 PMC12847834 41429764

[216] C. R. Clapier , J. Iwasa , B. R. Cairns , et al., “Mechanisms of Action and Regulation of ATP‐Dependent Chromatin‐Remodelling Complexes,” Nature Reviews Molecular Cell Biology 18 (2017): 407–422. 10.1038/nrm.2017.26 PMC8127953 28512350

[217] C. Kadoch , D. C. Hargreaves , C. Hodges , et al., “Proteomic and Bioinformatic Analysis of Mammalian SWI/SNF Complexes Identifies Extensive Roles in Human Malignancy,” Nature Genetics 45 (2013): 592–601. 10.1038/ng.2628 PMC3667980 23644491

[218] C. Kadoch and G. R. Crabtree , “Mammalian SWI/SNF Chromatin Remodeling Complexes and Cancer: Mechanistic Insights Gained From Human Genomics,” Science Advances 1 (2015): e1500447. 10.1126/sciadv.1500447 PMC4640607 26601204

[219] Y. Wang , N. Zhang , L. Zhang , et al., “Autophagy Regulates Chromatin Ubiquitination in DNA Damage Response Through Elimination of SQSTM1/p62,” Molecular Cell 63 (2016): 34–48. 10.1016/j.molcel.2016.05.027 27345151

[220] A. R. Barutcu , B. R. Lajoie , A. J. Fritz , et al., “SMARCA4 Regulates Gene Expression and Higher‐Order Chromatin Structure in Proliferating Mammary Epithelial Cells,” Genome Research 26 (2016): 1188–1201. 10.1101/gr.201624.115 PMC5052043 27435934

[221] J. E. Lazar , S. Stehling‐Sun , V. Nandakumar , et al., “Global Regulatory DNA Potentiation by SMARCA4 Propagates to Selective Gene Expression Programs via Domain‐Level Remodeling,” Cell Reports 31 (2020): 107676. 10.1016/j.celrep.2020.107676 32460018

[222] S. Kassel , K. Yuan , N. Bunnag , et al., “The TRIP12 E3 Ligase Induces SWI/SNF Component BRG1–β‐Catenin Interaction to Promote Wnt Signaling,” Nature Communications 16 (2025): 5248. 10.1038/s41467-025-60535-5 PMC12141612 40473626

[223] M. Yates , I. Marois , E. St‐Hilaire , et al., “SMARCAL1 Ubiquitylation Controls Its Association With RPA‐Coated ssDNA and Promotes Replication Fork Stability,” PLoS Biology 22 (2024): e3002552. 10.1371/journal.pbio.3002552 PMC10950228 38502677

[224] R. Mathur , B. H. Alver , A. K. San Roman , et al., “ARID1A Loss Impairs Enhancer‐Mediated Gene Regulation and Drives Colon Cancer in Mice,” Nature Genetics 49 (2017): 296–302. 10.1038/ng.3744 PMC5285448 27941798

[225] L. Farnung , M. Ochmann , and P. Cramer , “Nucleosome‐CHD4 Chromatin Remodeler Structure Maps Human Disease Mutations,” Elife 9 (2020): e56178. 10.7554/eLife.56178 PMC7338049 32543371

[226] C. L. Chang , C. R. Huang , S. J. Chang , et al., “CHD4 as an Important Mediator in Regulating the Malignant Behaviors of Colorectal Cancer,” International Journal of Biological Sciences 17 (2021): 1660–1670. 10.7150/ijbs.56976 PMC8120460 33994851

[227] J. Wu , Z. Zhou , J. Li , et al., “CHD4 Promotes Acquired Chemoresistance and Tumor Progression by Activating the MEK/ERK Axis,” Drug Resistance Updates 66 (2023): 100913. 10.1016/j.drup.2022.100913 36603431

[228] Y. Shi , Z. Zhao , S. Zhou , et al., “CHD4 Drives Gastric Cancer Metastasis via MYH9/GSK3β/β‐Catenin Axis and WNT/EMT Pathway Activation,” Cancer Letters 628 (2025): 217813. 10.1016/j.canlet.2025.217813 40480417

[229] J. Gong , X. Yao , T. Shang , et al., “Molecular Mechanisms of Skeletal Muscle Atrophy: Clinical Challenges and Future Therapeutic Strategies,” Biochemical Pharmacology 249 (2026): 117913. 10.1016/j.bcp.2026.117913 41865897

[230] J. Yi , J. Zhang , X. Fang , et al., “RIPK3 Deficiency Ameliorates Diabetic Sarcopenia Through Proteostasis Regulation by Suppressing Inflammatory Signaling and Cellular Stress Pathways,” Biochemical Pharmacology 244 (2026): 117627. 10.1016/j.bcp.2025.117627 41380803

[231] M. S. Hipp , P. Kasturi , and F. U. Hartl , “The Proteostasis Network and Its Decline in Ageing,” Nature Reviews Molecular Cell Biology 20 (2019): 421–435. 10.1038/s41580-019-0101-y 30733602

[232] J. Labbadia and R. I. Morimoto , “The Biology of Proteostasis in Aging and Disease,” Annual Review of Biochemistry 84 (2015): 435–464. 10.1146/annurev-biochem-060614-033955 PMC4539002 25784053

[233] P. Sweeney , H. Park , M. Baumann , et al., “Protein Misfolding in Neurodegenerative Diseases: Implications and Strategies,” Translational Neurodegeneration 6 (2017): 6. 10.1186/s40035-017-0077-5 PMC5348787 28293421

[234] T. Kitada , S. Asakawa , N. Hattori , et al., “Mutations in the Parkin Gene Cause Autosomal Recessive Juvenile Parkinsonism,” Nature 392 (1998): 605–608. 10.1038/33416 9560156

[235] E. M. Valente , P. M. Abou‐Sleiman , V. Caputo , et al., “Hereditary Early‐Onset Parkinson's Disease Caused by Mutations in PINK1,” Science 304 (2004): 1158–1160. 10.1126/science.1096284 15087508

[236] D. Narendra , A. Tanaka , D. F. Suen , et al., “Parkin Is Recruited Selectively to Impaired Mitochondria and Promotes Their Autophagy,” Journal of Cell Biology 183 (2008): 795–803. 10.1083/jcb.200809125 PMC2592826 19029340

[237] D. P. Narendra , S. M. Jin , A. Tanaka , et al., “PINK1 Is Selectively Stabilized on Impaired Mitochondria to Activate Parkin,” PLoS Biology 8 (2010): e1000298. 10.1371/journal.pbio.1000298 PMC2811155 20126261

[238] F. Koyano , K. Okatsu , H. Kosako , et al., “Ubiquitin Is Phosphorylated by PINK1 to Activate Parkin,” Nature 510 (2014): 162–166. 10.1038/nature13392 24784582

[239] M. Lazarou , D. A. Sliter , L. A. Kane , et al., “The Ubiquitin Kinase PINK1 Recruits Autophagy Receptors to Induce Mitophagy,” Nature 524 (2015): 309–314. 10.1038/nature14893 PMC5018156 26266977

[240] J. M. Heo , A. Ordureau , J. A. Paulo , et al., “The PINK1–PARKIN Mitochondrial Ubiquitylation Pathway Drives a Program of OPTN/NDP52 Recruitment and TBK1 Activation to Promote Mitophagy,” Molecular Cell 60 (2015): 7–20. 10.1016/j.molcel.2015.08.016 PMC4592482 26365381

[241] B. Bingol , J. S. Tea , L. Phu , et al., “The Mitochondrial Deubiquitinase USP30 Opposes Parkin‐Mediated Mitophagy,” Nature 510 (2014): 370–375. 10.1038/nature13418 24896179

[242] J. Okarmus , J. B. Agergaard , T. C. Stummann , et al., “USP30 Inhibition Induces Mitophagy and Reduces Oxidative Stress in Parkin‐Deficient Human Neurons,” Cell Death & Disease 15 (2024): 52. 10.1038/s41419-024-06439-6 PMC10789816 38225227

[243] M. Bordoni , E. Scarian , E. Jacchetti , et al., “HDAC6 and TDP‐43 Promote Autophagy Impairment in Amyotrophic Lateral Sclerosis,” Neurobiology of Disease 215 (2025): 107079. 10.1016/j.nbd.2025.107079 40912409

[244] A. Iwata , B. E. Riley , J. A. Johnston , et al., “HDAC6 and Microtubules Are Required for Autophagic Degradation of Aggregated Huntingtin,” Journal of Biological Chemistry 280 (2005): 40282–40292. 10.1074/jbc.M508786200 16192271

[245] U. B. Pandey , Z. Nie , Y. Batlevi , et al., “HDAC6 Rescues Neurodegeneration and Provides an Essential Link Between Autophagy and the UPS,” Nature 447 (2007): 859–863. 10.1038/nature05853 17568747

[246] A. Qi , K. Wang , Y. Li , et al., “The Degradation of α‐Synuclein Is Limited by Dynein to Drive the AALP Pathway Through HDAC6 Upon Paraquat Exposure,” Ecotoxicology and Environmental Safety 283 (2024): 116841. 10.1016/j.ecoenv.2024.116841 39128448

[247] H. X. Deng , W. Chen , S. T. Hong , et al., “Mutations in UBQLN2 Cause Dominant X‐Linked Juvenile and Adult‐Onset ALS and ALS/Dementia,” Nature 477 (2011): 211–215. 10.1038/nature10353 PMC3169705 21857683

[248] E. Rubino , I. Rainero , A. Chiò , et al., “SQSTM1 Mutations in Frontotemporal Lobar Degeneration and Amyotrophic Lateral Sclerosis,” Neurology 79 (2012): 1556–1562. 10.1212/WNL.0b013e31826e25df PMC3655323 22972638

[249] H. Maruyama , H. Morino , H. Ito , et al., “Mutations of Optineurin in Amyotrophic Lateral Sclerosis,” Nature 465 (2010): 223–226. 10.1038/nature08971 20428114

[250] A. Freischmidt , T. Wieland , B. Richter , et al., “Haploinsufficiency of TBK1 Causes Familial ALS and Fronto‐Temporal Dementia,” Nature Neuroscience 18 (2015): 631–636. 10.1038/nn.4000 25803835

[251] M. Gonzalez‐Zuñiga , P. S. Contreras , L. D. Estrada , et al., “c‐Abl Stabilizes HDAC2 Levels by Tyrosine Phosphorylation Repressing Neuronal Gene Expression in Alzheimer's Disease,” Molecular Cell 56 (2014): 163–173. 10.1016/j.molcel.2014.08.013 25219501

[252] X. Li , T. Liu , T.‐T. Wu , et al., “SIRT1 Deacetylates TET2 and Promotes Its Ubiquitination Degradation to Achieve Neuroprotection Against Parkinson's Disease,” Frontiers in Neurology 12 (2021): 652882. 10.3389/fneur.2021.652882 PMC8082066 33935952

[253] Y. Wei , J. Wang , Y. Ji , et al., “Therapeutic Challenges and Future Breakthroughs in Amyotrophic Lateral Sclerosis: From Precision Medicine to Innovative Trial Design,” Drug Design, Development and Therapy 20 (2026): 626069. 10.2147/DDDT.S626069 PMC13522104 42666355

[254] X. Chen , Z. Zhao , X. Yao , et al., “The Central Role of Endoplasmic Reticulum Stress in Parkinson's Disease and Targeted Therapeutic Strategies,” Biochemical Pharmacology 252 (2026): 118218. 10.1016/j.bcp.2026.118218 42398868

[255] Q. Zhou , H. Wang , D. M. Schwartz , et al., “Loss‐Of‐Function Mutations in TNFAIP3 Leading to A20 Haploinsufficiency Cause an Early‐Onset Autoinflammatory Disease,” Nature Genetics 48 (2016): 67–73. 10.1038/ng.3459 PMC4777523 26642243

[256] Q. Zhou , X. Yu , E. Demirkaya , et al., “Biallelic Hypomorphic Mutations in a Linear Deubiquitinase Define Otulipenia, an Early‐Onset Autoinflammatory Disease,” Proceedings of the National Academy of Sciences of the United States of America 113 (2016): 10127–10132. 10.1073/pnas.1612594113 PMC5018768 27559085

[257] B. Boisson , E. Laplantine , C. Prando , et al., “Immunodeficiency, Autoinflammation and Amylopectinosis in Humans With Inherited HOIL‐1 and LUBAC Deficiency,” Nature Immunology 13 (2012): 1178–1186. 10.1038/ni.2457 PMC3514453 23104095

[258] B. Boisson , E. Laplantine , K. Dobbs , et al., “Human HOIP and LUBAC Deficiency Underlies Autoinflammation, Immunodeficiency, Amylopectinosis, and Lymphangiectasia,” Journal of Experimental Medicine 212 (2015): 939–951. 10.1084/jem.20141130 PMC4451137 26008899

[259] Y. Liu , A. A. Jesus , B. Marrero , et al., “Activated STING in a Vascular and Pulmonary Syndrome,” New England Journal of Medicine 371 (2014): 507–518. 10.1056/NEJMoa1312625 PMC4174543 25029335

[260] H. Song , B. Liu , W. Huai , et al., “The E3 Ubiquitin Ligase TRIM31 Attenuates NLRP3 Inflammasome Activation by Promoting Proteasomal Degradation of NLRP3,” Nature Communications 7 (2016): 13727. 10.1038/ncomms13727 PMC5155141 27929086

[261] B. H. Duong , M. Onizawa , J. A. Oses‐Prieto , et al., “A20 Restricts Ubiquitination of Pro‐Interleukin‐1β Protein Complexes and Suppresses NLRP3 Inflammasome Activity,” Immunity 42 (2015): 55–67. 10.1016/j.immuni.2014.12.031 PMC4302274 25607459

[262] H. M. Hoffman , J. L. Mueller , D. H. Broide , et al., “Mutation of a New Gene Encoding a Putative Pyrin‐Like Protein Causes Familial Cold Autoinflammatory Syndrome and Muckle–Wells Syndrome,” Nature Genetics 29 (2001): 301–305. 10.1038/ng756 PMC4322000 11687797

[263] B. J. Liddicoat , R. Piskol , A. M. Chalk , et al., “RNA Editing by ADAR1 Prevents MDA5 Sensing of Endogenous dsRNA as Nonself,” Science 349 (2015): 1115–1120. 10.1126/science.aac7049 PMC5444807 26275108

[264] G. I. Rice , P. R. Kasher , G. M. A. Forte , et al., “Mutations in ADAR1 Cause Aicardi–Goutières Syndrome Associated With a Type I Interferon Signature,” Nature Genetics 44 (2012): 1243–1248. 10.1038/ng.2414 PMC4154508 23001123

[265] D. P. Bondeson , A. Mares , I. E. D. Smith , et al., “Catalytic in Vivo Protein Knockdown by Small‐Molecule PROTACs,” Nature Chemical Biology 11 (2015): 611–617. 10.1038/nchembio.1858 PMC4629852 26075522

[266] M. S. Gadd , A. Testa , X. Lucas , et al., “Structural Basis of PROTAC Cooperative Recognition for Selective Protein Degradation,” Nature Chemical Biology 13 (2017): 514–521. 10.1038/nchembio.2329 PMC5392356 28288108

[267] T. Han , M. Goralski , N. Gaskill , et al., “Anticancer Sulfonamides Target Splicing by Inducing RBM39 Degradation via Recruitment to DCAF15,” Science 356 (2017): eaal3755. 10.1126/science.aal3755 28302793

[268] T. Uehara , Y. Minoshima , K. Sagane , et al., “Selective Degradation of Splicing Factor CAPERα by Anticancer Sulfonamides,” Nature Chemical Biology 13 (2017): 675–680. 10.1038/nchembio.2363 28437394

[269] J. Cantley , X. Ye , E. Rousseau , et al., “Selective PROTAC‐Mediated Degradation of SMARCA2 Is Efficacious in SMARCA4 Mutant Cancers,” Nature Communications 13 (2022): 6814. 10.1038/s41467-022-34562-5 PMC9649729 36357397

[270] H. Lv , B. Liu , Y. Dai , et al., “TET3‐Overexpressing Macrophages Promote Endometriosis,” Journal of Clinical Investigation 134 (2024): e181839. 10.1172/JCI181839 PMC11527447 39141428

[271] A. P. Turnbull , S. Ioannidis , W. W. Krajewski , et al., “Molecular Basis of USP7 Inhibition by Selective Small Molecule Inhibitors,” Nature 550 (2017): 481–486. 10.1038/nature24451 PMC6029662 29045389

[272] L. Cadzow , J. Brenneman , E. Tobin , et al., “The USP1 Inhibitor KSQ‐4279 Overcomes PARP Inhibitor Resistance in Homologous Recombination‐Deficient Tumors,” Cancer Research 84 (2024): 3419–3434. 10.1158/0008-5472.CAN-24-0293 PMC11474170 39402989

[273] E. K. Rowinsky , A. Paner , J. G. Berdeja , et al., “Phase 1 Study of the Protein Deubiquitinase Inhibitor VLX1570 in Patients With Relapsed and/or Refractory Multiple Myeloma,” Investigational New Drugs 38 (2020): 1448–1453. 10.1007/s10637-020-00915-4 PMC7497669 32125598

[274] L. T. Vassilev , B. T. Vu , B. Graves , et al., “In Vivo Activation of the p53 Pathway by Small‐Molecule Antagonists of MDM2,” Science 303 (2004): 844–848. 10.1126/science.1092472 14704432

[275] M. Y. Konopleva , C. Röllig , J. Cavenagh , et al., “Idasanutlin Plus Cytarabine in Relapsed or Refractory Acute Myeloid Leukemia: Results of the MIRROS Trial,” Blood Advances 6 (2022): 4147–4156. 10.1182/bloodadvances.2021006303 PMC9327534 35413116

[276] E. Varfolomeev , J. W. Blankenship , S. M. Wayson , et al., “IAP Antagonists Induce Autoubiquitination of c‐IAPs, NFκB Activation, and TNFα‐Dependent Apoptosis,” Cell 131 (2007): 669–681. 10.1016/j.cell.2007.10.030 18022362

[277] J. N. Spradlin , X. Hu , C. C. Ward , et al., “Harnessing the Anti‐Cancer Natural Product Nimbolide for Targeted Protein Degradation,” Nature Chemical Biology 15 (2019): 747–755. 10.1038/s41589-019-0304-8 PMC6592714 31209351

[278] X. Zhang , V. M. Crowley , T. G. Wucherpfennig , et al., “Electrophilic PROTACs That Degrade Nuclear Proteins by Engaging DCAF16,” Nature Chemical Biology 15 (2019): 737–746. 10.1038/s41589-019-0279-5 PMC6592777 31209349

[279] X. Zhang , L. M. Luukkonen , C. L. Eissler , et al., “DCAF11 Supports Targeted Protein Degradation by Electrophilic Proteolysis Targeting Chimeras,” Journal of the American Chemical Society 143 (2021): 5141–5149. 10.1021/jacs.1c00990 PMC8309050 33783207

[280] N. J. Henning , A. G. Manford , J. N. Spradlin , et al., “Discovery of a Covalent FEM1B Recruiter for Targeted Protein Degradation Applications,” Journal of the American Chemical Society 144 (2022): 701–708. 10.1021/jacs.1c03980 PMC8928484 34994556

[281] M. Campone , M. De Laurentiis , K. Jhaveri , et al., “Vepdegestrant, a PROTAC Estrogen Receptor Degrader, in Advanced Breast Cancer,” New England Journal of Medicine 393 (2025): 556–568. 10.1056/NEJMoa2505725 40454645

[282] J. A. Livingston , J.‐Y. Blay , J. Trent , et al., “A Phase I Study of FHD‐609, a Heterobifunctional Degrader of Bromodomain‐Containing Protein 9, in Patients With Advanced Synovial Sarcoma or SMARCB1‐Deficient Tumors,” Clinical Cancer Research 31 (2025): 628–638. 10.1158/1078-0432.CCR-24-2583 39660994

[283] L. Ackerman , G. Acloque , S. Bacchelli , et al., “IRAK4 Degrader in Hidradenitis Suppurativa and Atopic Dermatitis: A Phase 1 Trial,” Nature Medicine 29 (2023): 3127–3136. 10.1038/s41591-023-02635-7 PMC10719089 37957373

[284] G. Qi , F. Xue , H. Sun , et al., “The Dual Role of the cGAS‐STING Signaling Pathway in Kidney Diseases: From Acute Injury to Chronic Fibrosis—Molecular Mechanisms and Precision Therapeutic Strategies,” Frontiers in Pharmacology 17 (2026): 1882994. 10.3389/fphar.2026.1882994 PMC13485976 42621049

[285] L. Zhang , B. Riley‐Gillis , P. Vijay , et al., “Acquired Resistance to BET‐PROTACs (Proteolysis‐Targeting Chimeras) Caused by Genomic Alterations in Core Components of E3 Ligase Complexes,” Molecular Cancer Therapeutics 18 (2019): 1302–1311. 10.1158/1535-7163.MCT-18-1129 31064868

[286] P. Ottis , C. Palladino , P. Thienger , et al., “Cellular Resistance Mechanisms to Targeted Protein Degradation Converge Toward Impairment of the Engaged Ubiquitin‐Transfer Pathway,” ACS Chemical Biology 14 (2019): 2215–2223. 10.1021/acschembio.9b00525 31553577

[287] C. Mayor‐Ruiz , M. G. Jaeger , S. Bauer , et al., “Plasticity of the Cullin–RING Ligase Repertoire Shapes Sensitivity to Ligand‐Induced Protein Degradation,” Molecular Cell 75 (2019): 849–858.e8. 10.1016/j.molcel.2019.07.013 31442425

[288] N. D. Udeshi , P. Mertins , T. Svinkina , et al., “Large‐Scale Identification of Ubiquitination Sites by Mass Spectrometry,” Nature Protocols 8 (2013): 1950–1960. 10.1038/nprot.2013.120 PMC4725055 24051958

[289] Y. Leestemaker , A. de Jong , and H. Ovaa , “Profiling the Activity of Deubiquitinating Enzymes Using Chemically Synthesized Ubiquitin‐Based Probes,” Methods in Molecular Biology 1491 (2017): 113–130. 10.1007/978-1-4939-6439-0_9 27778285

[290] R. T. Timms , E. L. Mena , Y. Leng , et al., “Defining E3 Ligase–Substrate Relationships Through Multiplex CRISPR Screening,” Nature Cell Biology 25 (2023): 1535–1545. 10.1038/s41556-023-01229-2 PMC10567573 37735597

[291] T. M. Tessier , M. E. R. Maitland , M. Kutera , et al., “Identification of E3 Ligase Substrates and PROTAC‐Induced Ubiquitylation Sites Using Proximity‐Based Identification of Ubiquitin Sites (PrIUS),” Communications Biology 9 (2026): 781. 10.1038/s42003-026-09964-6 PMC13250050 41957433

[292] L. T. Henneberg , J. Singh , D. M. Duda , et al., “Activity‐Based Profiling of Cullin–RING E3 Networks by Conformation‐Specific Probes,” Nature Chemical Biology 19 (2023): 1513–1523. 10.1038/s41589-023-01392-5 PMC10667097 37653169

[293] N. K. Chua , T. J. González‐Robles , C. J. Reddington , et al., “The E3‐Ome Gene‐Centric Compendium Reveals the Human E3 Ligase Landscape,” Cell 189 (2026): 2167–2188.e27. 10.1016/j.cell.2026.01.029 PMC13061254 41864206

[294] M. Schröder , M. Renatus , X. Liang , et al., “DCAF1‐Based PROTACs With Activity Against Clinically Validated Targets Overcoming Intrinsic‐ and Acquired‐Degrader Resistance,” Nature Communications 15 (2024): 275. 10.1038/s41467-023-44237-4 PMC10766610 38177131

[295] M. F. Mabanglo , B. Wilson , M. Noureldin , et al., “Crystal Structures of DCAF1–PROTAC–WDR5 Ternary Complexes Provide Insight Into DCAF1 Substrate Specificity,” Nature Communications 15 (2024): 10165. 10.1038/s41467-024-54500-x PMC11585590 39580491

